# Molecular mechanism of transition-state inhibitors of bacterial antibiotic efflux pumps

**DOI:** 10.1038/s44259-026-00207-6

**Published:** 2026-05-04

**Authors:** Clara Börnsen, Reinke T. Müller, Anais Vieira Da Cruz, Juan-Carlos Jiménez-Castellanos, Virginie Meurillon, Lorenz Brandstätter, Eszter V. Lodinsky, Mohd Athar, Attilio V. Vargiu, Ruben C. Hartkoorn, Marion Flipo, Achilleas S. Frangakis, Klaas M. Pos

**Affiliations:** 1 Buchmann Institute for Molecular Life Sciences and Institute for Biophysics, Goethe University Frankfurt, Frankfurt am Main, Germany; 2 Cluster of Excellence SubCellular Architecture of Life (SCALE), Goethe University, Frankfurt, Frankfurt am Main, Germany; 3 Institute of Biochemistry, Goethe-University Frankfurt, Frankfurt am Main, Germany; 4 Univ. Lille, Inserm, Institut Pasteur de Lille, U1177 - Drugs and Molecules for Living Systems, F-59000 Lille, France; 5 Univ. Lille, CNRS, Inserm, CHU Lille, Institut Pasteur de Lille, U1019 - UMR 9017 - CIIL - Center for Infection and Immunity of Lille, F-59000 Lille, France; 6 Department of Physics, University of Cagliari, Cagliari, Italy

**Keywords:** Biochemistry, Drug discovery, Microbiology

## Abstract

In many Gram-negative bacteria such as *Escherichia coli* and *Klebsiella pneumoniae*, the AcrAB-TolC efflux pump is central to multidrug resistance. We report the development of BDM91531, a nanomolar pyridylpiperazine inhibitor that potentiates the activity of several antibiotics. Structural analyses by X-ray crystallography and cryo-EM revealed that the divalent cationic BDM91531 binds AcrB through electrostatic interactions with a central role for residues D408 and E947, trapping protomers in an O to L transitional state and blocking the conformational cycling of the trimer. Differential scanning fluorimetry and susceptibility tests confirmed this inhibitory mechanism. Negative charges at the cytoplasmic rim are essential for inhibitor uptake as electrostatic attraction from rim carboxylates, including E947 and D951, facilitates entry. Loss of D951 abolished inhibitor sensitivity, whereas introducing alternative negative charges restored activity. These findings establish BDM91531 as a potent AcrB efflux pump inhibitor and highlight structural determinants for inhibitor access and binding.

## Introduction

**Fig. 1:**
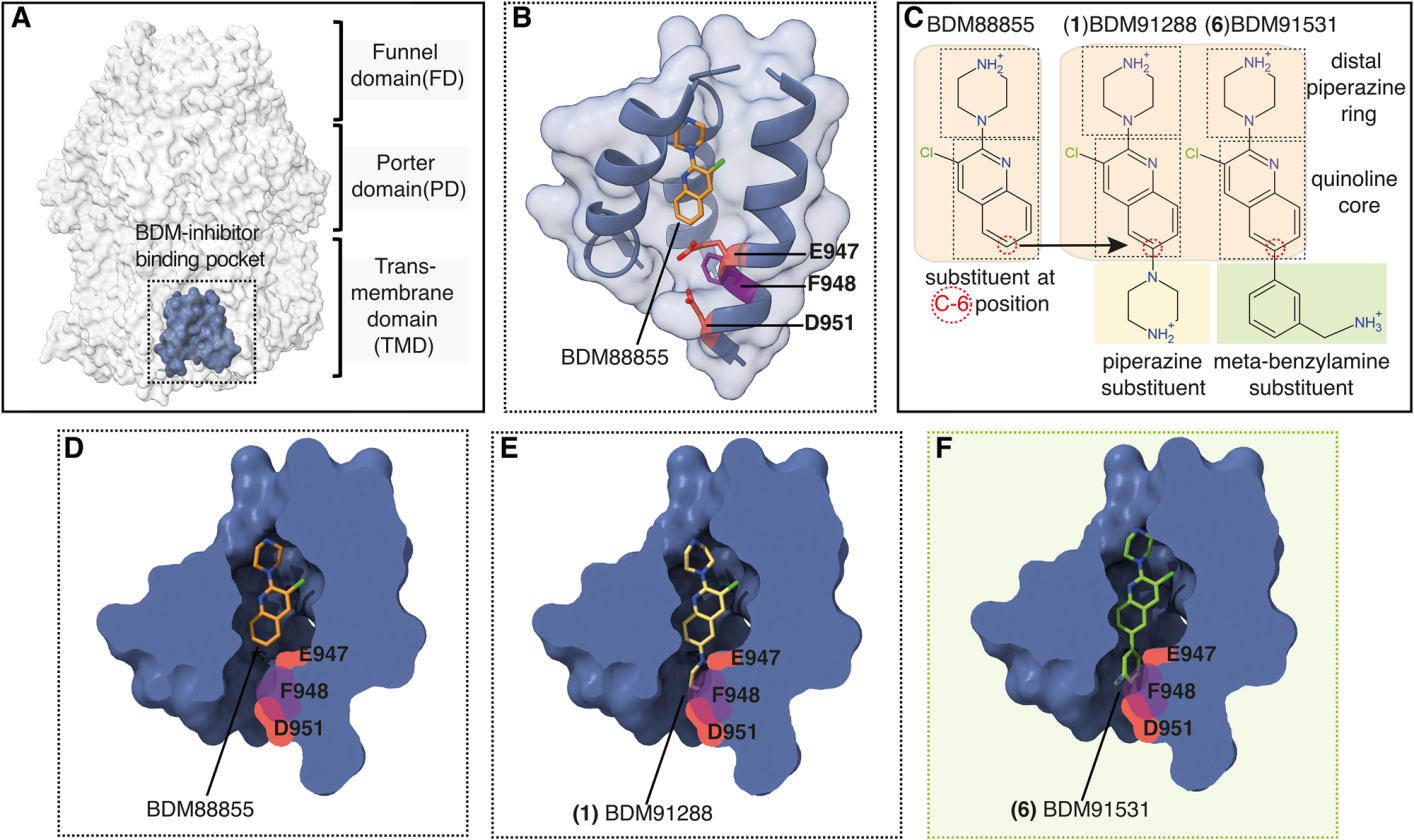
PyrPip-based inhibitors targeting interactions with the carboxylate groups of E947 and D951 within the AcrB inhibitor binding pocket. **A** AcrB can be structurally divided into three domains: transmembrane domain (TMD), porter domain (PD), and funnel domain (FD). The PyrPip-inhibitor binding pocket within the TMD of one AcrB protomer is depicted in blue. **B**, **D** The lead structure BDM88855 (orange) and the targeted residues F948 (violet), E947 and D951 (red) are shown within the *E. coli* AcrB binding pocket (PDB 7OUK). **C** Lead-optimization starting from BDM88855^19^ led to an improved BDM-series, including BDM91288 (**1**) with a piperazine substituent published in Vieira Da Cruz et al.^20^ and BDM91531 (**6**) in this study, with an extended meta-benzylamine substituent at the C-6 position. **E**, **F** The BDM91288 (**1**) and BDM91531 (**6**) molecules were placed into the AcrB inhibitor binding pocket congruent with the binding position of BDM88855 (PDB 7OUK)^19^. Potential interactions with the residues E947, D951 (red) and F948 (violet) were targeted by the structure-based drug design.

Antibiotic resistance is a significant concern in the healthcare sector, with infections caused by multi-drug resistant (MDR) Gram-negative bacteria posing a substantial threat^1^. A key resistance mechanism in these bacteria is the active expulsion of multiple antibiotics by efflux pumps from the Resistance Nodulation and cell Division (RND)-superfamily, undermining the effectiveness of antibiotics^2,3^. RND-type efflux pumps are complex tripartite structures consisting of an inner membrane (IM) transporter, a periplasmic adapter protein (PAP), and an outer membrane (OM) channel^4^. In *Escherichia coli* and *Klebsiella pneumoniae*, the prominent MDR efflux transporter is AcrAB-TolC^5,6^. The homotrimeric IM transporter AcrB determines substrate specificity for the entire tripartite complex and is energized by the proton motive force (pmf). Structurally, AcrB consists of three major regions: (i) a transmembrane domain (TMD) that mediates proton translocation between the periplasm and cytoplasm, (ii) a porter domain (PD) that recognizes and transports a wide range of antibiotics, bile salts, dyes, and detergents, and (iii) a funnel or docking domain (FD) that recruits the hexameric periplasmic adaptor protein AcrA (Fig. 1A). Hexameric AcrA engages the trimeric outer membrane channel TolC, and together AcrA and TolC form a continuous tunnel that expels substrates across the outer membrane (Fig. S1)^4^.

The protomers within the AcrB trimer interdependently cycle through loose (L), tight (T), or open (O) conformational states during drug/H^+^ antiport catalysis^7–9^. Drug substrates enter through specific channels in the periplasmic PD accessing either proximal access pocket (AP) in the L protomer or the more distal deep binding pocket (DBP) in the T protomer. Drug binding to the DBP in the T state triggers structural changes that enable proton transfer from the periplasm toward the titratable residues D407 and D408 within the TMD. Proton binding to these residues alters electrostatics in the TMD, resulting in a conformational change that is transduced to the PD, leading to the O state. The T to O transition results in the closure of all entry channels and the opening of an exit channel towards the PAP/OM channel. The O protomer, where the D407 and D408 residues in the TMD are protonated, is subsequently regenerated to the L state upon pmf-dependent H^+^-release toward the cytoplasm^4^. The opening and closing of the channels within the AcrB PD are postulated to occur in a peristaltic fashion, forcing the direction of drug transport^8^. An intermediate peristaltic conformation between the L and T states including a trapped drug, has recently been shown^10^.

The inhibition of RND-type efflux pumps has been a long-time goal to increase bacterial sensitivity to common antibiotics^11^. Various classes of efflux pump inhibitors (EPIs) have been reported that act at the DBP on AcrB, such as PAβN, NMP, D13-9001, MBX or EMP compounds^12–17^. Thus, EPI drug discovery has foremost been targeting the highly hydrophobic polyspecific DBP of RND efflux pumps. The need for substantial hydrophobic interactions has led to either poorly soluble or amphiphilic EPIs with unfavourable pharmacokinetic and toxicological profiles reaching clinical stages. Additionally, successful EPI development has faced significant challenges in achieving selectivity, due to a limited understanding of binding pocket interactions^18^.

Recently, a new class of EPIs based on a pyridylpiperazine (PyrPip) scaffold has been shown to bind to a previously undiscovered allosteric pocket in the AcrB TMD. In this PyrPip series, BDM88855 displays micromolar affinity and likely inhibits drug efflux by allosterically preventing the functional cycling of AcrB protomers (Fig. 1B–D)^19^. In addition, another PyrPip EPI, BDM91288, with comparable affinity but improved physico-chemical and pharmacokinetic properties has also been reported^20^. BDM91288 restored in vitro antibiotic susceptibility in resistant *K. pneumoniae* strains that overexpress AcrAB-TolC and enhanced the in vivo efficacy of levofloxacin in a *K. pneumoniae* lung infection mouse model^20^.

Single-particle cryo-EM co-structure of *K. pneumoniae* AcrB bound to BDM91288 (PDB: 8P1I) revealed that the additional piperazine substituent introduced in BDM91288 interacts with the acidic side chain of D950 located at the cytoplasmic entrance of the L protomer^20^.

*E. coli* AcrB comprises two carboxylates, specifically E947 and the corresponding D951 (D950 in *K. pneumoniae* AcrB), as well as a lipophilic phenylalanine side chain (F948) in this cytoplasmic cavity (Fig. 1B) which present opportunities for the development of high-affinity PyrPip inhibitors (Fig. 1C, E, F) by targeting these features.

Here we present the structure-based medicinal chemistry (MedChem) optimization of the PyrPip scaffold resulting in the nanomolar potent inhibitor BDM91531. We resolved the co-structures of *E. coli* AcrB with bound BDM91531 via X-ray crystallography at 1.94 Å and single particle cryo-EM at 3.23 Å global resolution. Differential scanning fluorimetry (DSF) assays, and site-directed mutagenesis phenotype studies further elucidated the mode of action of this inhibitor. BDM91531 binds to the TMD of the L state and O state protomers of AcrB via a directional two-point suspension and requires negatively charged residues at the cytoplasmic rim for entry. These results pave the way for further improvement in developing high-affinity RND-type efflux pump inhibitors towards clinical applications.

## Results

### Structure-based design of enhanced affinity PyrPip-inhibitors

Efflux pump inhibitors uniquely restore the efficacy of antibiotics by reversing resistance mechanisms, making them powerful agents in adjuvant therapies. Such inhibitors should be effective at low concentrations, which requires high affinity for the target — a feature that can be achieved by enhancing ligand–target interactions. Thus, the primary aim of this study was the potency enhancement of PyrPip inhibitors BDM88855 (EC_90_ = 3.6 μM) and BDM91288 (EC_90_ = 2.6 μM)^19,20^(Table 1). To achieve this, we focused our structure-based design on gaining targeted interactions with carboxylates E947 and D951 located on the cytoplasmic entry of the AcrB inhibitor binding pocket (Fig. 1B). We synthesized new inhibitors with substituents containing a primary or secondary amine at the C-6 position of the BDM88855 quinoline core that faces the carboxylate residues of interest (Table 1, Fig. 1C). All compounds were tested in combination with a sub-active dose of pyridomycin, a model antibiotic substrate of AcrB, to determine their effective concentration (EC_90_). Initially, substitution of the quinoline ring with aminomethyl-piperidines (**2**, EC_90_ = 3.9 μM and **3**, EC_90_ = 5.2 μM) or -pyrrolidine (**4**, EC_90_ = 3.9 μM) yielded compounds with similar potency compared to BDM91288 (**1**, EC_90_ = 2.6 μM), which was previously used in our *K. pneumoniae* AcrB studies^20^ (Table 1).

**Table 1:** Biological activities of compounds 1-11


Compound	Structure	EPI antibiotic boosting activity EC_90_ (µM [range])^a^	EPI antibiotic activity MIC_90_ (µM)^b^	EC_90_ *E. coli* E947Q EC_90_ µM [range]^c^ (fold difference)	EC_90_ *E. coli* D951N EC_90_ µM [range]^c^ (fold difference)
**1 BDM91288**		2.6 [1.9–3.8]	> 250	2.6 [1.9–3.8]	62 [31– > 62] (24-fold)
**2**		3.9	> 250	13 [7.8–16] (3-fold)	62 [31– > 62] (16-fold)
**3**		5.2 [3.9–7.8]	> 250	6.5 [3.9–7.8]	41 [31–62] (8-fold)
**4**		3.9	250	6.5 [3.9–7.8]	62 [31– > 62] (16-fold)
**5**		0.48	250	7.8 (16-fold)	7.8 (16-fold)
**6 BDM91531**		0.24	62	1.9 (8-fold)	5.2 [3.9–7.8] (16-fold)
**7**		1.6 [0.97–1.9]	62	1.6 [0.97–1.9]	13 [7.8–16] (8-fold)
**8**		5.2 [3.9–7.8]	125	2.6[1.9–3.9]	13 [7.8–16] (3-fold)
**9**		5.2 [3.9–7.8]	125	2.6 [1.9–3.9]	10 [7.8–16]
**10**		0.97	31	3.2 [1.9–3.9] (3-fold)	3.2 [1.9–3.9] (3-fold)
**11**		0.81 [0.48–0.97]	62	1.3 [0.97–1.9]	6.5 [3.9–7.8] (8-fold)

^a^EC_90_ represents the 90% maximal effective concentration of tested compounds that prevents the growth of *E. coli* BW25113 in the presence of 5 μg/mL pyridomycin as measured by resazurin reduction (the MIC_90_ of pyridomycin alone is 12.5–25 μg/mL). Data are the result of at least three biological replicates and are presented as mean and range of values. ^b^MIC_90_ represents the minimal inhibitory concentration of tested compounds that prevent 90% of *E. coli* BW25113 growth as measured by resazurin reduction. ^c^EC_90_ represents the 90% maximal effective concentration of tested compounds that prevents the growth of *E. coli* BW25113 AcrB-E947Q or AcrB-D951N in the presence of 5 μg/mL pyridomycin as measured by resazurin reduction. All data are the result of three biological replicates and are presented as mean and range of values.

As the co-crystal structure of BDM88855 with AcrB (pdb 7OUK)^19^ indicates the presence of a phenylalanine residue (F948) in proximity to the quinoline ring (Fig. 1D), we designed compounds bearing diverse substituted phenyl rings to potentially interact with F948. The introduction of ortho- (**5**, EC_90_ = 0.48 μM), meta- (**6**, EC_90_ = 0.24 μM), and para- (**7**, EC_90_ = 1.6 μM) benzylamine substituents at position 6 of the quinoline ring led to a 2- to 10-fold improvement in potency compared to BDM91288 (Table 1). Chain length elongation by the addition of a methylene group in meta or para position (compounds **10**, EC_90_ = 0.97 μM and **11**, EC_90_ = 0.81 μM) was tolerated, whereas replacing the benzylamine (**6**) with an aniline (**8**, EC_90_ = 5.2 μM) or introducing an alcohol (**9**, EC_90_ = 5.2 μM) in place of the primary amine moiety led to a decrease in potency. This suggests that the basic amine of compound **6** (BDM91531) is essential for a potential interaction with E947 or D951.

BDM91531 (**6**), which exhibited the greatest potentiation of pyridomycin activity (Table 1), was subsequently evaluated on the activity of a panel of diverse antibiotics to confirm that potentiation was due to the global inactivation of AcrB rather than antibiotic-specific effects. Data from this study confirmed that BDM91531 (**6**) exhibited broad spectrum potentiation of all antibiotics known to be substrates of AcrB on wildtype *E. coli* but not of the isogenic strain lacking *acrAB* (Table S1). Next, to further explore the dose dependent activity of BDM91531, checkboard combination studies were performed between BDM91531, and pyridomycin or oxacillin in wildtype and the AcrAB deficient mutant of *E. coli*. These checkerboard assays showed BDM91531 to mediate a dose dependent potentiation of both pyridomycin and oxacillin antibiotic activity in wildtype *E. coli* (Fig. S2)*¸* while this was not seen in *E. coli* lacking AcrAB, except at higher concentrations ( > 32 µM) where BDM91531 started to exhibit intrinsic antibacterial activity.

As such, all EPIs were also tested alone to determine their MICs and compounds **5**-**11** bearing a phenyl ring showed intrinsic antibacterial activity at high concentrations. The most potent inhibitors with nanomolar EC_90_ (**5,** **6,** **10** and **11**) displayed MICs in the micromolar range (MIC = 31–250 µM), thus, these compounds do not show antibacterial activity at concentrations used for effectively inhibiting AcrB. Overall, the structure-activity relationships underscore the significance of introducing aminomethyl- and aminoethyl-substituted phenyls on the quinoline ring for improving EPI potency. To confirm the interaction of the amino group of the inhibitors with the targeted AcrB acidic residues E947 or D951, all compounds were tested in combination with pyridomycin in a susceptibility assay on *E. coli* strains harbouring the AcrB E947Q and D951N substitutions (Table 1). These introduced substitutions by genetic mutation did not impair the functional activity of AcrB, as mutant strains exhibited susceptibility to pyridomycin comparable to that of the wild-type strain. While the E947Q AcrB substitution decreased the activity of a couple of PyrPips (**2,** **5,** **6**, and **10**), the D951N substitution had an equal or greater impact on PyrPip activity (Table 1). These data support the involvement of the two acidic residues in forming the intended ionic interactions with the introduced basic amino group on the PyrPip EPI and/or suggest that the carboxylates facilitate the entry of inhibitors into the AcrB binding pocket.

To evaluate the involvement of E947 and D951 in inhibitor binding and entry, we conducted structural studies with the most potent compound BDM91531 (**6**) using X-ray and cryo-EM analyses and estimated binding affinity using DSF assays with purified AcrB and AcrB substitution variants. Growth curve analysis of *E. coli* strains harbouring AcrB variants was conducted to investigate the role of carboxylate side chains in facilitating inhibitor binding.

### Structural analysis of nanomolar affinity binding of BDM91531

**Fig. 2:**
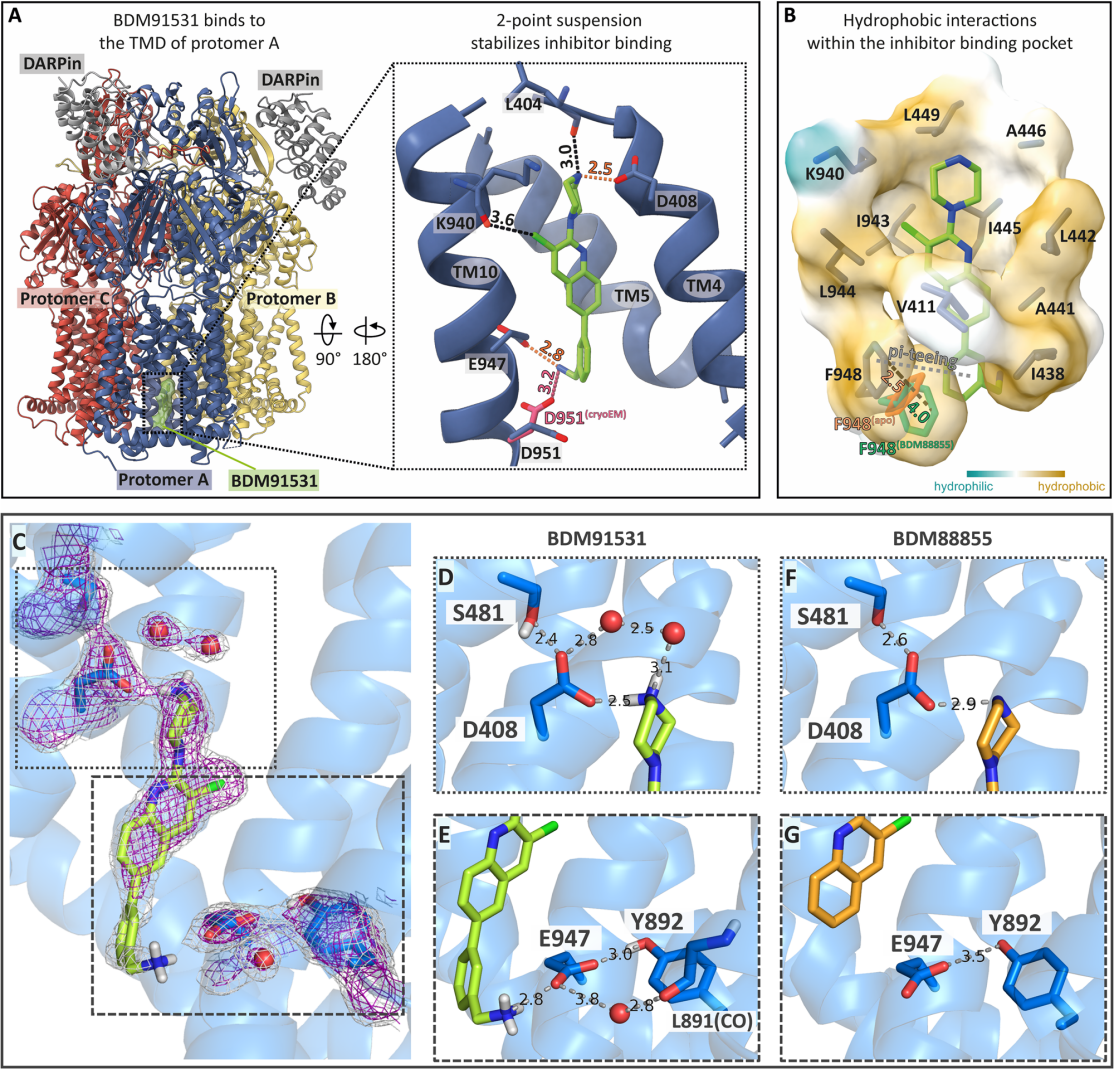
Binding site suspension points of the PyrPip-based efflux pump inhibitor BDM91531 in complex with EcAcrB (X-ray structure). **A** Side view of the AcrB trimer comprising protomers A-C (blue, yellow, red, respectively). The inhibitor binds centrally within the TMD of protomer A, located between TM4, 5 and 10. Inset: The inhibitor binding pocket (side view) illustrates the ionic and polar interactions between the BDM-inhibitor and the residues of TM4, 5 and 10. The crucial interactions with the D408 and E947 carboxylates, forming a 2-point suspension, are indicated in red lines. Other polar interactions are shown in black. D951 reveals an alternative orientation in the cryo-EM co-structure EcAcrB with BDM91531 (pink) forming a possible ionic interaction with the secondary amine of the benzylamine moiety. Distances are indicated in Å at the dashed lines. **B** The hydrophobicity map highlights the predominant hydrophobic interactions within the inhibitor binding pocket, with amino acid hydrophobicity color-coded according to the Kyte-Doolittle scale. At the entrance of the binding pocket, the F948 side chain in the AcrB/BDM91531 co-structure is displaced by 2.5 Å from its apo position (orange, PDB: 4DX5) and by 4.0 Å from its position in the BDM88855-bound structure (dark green, PDB: 7OUK). The potential π-teeing interaction between the phenyl ring of F948 and the benzylamine moiety of BDM91531 is indicated. **C** Side view on the AcrB/BDM91531 binding pocket with major ionic and polar interactions between BDM91531 and side chains D408, S481, E947, Y892 and L891. In addition to these residues, three coordinated water molecules (shown as red spheres) contribute to inhibitor binding. Structure and electron densities (2Fo-Fc at 0.9 σ (gray) and 1.5 σ (purple)) shown are from the 1.94 Å resolution co-crystal structure. Densities are continuous between S481, D408 and the secondary amine of BDM91531 indicating shared electrons. The upper boxed region (dotted lines) indicates the area shown in (**B**, **D**) and the lower boxed region (dashed lines) indicate the area shown in (**C**, **E**). **D** The upper suspension point is defined by the short hydrogen bond distance between D408 and S481 (2.4 Å) and a salt bridge (2.5 Å) between D408 and the secondary amine of BDM91531. **E** The lower suspension point is characterized by a salt bridge between the primary amino group of BDM91531 and the E947 carboxylate. The interaction is supported by an extended network of hydrogen bonds including Y892, a water molecule, and the L891 backbone carbonyl oxygen. **F** Interactions of D408 and S481 with BDM88855 (PDB 7OUK) appear similar yet with longer distances. The AcrB/BDM88855 crystal co-structure was, however, less well resolved (2.7 Å)^19^. **G** There is no interaction between BDM88855 and E947.

An AcrB/DARPin/BDM91531 (**6**) co-crystal X-ray structure was solved at 1.94 Å resolution (Table S2). We designated the three protomers in the structure as A, B, and C, where in the apo (non-inhibited) trimer structure (pdb: 2GIF) these protomers adopt the L, T and O states, respectively^8^. BDM91531 (**6**) binds to the TMD of the AcrB protomer A as previously described for the AcrB/DARPin/BDM88855 co-structure (pdb: 7OUK)^19^ (Fig. 2A).

The elongated transmembrane inhibitor binding pocket includes charged amino acid residues which are critical for inhibitor binding and act as hydrogen bonding donors and acceptors (Fig. 2A). BDM91531 binding is further dominantly supported by comprehensive hydrophobic interactions (Fig. 2B).

**Fig. 3:**
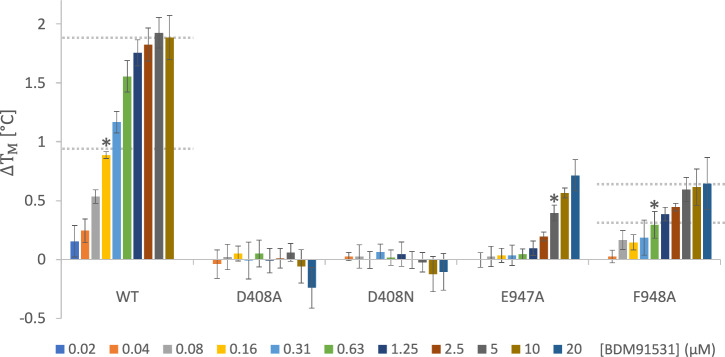
Differential scanning fluorimetry reveals thermal shifts in protein melting temperature in dependence on BDM91531 concentration. Solubilized AcrB wildtype or substitution variants E947A, D408A, D408N and F948A were incubated in presence of 0–20 µM (for wildtype AcrB: 0–10 µM) BDM91531 (**6**) during cysteine-reactive fluorophore CPM-based melting temperature determination. Thermal shifts (ΔT_M_) from five DSF measurements (two different protein purifications) in presence of BDM91531 are shown as deviations from the respective apo protein melting temperature. The average values are shown in the bar chart with standard deviation. Maximum and half-maximum ΔT_M_ values were represented by dashed lines. Wildtype AcrB showed a concentration dependent saturation with a maximum stabilization of 1.89 ± 0.19 °C and a half-maximal effect (*) at approx. 0.16 µM. The inactive variants D408A and D408N were not thermal stabilized in presence of the inhibitor indicating lack of binding. Thermal stabilization values in presence of inhibitor obtained with protein variants F948A and E947A were less pronounced, but clearly visible with half maximal effects (*) around 0.63 µM (F948A) and ≥ 5 µM (E947A). While thermal stabilization by BDM91531 of the F948A variant appears to converge at 0.62 ± 0.15 °C above the apo F948A T_M_ value, saturation could not be achieved for the E947A variant within the technically accessible concentration range of 0.04–20 µM. CPM: 7-diethylamino-3-(4-maleimidophenyl)-4-methylcoumarin.

Under physiological conditions, the secondary amine of the distal piperazine ring (pK_a_ = 9.7) and the primary amine of the proximal benzylamine group (pK_a_ = 9.3) are both positively charged (Fig.1C). This protonation allows for strong ionic interactions with the negatively charged D408 (distance 2.5 Å) and E947 (distance 2.8 Å) carboxylates (Fig. 2A). The presence of a clear and continuous electron density between the secondary piperazine amine with one of the D408 carboxylate oxygens indicates a strong salt bridge (Fig. 2c) and suggests sharing of electrons and the proton (H^+^). DSF data shows that the AcrB D408A and D408N substitution variants are completely devoid of BDM91531 binding (Fig. 3, Table S3). Both the proximity between the secondary piperazine amine of BDM91531 and the D408 carboxylate coupled with the hydrogen-bond interaction with the carbonyl oxygen of the L404 main chain (Fig. 2A–D) suggests that this ionic interaction is crucial for BDM91531 binding. The absence of BDM91531 binding to the wildtype conformation-equivalent D408N variant^21^ supports this notion. In addition, the introduced benzylamine-substituent of BDM91531 interacts with the carboxylate side chain of E947, which is located at the cytoplasmic rim region of AcrB (Figs. 1F, 2A). BDM91531 binding is therefore stabilized by two defined suspension points (Fig. 2A). The highly conserved suspension points D408 and E947 are supported via hydrogen bond interaction with residues S481 and between Y892, respectively (Fig. 2D, E). Each carboxylate is additionally stabilized by hydrogen bonding via a nearby structural water molecule (Fig. 2D, E). Whereas the crucial interaction between the secondary piperazine amine of the µM affinity inhibitor BDM88855 and the functionally essential D408 carboxylate was also present in the AcrB/BDM88855 co-structure^19^, the additional stabilizing interaction with E947 was absent since this inhibitor does not carry a second basic amino group (Fig. 1B, D, Fig. 2F, G). Instead, the target amino group of BDM91531 interacts exclusively with E947 (Fig. 2A, E), and the carboxylate group of D951 is located at a distance of 4.6 Å in the X-ray structure.

Predominant hydrophobic interactions are observed between the BDM91531 PyrPip-core and the nearby ( < 4 Å) conserved residues V411, I438, I445, and L944 of the inhibitor binding pocket (Fig. 2B). Other hydrophobic side chains involved in BDM91531 interaction include A441, L442, A446, L449, I943 as well as the alkyl moiety of K940 (Fig. 2B), whose primary amino group forms an ionic interaction with nearby D407, and its carbonyl oxygen forms a crucial halogen bond with the chlorine atom on BDM91531 (Fig. 2A)^19^. E947 (C_γ_) and F948, both interacting with the benzylamine ring of BDM91531, appear to further contribute to the predominantly hydrophobic character of the inhibitor binding site. The benzylamine extension of BDM91531 displaces the F948 side chain from its apo-position (pdb: 4DX5)^22^ by 2.5 Å and from the previously determined BDM88855 bound co-structure (pdb: 7OUK) by 4.0 Å^19^. (Fig. 2B). The resulting orthogonal orientation between the F948 phenyl ring and the BDM91531 benzylamine ring might form a T-shaped π-π interaction (π-teeing) (Fig. 2B). In addition, F948 appears to remain in a cation-π-interaction with the guanidinium group of R971 as observed for the apo O state^7–9^.

### D408 and E947 as key determinants for nanomolar binding affinity

Structural analysis suggests that BDM91531 exhibits stronger binding to AcrB (Fig. 1F) compared to BDM88855 (Fig. 1D) attributed to additional interactions with residues E947 and F948. To investigate the contributions of these side chains to BDM91531 binding, DSF assays were done using purified AcrB wildtype and substitution variants E947A, F948A, as well as the control variants D408A and D408N. A BDM91531 titration series ranging from 0 to 20 µM was applied. In the presence of BDM91531, the melting temperature (T_M_) of purified wildtype AcrB increased up to 1.89 ± 0.19 °C (Fig.3, Table S3), indicating compound-induced thermal stabilization. We observed half-maximal thermal stabilization of AcrB at a BDM91531 concentration of approximately 156 nM. However, due to technical constraints limiting our ability to measure concentrations lower than 150 nM AcrB trimers, we anticipate that the affinity between AcrB and BDM91531 is likely higher. In context, when compared to the previously studied AcrB inhibitor BDM88855, which exhibited a half-maximal effect at concentrations between 5 and 10 µM^19^, the PyrPip optimization to BDM91531 through rational MedChem design has resulted in an estimated 50-fold increase in affinity.

When AcrB D408 was substituted with Ala or Asn, increasing concentrations of BDM91531 did not lead to a change in protein melting temperature, indicating that the PyrPip did not bind to these AcrB variants (Fig. 3, Table S3). Since the AcrB variant D408N was solved in a wildtype structural conformation (Eicher et al. 2014) and similar T_M_ values for D408N AcrB (54.23 ± 0.10°C) and wildtype AcrB (54.00 ± 0.12°C) without the presence of the inhibitor are observed (Fig. 3, Table S3), an interaction between the inhibitor and D408 solely based on hydrogen bonding appears unlikely. Moreover, equivalent results were obtained for the D408A variant with BDM88855 (Fig. S3), which appears not to bind. The lack of inhibition by BDM88855 of cells harbouring the marginal active D408N AcrB variant^23^ when exposed to erythromycin (Fig. S4) further supports the lack of binding in the absence of a carboxylate at position 408. When these cells were exposed to the deep binding pocket inhibitor MBX3132 in combination with erythromycin, no growth occurred. This confirms the prime interaction between the secondary piperazine amine of the PyrPips and the D408 carboxylate. We therefore propose an electrostatic interaction between the charged carboxylate side chain and the secondary piperazine amine of BDM88855 or BDM91531.

The E947A substitution (apo T_M_ 54.01 ± 0.09 °C, comparable to wildtype AcrB apo T_M_) resulted in a significant reduction in the apparent BDM91531 affinity to ≥ 5 µM (Fig. 3, Table S3), correlating with the increase in EC_90_ of *E. coli* harbouring the E947Q variant (Table 1). For this variant, BDM91531 saturation could not be achieved, as concentrations higher than 20 μM led to protein destabilization (Fig. 3, Table S3), thereby interfering with the half-maximal stabilizing concentration estimation. These data agree with the impact of E947 mutations on the boosting of pyridomycin activity by BDM91531.

As previously reported^19^, when compared to the wildtype the substitution of F948 to Ala caused inhibitor hypersensitivity and leads to decreased cell growth in the presence of drugs and BDM88855. This suggests that F948, situated at the cytoplasmic rim of the AcrB TMD, might sterically interfere with BDM88855 entry. Conversely, substituting F948 with a sterically smaller Ala, facilitated inhibitor entry. In the wildtype AcrB/BDM91531 co-structure, the interaction between BDM91531 and F948 supports a positive contribution by the observed π-teeing (Fig. 2b). In contrast to the proposed steric interference which decreases the affinity of BDM88855, F948 rather appears to positively contribute to BDM91531 affinity.

Indeed, when the AcrB F948A variant was evaluated by DSF in presence of BDM91531, the half maximal thermal stabilization was increased 4-fold reaching approx. 625 nM. This suggests a decrease in its affinity when the phenyl ring is removed and appears to contribute to BDM91531 affinity through F948 in wildtype AcrB (Fig. 3, Table S3).

### Transition-state inhibitor stabilization of AcrB hinders functional rotation

The nanomolar binding of BDM91531 to AcrB occurs to one of the protomers (protomer A) as determined by the X-ray co-structure (Fig. 2a) and leads to effective inhibition of efflux activity (Table 1). In the AcrB/DARPin/BDM91531 co-crystal X-ray structure, protomers B and C adopt the known conformational states tight (T), open (O) (Table S4), whereas the PD of protomer A adopts the L state. The TMD of this protomer A features an O to L state characteristic (Fig. S5b, Table S4).

**Fig. 4:**
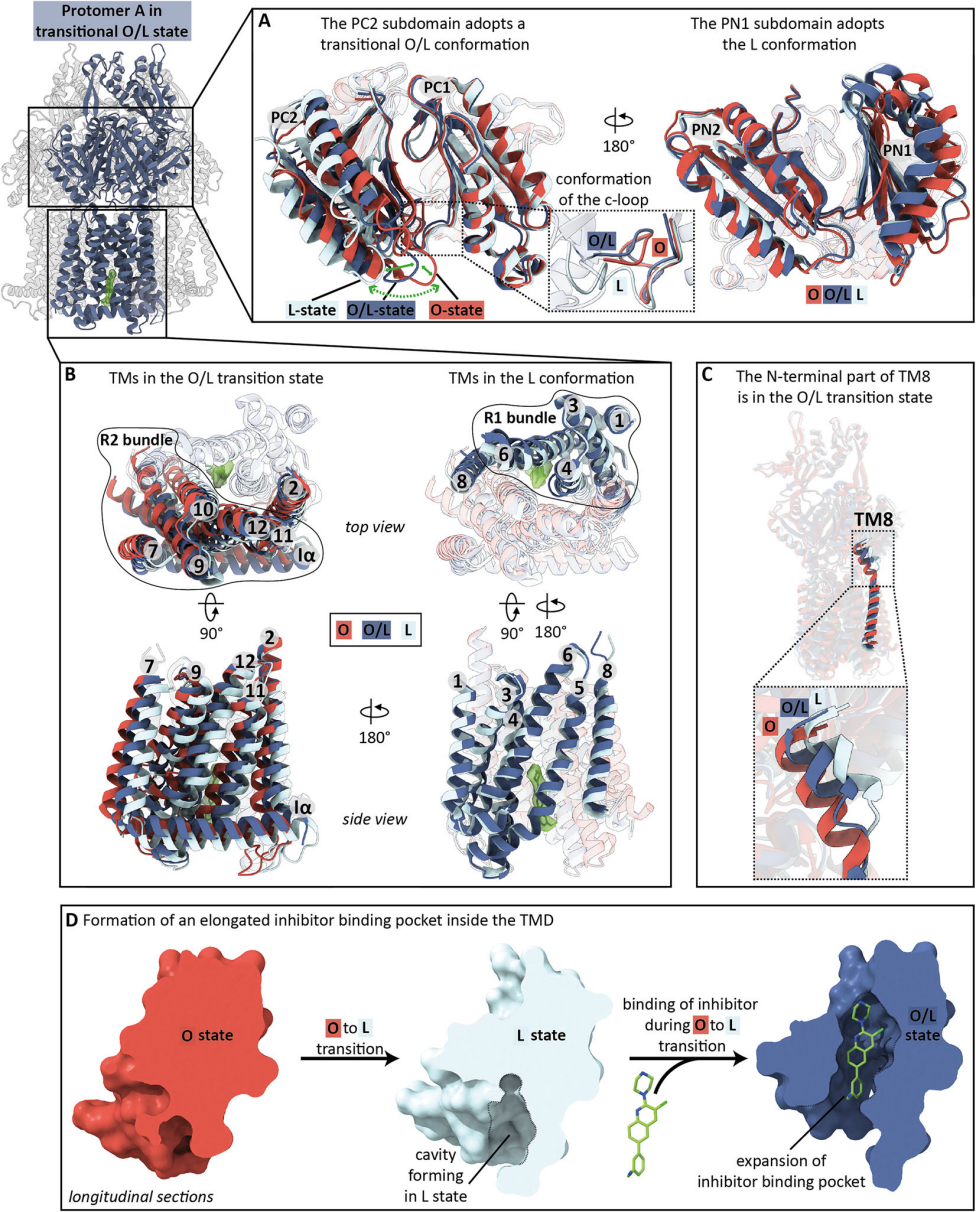
BDM-inhibitor binding induces a trapped transitional O/L conformational state in protomer A, hindering the functional rotation of AcrB (cryo-EM structure). **A** The PC2 subdomain adopts a trapped transitional O/L conformation (dark blue, cryo-EM class I co-structure from this study), while the c-loop remains in a folded conformation similar to the O state (red, PDB: 4DX5), and the PN1 subdomain is in the L conformation (light blue, X-ray co-structure from this study). **B** The O/L trapped transition state (dark blue) is shown only for the cryo-EM class I co-structure, as the conformational O/L characteristics are congruent to the X-ray co-structure (C_α_ RMSD: 0.49, Table S4). Within the TMD, specifically the TMHs of the R2 bundle (7, 9, 10, 11, 7, 9, 10, 11, 12 (from Gly1004)^21^, TMH2 and helix Iα adopt the O/L trapped transition state (dark blue) (top and side view). The TMHs of the R1 bundle (1, 3, 4, 5, 6) and TMH8 (up to Gln872) adopt the L conformation (light blue) (top and back side view). The O (red) and L (light blue) states are shown as references from the apo AcrB structure without bound inhibitor (PDB: 4DX5). **C** The N-terminal part of TMH8 adopts the O/L trapped transition state (dark blue, cryo-EM class I). **D** Longitudinal sections of the cavity forming in the L state (light blue) upon O (red) to L transition and the elongated inhibitor binding pocket in the O/L state (dark blue). The cavity formed for proton-release during the O to L transition is suggested to expand upon BDM-inhibitor binding.

The crystallization of the AcrB/BDM91531 X-ray co-structure was facilitated by DARPins, which recognize the porter domain of AcrB^9^. Because the PD and TMD are conformationally coupled, it is possible that DARPin binding constraints additional conformational impact of BDM91531 binding. We therefore subjected dodecylmaltoside (DDM) solubilized AcrB in the presence of BDM91531 in absence of DARPins to single particle cryo-EM analysis (Fig. 4, Fig. S5c, d, Table S5, Table S6, Fig. S10). We obtained two different classes of 3D maps (Fig. S6). The cryo-EM map of the class I AcrB trimer (3.23 Å, C1 symmetry, Fig. S6) revealed that BDM91531 is bound to protomer A, similar to the AcrB/DARPin/BDM91531 X-ray crystal structure (Fig. 2, Fig. 4, Fig. S5b, c, Table S4). The protomers B and C also adopt distinct conformational states: tight (T) and open (O), respectively (Table S4, Fig. S5b, c). In accordance, the TMD of protomer A in complex with BDM91531 exhibits the same O/L conformational state as observed in the X-ray structure, with an all-atom root mean square deviation (RMSD) of 0.49 Å between the individual TMDs (Fig. S5b, c, Table S4). In contrast to the DARPin-bound X-ray co-crystal structure, whose protomer A periplasmic domain is in the apo L state, the entire protomer A of the cryo-EM structure adopts an O/L trapped transitional state (Fig. 4A, Fig. S5c, Table S4). Specifically, the periplasmic PC2 subdomain and N-terminal part of TM8 remain in an O/L trapped transition state (Fig. 4A, C). The c-loop (Asn667 – Gly679), however, remains in a similar folded conformation as found within the O state of AcrB (PDB: 4DX5)^22^, whereas the PN1 subdomain already adopted the L conformation (Fig. 4A). These observations suggest that within the X-ray structure the PD of the inhibitor bound protomer A is shifted towards the L conformation upon DARPin binding and/or crystal packing.

Interestingly, the cryo-EM O/L PD intermediate of the BDM91531-bound protomer A shows remarkable similarity to the X-ray structure of a double Cys-substituted cross-linked AcrB variant (AcrB_S562C_T837C/DARPin, Table S2, Fig. S5a, Fig. S7), which was previously reported to cause efflux deficiency for all tested drugs^24^. In the latter structure, a crosslink is apparent in protomer A between TM7 (T837C) and the PC2 (S562C) subdomain, causing the periplasmic PC2 subdomain to remain in an O/L trapped transition (Fig. S7). The TMD of the cross-linked protomer A adopts the L conformation, except for the tilted, almost O state-like conformation of the N-terminal part of TM8 (Fig. S7b). The non-cross-linked protomers B and C adopt the T and O states, respectively. Comparison of RMSDs between BDM91531-bound and crosslinked AcrB protomers (Table S4) suggests that both BDM91531 binding and Cys-crosslinking trap the PC2 subdomain in a similar transitional conformation during the functional cycle of AcrB. Given that disulfide cross-link formation most likely occurs during catalysis^24^, the structural resemblance to the BDM91531-bound state supports the conclusion that BDM91531 acts as a transition state inhibitor (Fig. 4D).

### BDM-inhibitor binding to two protomers within the AcrB trimer

Next to the cryo-EM class I particles described above (Fig. 4, Fig. S6), the DDM-solubilized AcrB/BDM91531 sample also resulted in a class II cryo-EM map (3.52 Å, C1 symmetry, Fig. S6, Fig. S10). The electron density maps from this class revealed an AcrB trimer with two molecules of BDM91531 bound, one to protomer A and one to protomer C (Fig. S5d, Fig. S6, Fig. S8). In this class II, protomer B adopts the T state with no inhibitor bound (Table S4, Fig S5d). Protomer A adopts the O to L state in the TMD region congruent to the O to L trapped state described for cryo-EM class I and the X-ray structures with bound BDM91531 (Fig. S5c, d, Fig. S8a, Table S4). The PD of protomer A is in the L state (Fig. S8b, Table S3) as observed in the X-ray structure (Fig. S5b). This class II PD conformation is therefore different from the conformation of the PD of class I, which is present in the O/L trapped transition state (Fig. 4A, Fig. S5c, d). The BDM91531-bound protomer C mainly adopts the O conformational state (i.e., TMs 2, 3, 6, 7, 12), while TMs 1, 4, 5, 8-11 are found in a trapped transitional O/L conformation to adopt the inhibitor (Fig. S8b, Table S4). Furthermore, the PD in this protomer remains in the O state (Table S4, Fig. S8b).

We previously solved a structure of *K. pneumoniae* AcrB in complex with BDM91288 that was congruent to class I *E. coli* AcrB/BDM91531 co-structure (Fig. S5e, Table S4)^20^. Reanalysis of the cryo-EM particle data identified densities which corresponded to the *K. pneumoniae* AcrB in complex with two molecules of BDM91288 (Fig. S5f, Fig. S9). This *K. pneumoniae* AcrB/BDM91288 class II co-structure at 3.42 Å (C1-symmetry, Fig. S9, Fig. S10) was congruent to the *E. coli* AcrB/BDM91531 class II co-structure (Fig. S5d, f, Fig S8b, Table S4). We therefore find for both AcrB efflux pumps from *K. pneumoniae* and *E. coli* that the PyrPip inhibitors bind to the trimer either to protomer A only, or to protomer A and protomer C concurrently. For the *K. pneumoniae* AcrB trimer with one PyrPip inhibitor bound, the binding site to the L protomer induces a O to L intermediate state in the TMD. This intermediate trapped transition state is transduced to the PD, in specific the PC2 subdomain, which adopts an O to L trapped transition state as well. The rest of the PD (subdomains PN1, PN2, and PC1) is congruent to the apo-L state.

About one third of the particles within a purified AcrB sample were designated as class II particles with two BDM91531 or BDM91288 molecules bound to protomers A and C of both the *E. coli* and *K. pneumoniae* AcrB trimer, respectively (Fig. S8, Fig. S9, Fig. S10). Since class I particles with one inhibitor bound per trimer in the *E. coli* AcrB/BDM91531 samples exceeded the number of class II particles with two inhibitors bound to the trimer, we assume that high affinity ( ~ 150 nM) protomer A binding and a low affinity ( ~ 150 µM) protomer C binding occur in a concentration dependent manner. The low affinity of protomer C, which is apparently in the order of magnitude of the inhibitor concentration in the cryo-EM setup, is likely due to conformational constraints associated with the high affinity (first) binding event and the less accessible O conformation (Fig. 4D). As a result, the second binding event is probably not relevant for practical application.

### Inhibitor entry depends on charged environment at the cytoplasmic rim of AcrB

**Fig. 5:**
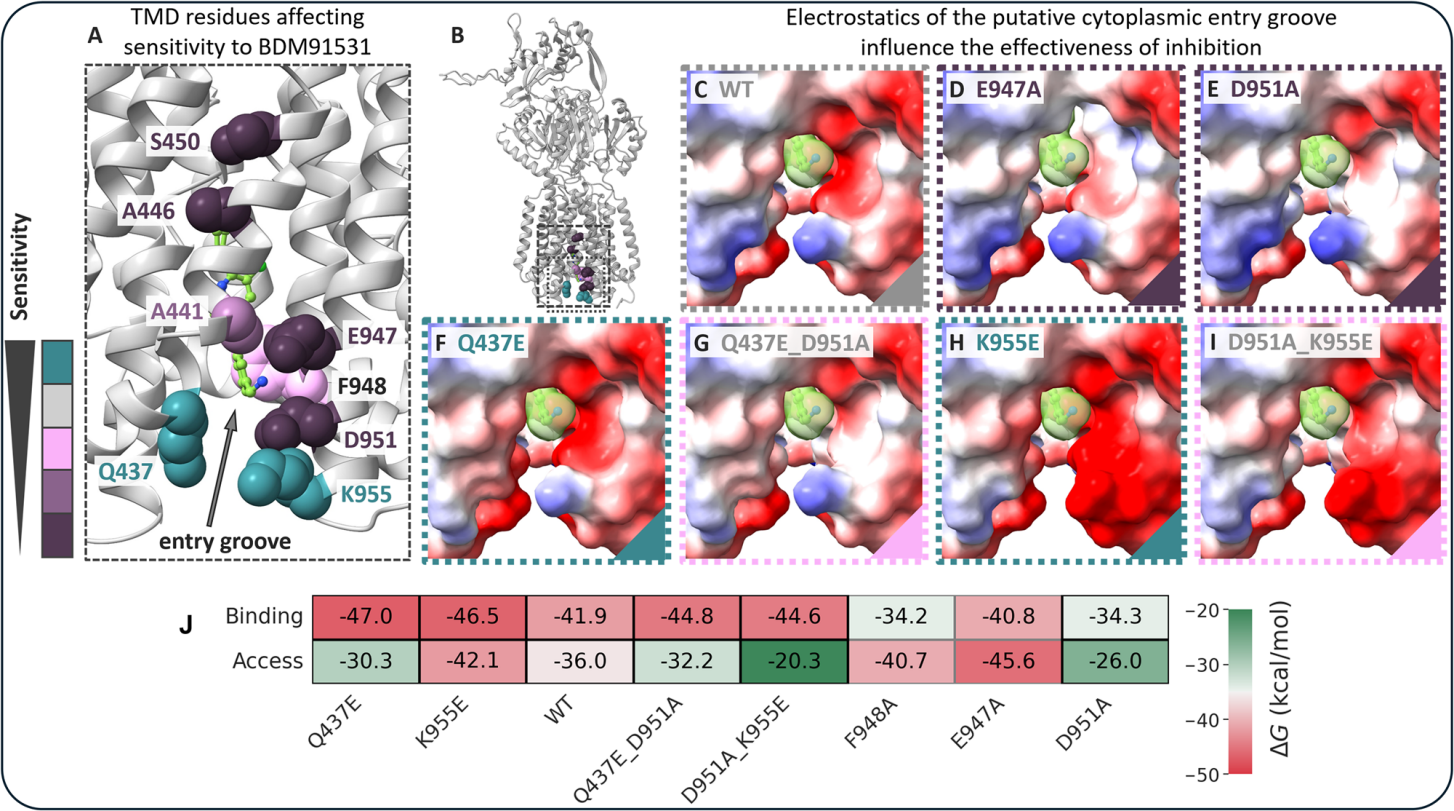
Electrostatic representation of the cytoplasmic inhibitor entry groove of AcrB wildtype and variants. Shown is a graphical interpretation of the phenotypical data obtained from the growth curve assay shown in Fig. S11. The results were obtained using an *E. coli* BW25113*ΔacrB*/pET24a_*acrB* expression system in the presence of 16 µg/ml erythromycin and 0 - 5 µM BDM91531. **A** Localization of the positions of the substitutions present in the tested AcrB variants in sphere representation. Color coding corresponds to the sensitivity towards the inhibitor. The WT (grey) is considered highly sensitive to BDM91531. Cytoplasmic rim substitution variants Q437E and K955E (teal) were even slightly more inhibitor sensitive than wildtype AcrB. Variants with intermediate sensitivity toward BDM91531 (F948A/I and A441L) and the insensitive variants A446P, S450P, E947A/Q or D951A/N are shown in light to dark purple. **B** Side view of the inhibitor-bound AcrB protomer indicating the cytoplasmic inhibitor entry groove region with the substituted side chains indicated in spheres. **C–I** structural models of substitution variants in the putative cytoplasmic inhibitor entry groove were created in PyMOL^38^ and subjected to electrostatic surface representation in ChimeraX^39^. **C** The entry groove of the WT is composed of mainly electronegative (red) regions but also includes electropositive (blue) surface areas. While the inhibitor binding pocket of the BDM91531 insensitive variants E947A (**D**) and D951A (**E**) are mainly determined by basic and apolar (white) areas, the BDM91531 hypersensitive variants Q437E (**F**) and K955E (**H**) have a predominantly electronegative character that presumably facilitates inhibitor entry. The double substitution variants Q437E_D951A (**G**) and D951A_K955E (**I**) in turn lead to an apparent wildtype-like electrostatic appearance, which is reflected by wildtype sensitivity as shown in Fig. S11. **J** Comparison of binding and accessibility of BDM91531 in AcrB mutants, where rows represent ΔG_binding_ (binding) and ΔG_Access_ (cytoplasmic entrance gate accessibility) in kcal/mol, and columns correspond to the different AcrB variants. The binding poses corresponding to each state is shown in Fig. S13 and Fig. S16.

The potency of the PyrPip inhibitor not only depends on its final binding position inside the TMD inhibitor binding pocket but might also rely on the accessibility of the pocket via the cytoplasmic TMD rim. We observed that residue E947 (Fig. 2A, Table 1) at the cytoplasmic rim is involved in inhibitor binding. BDM91531 is a divalent cation due to the presence of a primary and secondary amino group. Therefore, negatively charged side chains in and near the inhibitor binding pocket might facilitate the entry of the inhibitor molecule from the cytoplasm (Fig. 5). We tested the role of E947 and D951 by assessing the concentration-dependent inhibitory effect of BDM91531 on the activity of AcrB variants within the AcrAB-TolC efflux pump via growth curve analysis. For this purpose, the growth of *E. coli* expressing *acrB* wildtype or the different *acrB* mutants was examined over time in the presence of 16 µg/ml erythromycin (ERY) as a representative substrate of the AcrB pump in combination with a sub-MIC concentration series (0–5 µM) of BDM91531 (Fig. S11). The expression levels of all tested mutants were comparable to that of the wildtype *acrB* gene, as evidenced by Western blot analysis (Fig. S12). Given that BDM91531 alone exhibits an MIC_90_ of 62 µM, any growth inhibition observed in the presence of antibiotics and BDM91531 at a maximal concentration of 5 µM in the subsequent experiments is likely attributable to efflux pump inhibition rather than to a direct antibacterial effect of BDM91351 (Table 1). In the presence of ERY and absence of BDM91531, all AcrB variants tested (except D407N and D408A) conferred wildtype resistance and hence display a comparable growth curve progression (Fig. S11). The wildtype reference strain *E. coli* BW25113Δ*acrB*/pET24a_*acrB* showed a clear BDM91531 concentration-dependent growth inhibition when ERY was present, with growth being completely suppressed at BDM91531 concentrations above 625 nM, consistent with the observed EC_90_ value for pyridomycin (240 nM, Table 1)(Fig. S11a). Control variants D408A and D407N were inactive as shown previously^23,25^ and did not confer ERY resistance in the absence or presence of the inhibitor (Fig. S11b, c). The known BDM88855-insensitive variants A446P and S450P^19^ remained also unaffected by BDM91531 (Fig. S11d, p).

Substitutions of the acidic residues E947 and D951 (E947A, E947Q, D951A and D951N) in the proximal site of the inhibitor binding pocket confer significant tolerance toward BDM91531 (Table 1, Fig. 2A, Fig. S11e-h) and growth in the presence of ERY was only slightly inhibited at the highest inhibitor concentration (5 µM). For the E947A and E947Q substitutions, this tolerance appears to result from the removal of the observed salt bridge between the amino group of the benzamine amino-substituted ring and the E947 carboxylate, leading to a loss of affinity (Table 1, Fig. 2A, Fig S11g, h). For the decrease in PyrPip affinity observed for the D951A and D951N variants (as shown in Table 1 and Fig. S11e,f), we propose that this decrease is due to the loss of the carboxylate group, which is involved in the observed salt bridge interaction between PyrPip and the proximal benzylamine group of BDM91531 in the cryo-EM structure (Fig. 2A). Notably, D951 does not directly interact with BDM91531 in the X-ray structure (Fig. 2A), unlike the observation in the cryo-EM structure. This discrepancy may be attributed to the reduced coulomb forces in the high-salt crystallization condition. Due to these conflicting observations, we hypothesized that, in addition to the direct binding of the primary amino group of the benzylamine ring, electrostatic attraction and the initial binding of the divalent cationic BDM91531 molecule to the distal site of the transmembrane inhibitor pocket might be facilitated by D951. Indeed, reintroducing carboxylates at the nearby D951A position restored concentration dependent sensitivity towards BDM91531 for the combinatorial variants Q437E_D951A and D951A_K955E (Fig S11m, n). Introduction of additional negative charge to wildtype AcrB at the cytoplasmic rim, as in the variants Q437E or K955E, resulted in BDM91531 inhibition stronger than that observed for wildtype AcrB (Fig. S11i, j). This inhibitor hypersensitivity indicates an improved accessibility of the divalent cation BDM91531 due to electrostatic attraction (Fig. 5).

Another determinant for inhibitor access is F948. The F948A variant exhibited hypersensitivity to BDM88855. This was interpreted as the phenyl ring causing steric hindrance for the inhibitor to enter the binding pocket^19^. For BDM91531, we observed a moderate reduction in sensitivity toward the F948I and F948A variants (Fig. S11k, o). BDM91531 directly interacts with F948 through its benzylamine group (Fig. 2B), which is absent in BDM88855. Consequently, the removal of the F948 phenyl ring is likely to impact BDM91531 binding affinity (Fig. S11). In sum, acidic residues in the inhibitor binding pocket and at the cytoplasmic rim of the pocket facilitate the entry and binding of the divalent cationic inhibitor through electrostatic interaction. Removing positive charge or introducing negative charge at the rim can increase the inhibitor’s efficacy, as it directly affects the electrostatics at the entry site of the pocket.

To investigate the impact of AcrB point mutations on BDM91531 binding, we generated mutant complexes by homology modelling and refined the top model structures through short (50 ns in length) Molecular Dynamics (MD) simulations. Next, we performed a cluster analysis on the equilibrium trajectory to identify most populated conformations. Finally, we performed MM-GBSA calculations on each top cluster representative to estimate (pseudo) binding free energies. The wild-type AcrB exhibited a binding free energy (ΔG_binding_) of −41.9 kcal/mol, with key contributions from residues Glu947, Leu404, Leu442, Asp951, and Phe948 (Fig. 5J and Table S8). Several single and double mutations modulated binding affinity by altering the interaction networks, which could help rationalizing changes in BDM91531 susceptibility. Substitutions Q437E and K955E enhanced binding affinity compared to wild-type AcrB (Fig. 5J and Table S8). Structural analyses also revealed that the Q437E mutation introduced a new stable H-bond between E437 and BDM91531 (58% occupancy), as well as a salt bridge between this residue and K955 (Figs. S13, S14). Similarly, K955E slightly reinforces H-bonding of BDM91531 with E947 (which overall contributes to the binding free energy by −6.5 kcal/mol) (Table S8). Conversely, intermediate-sensitive variants like F948A and insensitive variants like D951A show weakened binding affinities (Table S8) and disrupted key interactions (Fig. S13). While our semi-quantitative binding free energy estimations rationalized most observed susceptibilities changes relative to the WT transporter, some reduced-susceptibility mutants like E947A were difficult to interpret. This prompted us to also investigate role of the putative cytoplasmic entrance gate by performing docking calculations on this region of AcrB, using structural ensembles derived from MD simulations (see *Methods* and Fig. S15 for details). Interestingly, the binding free energy calculated at the cytoplasmic gate was higher than that at the binding site in the E947A variant, suggesting (suboptimal) ligand retention away from the inhibitor binding site (Fig. S16, Fig. 5J). A similar scenario occurred for F948A. In contrast, highly susceptible single mutants Q437E and K955E and WT showed optimized binding affinities at the binding site coupled with favourable accessibility profiles (Fig. S16, Fig. 5J). Regarding the double variants Q437E_D951A and D951A_K955E, the compensatory substitutions restore in both systems an interaction with the binding site similar to that found in the wildtype transporter, although the binding to the gate entrance was weakened in the second system (Table S8).

Overall, the evaluation of (pseudo) binding affinities at both the binding and the putative cytoplasmic gate complements experimental data and provides a reasonable rationale for the susceptibility changes in all mutants. These results show the importance of negatively charged residues such as E947, D951 or alternatively Q437E and K955E at the cytoplasmic gate of the binding site of PyrPip ligands. In contrast, removal of polar or charged side chains generally impairs binding stability.

## Discussion

Previously acquired structural insights^19^ were leveraged to substantially enhance the design of an allosteric inhibitor targeting the major antibiotic efflux pump, AcrAB–TolC.

Our inhibitor design was based on the AcrB/DARPin/BDM88855 co-structure revealing E947, D951 and F948 as potential additional binding partners (Fig. 1). Consequently, the introduction of a benzylamine substituent in BDM91531, which contains a charged primary amino group, significantly enhanced its binding affinity by a factor of about fifty, and its potency by a factor of fifteen compared to BDM88855. This increase in affinity correlates with the observed two-point suspension for BDM91531 within the AcrB TMD binding pocket, involving the carboxylates D408 and E947 (Figs. 1, 2). The interaction between the primary amino group of the benzylamine ring of BDM91531 and E947 at the entrance of the inhibitor binding pocket contributes substantially to the increased binding affinity (Fig. 3). This additional ionic interaction at the cytoplasmic rim stabilizes a more defined inhibitor binding position. However, compared to the strong interaction observed between D408 and the piperazine moiety, the weaker electron densities for E947 and the benzylamine group suggest a slightly higher flexibility for the proximal benzylamine part of the BDM91531 molecule within the AcrB TMD transition zone towards the cytoplasm. In addition, the van der Waals interactions between the inhibitor’s hydrophobic surface and the hydrophobic residues within the binding pocket are enhanced in case of BDM91531 binding (Fig. 2B). The F948 interaction with the benzylamine ring of the PyrPip inhibitor further contributes to affinity (Fig. 3, Table S3). Our DSF-based binding studies indicated a substantial increase in affinity for BDM91531 (approx. 150 nM) compared to BDM88855 (approx. 5–10 µM)^19^(Fig. 3), in line with the observed EC_90_ values of 0.24 µM vs. 3.6 µM, respectively (Table 1).

A significant contribution to the affinity for the divalent cation BDM91531 is further attributed to the carboxylates at the cytoplasmic rim of the AcrB TMD (Fig. 3, Fig. 5, Fig. S11). Substitution of the inhibitor insensitive D951A variant with a secondary site carboxylate regained inhibitor sensitivity. Introducing an additional carboxylate to the AcrB wildtype conferred inhibitor hypersensitivity (Fig. 3, Fig. 5, Fig. S11). Clearly, electrostatic interactions mediated via D951 and possibly via E947 appear not only to support binding in the inhibitor binding pocket but appear to be inhibitor interaction determinants in the proximal transition zone at the cytoplasmic rim of the AcrB TMD. Electrostatic attraction likely increases the availability of the inhibitor in the transition zone and reduces the energetic profile of possible transition states. While the exact location of the carboxylate within this PyrPip transition zone appears less critical, the presence of negative charge in this region is required for inhibitor uptake. This mechanism and location-indifferent charge-dependence for inhibitor uptake has been previously described for the uptake of cationic drugs in the MFS drug/ H^+^ antiporter MdfA^26,27^, where additional carboxylate groups facilitated the entry of the cationic drug molecule via the MdfA cytoplasmic rim. The mechanism displays congruent characteristics as the exact location of the carboxylate group is rather irrelevant if it is within an otherwise large hydrophobic area.

To complete this picture, processes such as the release of protons and the uptake of inhibitors might also be seen from the perspective of water availability and dynamics. The presence of ionic side chains is supposed to increase the availability of water molecules in the transition zone (and the binding pocket). We would expect that water molecules counteract the formation of tight hydrophobic and ionic interactions within the protein, thus enabling the flexibility important for inhibitor uptake both through local transient interactions and at the global conformational level.

The main inhibitor binding determinant is, however, residue D408, which is also a key player in the H^+^-transfer through the AcrB TMD. Substitution of the carboxylate moiety by carboxamide (D408N) resulted in a complete loss of inhibitor affinity (Fig. 3). The BDM91531 interaction with D408 represents an essential salt bridge between the protonated secondary nitrogen atom (–NH_2_^+^–) of the piperazine moiety and the charged D408 carboxylate (Fig. 2A). This ionic interaction likely mimics the protonation state of D408 in the O conformer. In the same BDM91531-bound conformer, the primary amino group (–NH_3_^+^) of the K940 side chain is likely to play a similar role in the interaction with the charged D407. Both D407 and D408 might therefore be kept in a quasi-protonated state, preventing the completion of the O to L transition.

The role for D951 in inhibitor binding is less clear, as in the X-ray structure the clear density reveals no interaction with the inhibitor, whereas the cryo-EM electron density map (class I) indicates an alternate rotamer state of D951 oriented toward the benzylamine group at a distance of 3.3 Å (Fig. 2A). Possible reasons for this discrepancy might be the higher salt concentration in the crystal compared to the cryo-EM sample indicating a reduction of Coulomb forces in the first. MD simulations appear to indicate that indeed BDM91531 interacts preferably via hydrogen-bonding ( ~ 85% occupancy) with D951 (Fig. S13, Fig. S14). Both the X-ray and cryo-EM class I co-structures show the TMD of protomer A binding the BDM91531 inhibitor adopting a trapped transitional O/L conformational state (Fig. S5b, c). In contrast to the PD of protomer A in the X-ray structure adopting the well-known L state, the PD of the cryo-EM class I structure shows a trapped transitional O/L conformation. The crystallization of the AcrB/BDM88855 co-structure was facilitated by DARPins which might have a conformational selection bias toward the L state. The O/L intermediate state of the PD was also observed in the structure of the double Cys-substituted cross-linked AcrB variant (AcrB_S562C_T837C/DARPin). Comparison of the RMSD´s of the BDM91531-bound and crosslinked protomers suggests conformational trapping of the PC2 subdomain by either BDM91531 or the Cys-crosslink into a similar transition state during the conformational cycling of AcrB. The trimeric AcrB structures in complex with PyrPip also show a T conformer without drugs nor inhibitor bound. These apo structures are congruent to the previous T conformer structures (Table S4), with minocycline bound in the deep binding pocket. Since the conformation of the T protomer, including the deep binding pocket, is not affected by PyrPip binding, we assume that drug substrate binding will occur, if drug substrate is present.

The hypothesis on the inhibition of the AcrAB-TolC efflux pump by the PyrPip compounds is that one protomer is trapped in an O to L transition state and is refrained from rotating further through the L, and subsequent T and O states. Since the protomers within the AcrB trimer are cycling interdependently through these states, the entire trimer is thereby halted and catalytically inactive. The observed binding of a second BDM91531 molecule to a neighbouring protomer is possibly indicating a remaining conformational flexibility of the protomers even after the initial inhibitor binding to the first protomer. This flexibility might not allow for a full transition of conformational state (O to L), but might allow for occasional transition state conformation, so that the second inhibitor molecule can bind to the second protomer. We assume that the second protomer cannot adopt or remain the same O/L conformation as seen for the first protomer and it rather adopts an almost O state, with exception of the TMs 1, 4, 5, and TM8-11. These helices have to change conformation to allow inhibitor binding. Within one AcrB sample we observe about two-third of particles with one inhibitor bound, and one-third of the particles with two inhibitor molecules bound, hence we assume a considerably lower affinity for the second protomer inhibitor binding site.

During the LTO cycling, the O to L transition results in proton release from the titratable D407 and D408 residues to the cytoplasm^21,28^. To facilitate the proton release, a water-filled cavity, open toward the cytoplasm, is formed in the TMD between TM4, TM5 and TM10 during the O to L transition. Upon proton-release, residues D407 and D408 become negatively charged, facilitating BDM91531 binding. In the BDM-bound O-L trapped transition state, this water-filled cavity is expanded by slight further displacement of TM4, TM5, TM10 and TM11 to form an elongated pocket (Fig. 4D). We assume this expansion occurs during the O to L transition as a direct consequence of BDM91531 binding. The effect of BDM91531 binding into the pocket is the donation of a H^+^ from the piperazine amine to the D408 carboxylate anion, which consequently retains the proton. The electrostatics remain therefore balanced as apparent in the native O state and counteracts a complete transition of the O to L state, which requires the release of the proton from D408 to the cytoplasm.

In summary, BDM91531 is a nanomolar-affinity inhibitor binding to the TMD of AcrB. Its mode of action likely involves stabilization of the O to L transition state after release of protons from the TMD to the cytoplasm. Stalling of one protomer in this intermediate state appears to disrupt the functional rotation of the AcrB trimer, thereby inhibiting its catalytic activity and restoring drug susceptibility.

## Methods

### Construction of acrB chromosomal mutants

Clean Glu947Gln and Asp951Asn chromosomal mutations in *acrB* of *E.coli* strain BW25113 were constructed as described previously^29^, resulting in strains that were named BW25113 AcrB-Glu947Gln and BW25113 AcrB-Asp951Asn, respectively.

### Determination of antibiotic minimal inhibitory concentration and PyrPip EC_90_

To assess the spectrum of antibiotics boosted by BDM91531, the antibiotic MIC was determined in the presence and absence of the EPI as described previously in ref. ^19^. Briefly, a log phase culture of *E. coli* BW25113 (or isogenic mutants) was diluted to an OD_600_ = 0.001 in Cation Adjusted Muller-Hinton Broth (CAMHB, Difco) and spiked with or without BDM91531 (7.5, 15 or 30 µM). The bacterial suspension was then transferred to a 96-well microtitre plate (transparent flat-bottomed microtitre plate, Falcon) and subjected to serial dilutions of indicated antibiotics. Plates were incubated at 37 °C for 5 h and bacterial viability was evaluated by the addition of resazurin (1 h) and the measurement of resazurin turnover using a fluorescence plate reader (Tecan M-Plex, Ex: 530 nm Em: 590 nm). MIC_95_ were defined as the antibiotic concentration that prevented 95% of resazurin turnover compared to the non-treated bacteria.

### Evaluation of AcrB-Glu947Gln and Asp951Asn on BDM91531 efficacy

To evaluate the potency of PyrPip EPIs, the concentration-dependent impact of these inhibitors was measured on bacteria (*E. coli* strain BW25113, BW25113 AcrB-Glu947Gln and BW25113 AcrB-Asp951Asn) growing in the absence and presence of a sub-inhibitory concentration of the antibiotic pyridomycin (5 μg/mL). To achieve this, log-phase bacteria were diluted to an OD_600_ = 0.001, in Cation Adjusted Muller-Hinton Broth (CAMHB, Difco), spiked with pyridomycin (5 μg/mL), and subjected to serial dilutions of the PyrPip EPIs in a 96-well microplate. Plates were incubated (37 °C for 4.5 h) and bacterial viability determined by the addition of resazurin (37 °C for 1 h) and read using a fluorescence plate reader (Tecan M-Plex, Ex: 530 nm Em: 590 nm). The EPI EC_90_ was defined as the compound concentration that prevent 90% of resazurin turnover compared to the non-treated bacteria. All compounds were also tested in the absence of pyridomycin to determine their potential intrinsic antibacterial activity.

### Expression and purification *E. coli* AcrB wildtype and the AcrB_S562C_T837C variant

In brief, 6 x 1 L LB medium supplemented with 1 mM MgSO_4_ and 50 µg/mL kanamycin was inoculated 1:400 with an *E. coli* C43(DE3)*ΔacrAB*/pET24_*acrB*^30^ overnight culture grown from a single colony. Cultures were grown in 5 L shake flasks at 37 °C (135 rpm) until OD_600nm_ reached 0.8, stored on ice for 30 min, induced with 0.5 mM (final) IPTG and incubated for further 20 h at 20 °C (135 rpm). All following purification steps were performed at 4 °C. Cells were harvested (6000 x g, 20 min) and resuspended in 3 volumes (w:v, final) lysis buffer (20 mM TRIS pH 8.0, 500 mM NaCl, 2 mM MgCl_2_) supplemented with each 10 mg/L lysozyme and DNase for at least 30 min. The protease inhibitor PMSF (200 µM, final) was added directly before cell disruption with a pressure cell homogenizer (Stansted Fluid power Ltd., UK), 2x times at approx. 15.000 psi. The lysate was cleared from debris at 20.000 x g for 20 min before subjected to preparative ultra-centrifugation at 142.000 x g for 60 min. Membranes were resuspended in resuspension buffer (20 mM TRIS pH 7.5, 300 mM NaCl, 10% glycerol) to a final concentration of 0.2 mg/mL, snap frozen in liquid nitrogen and stored at −80 °C.

Purification of the wildtype protein both for crystallization and cryo-EM was carried out as follows: 16 mL of AcrB WT membrane suspensions (3.2 g) were thawed and solubilized in 1% DDM (w:v, 20% stock, (D-97002-C, Glycon)) and 12 mM imidazole (1 M stock, pH 7.5) in a final volume of 48 mL for 1 h under mild sample rotation. Buffer P1 (20 mM TRIS pH 7.5, 300 mM NaCl, 0.02% DDM) was used for volume adjustment. After ultra-centrifugation (160.000 x g, 30 min) the supernatant was rotated with 1 mL (2 mL 1:1 suspension) HisPur Ni-NTA resin (Thermo Scientific) for 30 min. The resin was washed in a gravity flow column with each 15 CV (15 mL) of P1 buffer supplemented with (i) 20 mM imidazole and (ii) 60 mM imidazole. The protein was eluted with 2 × 5 mL P1, 300 mM imidazole in a further volume of 8 mL P1. For crystallization in complex with DARPin eluates were concentrated in an Amicon Ultra-100 kDa cutoff spin concentrator to approx. 4.5 ml before the protein yield was determined (A280nm) and DARPin^9^ was added in a 1 : 1 molar ratio. The sample was incubated on ice for 10 min, further concentrated (in the same 100 kDa concentrator) to a volume of approx. 600 µl and applied on a Cytiva Superose 6 increase 10/300 GL size exclusion column with GF buffer (20 mM TRIS pH 7.5, 150 mM NaCl, 0.03% DDM, 0.05% DDAO (Anatrace)) at a flow rate of 0.25 mL/min. For cryo-EM, the sole eluate was concentrated and applied on a Cytiva Superose 6 increase 10/300 GL size exclusion column with GF2 buffer (20 mM HEPES pH 7.5, 150 mM NaCl, 0.02% DDM). Trimeric protein fractions were collected and concentrated in Amicon Ultra-100 kDa cutoff spin concentrators. Crystallization samples were adjusted to a protein concentration of 24 mg/mL (200 µl). Cryo-EM samples were adjusted to 3.2 mg/ml.

Membranes containing the AcrB_S562C_T837C variant were solubilized in 20 mM Tris, 150 mM NaCl, 10 mM imidazole, 10% glycerol and 2% DDM, pH 7.5. The soluble fraction was applied to a Ni-NTA affinity column (Ni-NTA Agarose Qiagen, 2 ml bed volume) pre-equilibrated in buffer C1 (20 mM Tris, 150 mM NaCl, 10 mM imidazole, 10% glycerol, pH 7.5). The column was washed with 45x column volume (CV) buffer C1 and subsequently washed with 30x CV buffer C2 (20 mM Tris, 150 mM NaCl, 50 mM imidazole, 10% glycerol, pH 7.5). AcrB was eluted in buffer C3 (20 mM Tris, 150 mM NaCl, 200 mM imidazole, 10% glycerol, pH 7.5). DARPins were added to the purified AcrB in a two-fold molar excess. Concentrated samples were subjected to size-exclusion chromatography (20 mM Tris, 150 mM NaCl, 0.03% DDM, pH 7.5) using a GE Superdex 200 10/300GL column coupled to a GE Healthcare AKTAprime system (at 0.5 ml/min flow rate). Peak fractions corresponding to the AcrB/DARPin complex were collected and concentrated. Concentration steps were performed in centrifugal filter devices with a cutoff size of 100 kDa (Amicon Ultra, Millipore). During concentration, buffer exchange to HEPES buffer (10 mM Na-HEPES, 50 mM NaCl, 0.03% DDM, pH 7.5) was performed.

### Expression and purification of DARPin

A volume of 50 ml LB liquid medium supplemented with 50 µg/ml carbenicillin was inoculated with a single colony of *E. coli* XL1-Blue/pQE30_110819 (DARPin)^9^ and grown overnight at 37 °C and 180 rpm. Two Tuneair^TM^ 2.5 L shake flasks each containing 1 L 2YT medium (20 g/L tryptone, 10 g/L yeast extract, 5 g/L NaCl) supplemented with 50 µg/ml carbenicillin were inoculated 1:100 and grown at 37 °C and 125 rpm until OD_600_ reached 1.0 (3.5 h) before gene expression was induced with 0.5 mM isopropyl-β-D-thiogalactopyranosid (IPTG). After further incubation for 3 h, cells were cooled down on ice and harvested (6000 x g, 4 °C, 20 min). The cell pellet (meanwhile stored at −20 °C) was resuspended in 4 volumes of lysis buffer (20 mM TRIS pH 8.0, 500 mM NaCl, 2 mM MgCl_2_) supplemented with each 10 mg/L lysozyme and DNase for 40 min at 4 °C. Protease inhibitor PMSF (200 µM, final) was added directly before cell disruption with a pressure cell homogenizer (Stansted Fluid power Ltd., UK), 3x times at approx. 15,000 psi. The lysate was cleared from debris at 30.000 x g for 45 min at 4 °C before subjected to immobilized metal affinity chromatography (IMAC). The supernatant ( + 20 mM imidazole) was added to 2.5 ml mL (5 mL 1:1 suspension) HisPur Ni-NTA resin (Thermo Scientific) and rotated for 30 min. The resin was washed in a gravity flow column with each 2 × 5 CV (12.5 mL) of P0 buffer (20 mM TRIS pH 7.5, 300 mM NaCl) supplemented with (i) 20 mM imidazole and (ii) 50 mM imidazole. The protein was eluted with 12.5 mL P0, 300 mM imidazole in 7.5 ml P0. The eluate was concentrated in a Amicon Ultra-100 kDa cutoff spin concentrator to 3.5 ml. A volume of 3 ml was rebuffered in P0 on a 10DG desalting gravity flow column (Biorad) according to the manufacturer’s instructions (equilibrate with 20 ml P0 – apply sample and discard flowthrough – elute with 4 ml P0). The purified protein was adjusted to 4 mg/ml. Aliquots were snap frozen in liquid nitrogen and stored at −80 °C.

### AcrB/DARPin/BDM91531 and AcrB_S562C_T837C/DARPin co-crystallization

AcrB/BDM91531 co-crystallization was performed in a 24-well ASV sub screen^19^ with 950 µL reservoir solution containing 50 mM ADA pH 6.6, 5% glycerol, 110–220 mM ammonium sulfate, and 8–9% PEG 4000. Equal volumes (20 ml) of protein (24 mg/mL) and GF buffer containing 5 mM BDM91531 ( + HCl, 100 mM DMSO stock) were mixed and incubated at RT for 10 min before (significant) precipitate was removed at 21.000 x g, 4 °C for 10 min. For each well, 1 μL of the supernatant was combined with 1 µL of the reservoir solution on siliconized glass cover slides (Jena Bioscience, CSL-107). Crystals were grown in hanging drops at 17°C. Rod shaped crystals were harvested after 8 weeks. Cryoprotection was sequentially carried out for each 30 s in 50 mM ADA pH 6.6, 160 mM ammonium sulfate, and 9% PEG 4000, 0.03% DDM, 0.05% DDAO and 2.5 mM BDM91531 supplemented with (i) 5%, (ii) 15% and (iii) 28% glycerol before crystals were snap frozen in liquid nitrogen.

Crystals of AcrB_S562C_T837C/DARPin were grown in reservoir solution (50 mM ADA pH 6.5, 5,1% glycerol, 200 mM ammonium sulfate, and 8% PEG 4000) mixed in a 1:1 ratio with the protein (11,6 mg/mL) in a hanging drop vapor diffusion setup at 17 °C. Before freezing in liquid nitrogen, crystals were cryoprotected by stepwise soaking the crystals in reservoir solution containing 0.03% DDM for 3 minutes with increasing (5% steps) glycerol concentrations up to 30%.

### Crystallographic data collection and processing

AcrB/DARPin/BDM91531 data sets were collected at the beamline P13 (MX1) at the “Deutsches Elektronen-Synchroton” (DESY), Hamburg, Germany at a wavelength of 0.99 Å. The highest (1.94 Å) resolution data set was obtained from a crystal grown in 50 mM ADA pH 6.6, 5% glycerol, 190 mM ammonium sulfate, and 9% PEG 4000, and 2.5 mM BDM91531. Reflections were recorded applying a helical (3600, 0.1°) data acquisition strategy. AcrB_S562C_T837C/DARPin datasets were collected at the beamline X06SA of the Swiss Light Source (SLS) at the Paul Scherrer Institut, Villigen, Switzerland at a wavelength of 1 Å. Data sets were processed using the XDS package^31^ for indexing and integration, Aimless for scaling and merging and Truncate^32^ to obtain structural amplitudes. The structure was determined by molecular replacement using Phaser^33^ and PDB: 7OUK^19^ as search model for the AcrB/DARPin/BDM91531 data, and the AcrB_DARPin wildtype structure (PDB: 4DX5)^22^ for AcrB_S562C_T837C/DARPin data. Models were manually (re)built in COOT^34^. Refinement was done with Phenix^35^ and in a final step by using the PDB_REDO Server^36^. The structure was validated with MolProbity^37^. Figures were made using PyMOL^38^ or ChimeraX^39^.

### Sample preparation for single-particle cryo-EM

For the *E. coli* (*Ec*) AcrB sample, a volume of 3.5 µL purified *Ec*AcrB (3.2 mg/mL in 20 mM HEPES pH 7.5, 150 mM NaCl, 0.02% DDM) containing 150 µM BDM91531 inhibitor (prepared from a 16 mM DMSO stock) was applied onto glow-discharged Quantifoil R1.2/1.3, 300-mesh Cu holey carbon grids (Quantifoil Micro Tools GmbH).

For the *K. pneumoniae* (*Kp*) AcrB sample, 3.5 µL of purified *Kp*AcrB (4.2 mg/mL in 20 mM HEPES pH 7.5, 150 mM NaCl, 0.02% DDM) containing 165 µM of BDM91288 inhibitor (prepared from a 16 mM DMSO stock) was deposited onto glow-discharged Quantifoil R1.2/1.3, 300-mesh Cu holey carbon grids (Quantifoil Micro Tools GmbH)^20^.

All samples were vitrified in liquid ethane using a Vitrobot Mark IV (Thermo Scientific, Waltham, USA) at 4 °C and 100% relative humidity. Prior to vitrification, Whatman blotting papers (grade 595) were pre-equilibrated in the Vitrobot chamber for 1 h under the same conditions. Blotting was performed with a nominal blot force of −25, a wait time of 40 s, with blotting times of 8 s and 10 s.

### Single-particle cryo-EM data collection

The *Ec*AcrB BDM91531 sample was imaged using a Titan Krios G1 cryo-TEM from Thermo Scientific (Waltham, USA), operating at a voltage of 300 kV. The microscope was equipped with a K2 Summit direct electron detector and a GIF Quantum S.E. post-column energy filter (Gatan Inc.), configured in zero-loss peak mode with a 20 eV slit width. A total of 2597 micrograph stacks, each comprising 26 frames with a frame time of 0.2 s, were recorded in counting mode. The dose rate was maintained at 9.6 (e^−^/Å^2^ s^−^^1^) and a total dose of 50 (e^−^/Å^2^ s^−1^) was applied per micrograph. Data were collected at a nominal magnification of 130,000x (1.05 Å per pixel) in nanoprobe EFTEM mode using SerialEM v3.8^40^. The defocus range was set between −0.8 and −3.5 µm, with a step size of 0.1 µm. Dose-fractionated movies of the *Kp*AcrB BDM91288 sample were acquired as described previously^20^.

### Cryo-EM data processing

Cryo-EM data were processed with CryoSPARC v4.0-4.1.2^41^. For the *Ec*AcrB BDM91531 sample, a total of 2527 micrographs were subjected to beam-induced motion correction CTF estimation using the integrated tools within CryoSPARC. Initially, autopicking was performed using the blob picker with a particle diameter range of 100–160 Å. This process generated 2D classes, which were then used to create templates for automated template-based particle picking. Following template picking, 465,620 particles were extracted. Two rounds of iterative unsupervised 2D classification were employed to eliminate false positives and low-quality particles, resulting in 175,821 particles being selected for ab initio reconstruction into three classes. After this step, 175,821 particles were chosen for further processing, including local correction for beam-induced motion and per-particle defocus adjustment. CTF refinement was performed iteratively per group, followed by non-uniform refinement with C1 symmetry applied to refine the AcrB homotrimer, yielding a 3.15 Å map. Unsupervised 3D classification into four classes identified two different conformational states of the AcrB homotrimer, with 132,458 particles (classes 0, 1, 3) and 43,363 particles (class 2) representing each state (named class I and class II, respectively). Subsequent non-uniform and local refinement with C1 symmetry applied to classes 0, 1, and 3 produced an *Ec*AcrB class I map at a global resolution of 3.23 Å, while class II was resolved at 3.52 Å (Fig. S6).

The data of the *Kp*AcrB BDM91288 sample was processed as described in Vieira Da Cruz et al^20^. However, further processing steps and unsupervised 3D classification resulted in an additional unpublished second class of particles showing congruent conformational characteristics as observed for *Ec*AcrB BDM91531 class II described above. Refinement of classes 2 and 3 yielded a *Kp*AcrB BDM91288 class II map at a global resolution of 3.42 Å (Fig. S9).

### Model building and refinement

For the model building of *Ec*AcrB classes I and II, the crystal structure of *E. coli* AcrB (PDB: 7OUK)^19^ served as the reference to create the initial models, starting with a round of real space refinement using Phenix^35^. The models were then iteratively adjusted manually in Coot^34^ and further refined through alternating cycles of Phenix real space refinement^35^ and refinement in Isolde^42^. The BDM91531 ligand descriptions were generated using the ligand builder tool in Coot. For *Ec*AcrB class I, the observed lipid density was represented by phosphatidylglycerol 10:0/22:0, with the alkyl chain length chosen to best fit the density. The final structures were validated using MolProbity^37^. The cryo-EM density maps have been deposited in the EMDB with accession numbers EMD-18780 and EMD-18782, respectively (Table S5). The atomic models for *Ec*AcrB classes I and II were also deposited in the PDB under the accession codes 8QZQ and 8QZT, respectively (Table S6).

For the model building of *Kp*AcrB class II, Swiss-Model^43^ was utilized for the initial model generation. A structural model of *E. coli* AcrB (PDB ID: 7OUK)^19^ was employed as a template to create a starting model based on the amino acid sequence of *K. pneumoniae* AcrB (FDAARGOS_775). This preliminary model was then adjusted to fit the cryo-EM map through real space refinement using Phenix^35^. The structural model was further refined through an iterative process that included manual adjustments in Coot^34^ and alternating cycles of Phenix real space refinement^35^. The ligand descriptions for the BDM91288 inhibitor were created using the Coot ligand builder. The final *Kp*AcrB class II structure was validated with Molprobity^37^. The refined structure for *Kp*AcrB class II was deposited in the PDB under accession code 8QZL and the associated cryo-EM density map was submitted to the EMDB (EMD-18777) (Tables S5, S6).

### Differential Scanning Fluorimetry Assay

Differential scanning fluorimetry (DSF) assays with purified AcrB and protein variants D408A, D408N, E947A and F948A were performed as described earlier^19^ with some modifications. In brief, pET24a_*acrB* variants (WT, D408A, D408N, E947A and F948A) were expressed in *E. coli* C43(DE3)*ΔacrAB* and membranes were prepared as described above. Starting from 8 ml membranes, protein purification was scaled down accordingly. Deviating from the procedure described above, the second washing step was carried out with buffer P1 (20 mM TRIS pH 7.5, 300 mM NaCl, 0.02% DDM) supplemented with 80 mM imidazole and the protein was eluted with 2 × 3 ml P1 containing 400 mM imidazole in 6 ml P1. Eluates were concentrated in an Amicon Ultra-100 kDa cutoff spin concentrator to approx. 700 µL and applied on a Cytiva Superose 6 increase 10/300 GL size exclusion column with 20 mM HEPES pH 7.5, 150 mM NaCl, 0.02% DDM at a flow rate of 0.25 mL/min. Trimeric protein fractions were collected, aliquoted, snap frozen in liquid nitrogen and stored at –80 °C. Purifications were carried out twice for each variant.

For DSF measurements protein aliquots were thawed rapidly in a metal block at room temperature (RT) and then stored on ice. Potential aggregates were removed at 13.000 x g, 4 °C for 10 min before the supernatant was diluted to 0.05 mg/mL (A_280nm_, Mol Ext Coeff = 1.269 l x mol^−^^1^ x cm^−^^1^). DSF measurements were prepared at RT. Volumes of each 0.5 µL (100-fold) of each inhibitor stock solutions containing a serial 2-fold dilution series (2 mM, 1 mM, 0.5 mM, 0.25 mM, 0.125 mM, 0.06 mM, 0.03 mM, 0.016 mM, 0.008 mM, 0.004 mM, 0.002 mM) of BDM91531 or DMSO (0 mM) were added to 1.5 mL tubes and diluted (1:100) with 49.5 µL of the protein (0.05 mg/mL). AcrB WT was analyzed in the presence of 10 µM to 20 nM BDM91531. BDM91531 concentrations between 20 μM and 40 nM were used for the other variants. Apo approaches (DMSO controls) were carried out in duplicate. Mixtures were incubated for 5 min before aggregates were removed at 13.000 x g for 2 min. After additional 15 min of incubation 39.5 µL of the supernatant was transferred into fresh 1.5 mL tubes provided with 0.5 µL 1 mg/mL (DMSO) of the cysteine reactive dye CPM (N-[4-(7-diethylamino-4-methyl-3-coumarinyl)phenyl]maleimide). Undissolved material was removed as described before and 30 µL of supernatant were transferred into 250 µL real time PCR tubes (Qiagen, 981005). Samples were heated from 30 °C to 80 °C with increments of 0.5 °C every 10 s while recording CMP-fluorescence (λ_ex_ 380 nm / λ_em_ 460 nm) in a Qiagen Rotor-Gene Q PCR machine. For automated data analysis (Rotor-Gene Q software) fluorescence intensities (F) were derived to the temperature (T) and maxima of dF/dT over T were given as melting temperatures (T_m_). For each variant five independent DSF measurements (*n* = 5) were conducted. For better comparability, ΔTm values (deviations from the apo controls) were determined for individual measurements and averaged with the center of the error bars as the mean value.

### Cloning of *acrB* variants

For the construction of the modular expression vector pET24a_acrB_M, a multi-fragment ligation strategy was applied for cloning of the modular expression vector pET24a_*acrB_M* in which the *acrB* sequence is assembled out of four similarly sized exchangeable modules that can be separated by the unique restriction sites NdeI(8379), NheI(876), Bsp119I(1604), NcoI(2471) and XhoI(3148). In detail, three fragments containing the desired terminal modifications were synthesized via PCR using primer pairs T7P/1.06, 1.05/1.08 and 1.07/T7T with pET24a_*acrB-fl* (containing additional unique sites Bsp119I(1604) and HindIII(1887), see Müller et al*.*^44^) as a template (see Table S7 for primer sequences). The PCR fragments were gel purified and combined with a fresh pET24a backbone via NdeI/BsaI/XhoI restriction/ligation cloning. The Type IIS restriction enzyme BsaI produces four nucleotide 5’ overhangs in a defined orientation to its non-palindromic recognition site (GGTCTCNNNNN(1/5)) and can therefore be used to join arbitrary sequences in a seamless manner. The NcoI restriction site at position 2471 was made unique removing NotI(1721).

pET24a_*acrB*_M Vector variants Q437E, A441L, E947Q, F948I, D951A, D951N, D951A_Q437E, D951A_Q437E and K955E were produced using the SOEing Overlap extension method^45^. For variants Q437E and A441L mutagenic primers (2.02, 3.02 or 2.01, 3.01) were combined with terminal Primers 2.17 (AcrB(NheI)FW2) or 1.02 (AcrB(Bsp119I)_RV) and pET24a_acrB_M as a template to produce pairs of fragments that were complementary to one another in the region of the mutation. For all other variants, pET24a_acrB_M was amplified with the respective mutagenic primers (2.05–2.16) that were combined with terminal primers 2.18 (AcrB_E734R_FW) or 2.19 (AcrB(XhoI)RV2) to produce the mutagenic fragment pairs. The mutagenic fragment pairs were combined in an equimolar ratio, extended to the respective full-length segments, and amplified with the respective terminal primers. Segments containing substitutions Q437E and A441L were introduced into pET24a_acrB_M via NheI/Bsp119I restriction/ligation cloning. All other substitutions were introduced into pET24a_acrB_M via NcoI/XhoI restriction/ligation cloning. The double substitution variant Q437E_D951A was produced by cloning the Q437E segment into pET24a_acrB_M _D951A via NheI/Bsp119I restriction and T4 DNA-ligase mediated ligation.

AcrB variants pET24a_acrB_A446P, S450P, E947A and F948A were described previously^19^.

### Growth Curve Assay

For growth curve analysis, *E. coli* BW25113Δ*acrB* cells were complemented with the pET24a_acrB variants WT, D407N, D408A, Q437E, A441L, A446P, S450P, E947A, E947Q, F948A, F948I, D951A, D951N, K955E and the double substitution variants D951A_Q437E and D951A_K955E. Chemically competent cells were transformed and plated on LB-agar supplemented with 50 µg/ml kanamycin (LB-K50), grown overnight at 30 °C and stored at 4 °C for at least 3 days before usage. Overnight cultures were grown from single colonies at 37 °C, 180 rpm for 10 to 12 h in glass tubes with 3 ml LB-K50 medium. Overnight cultures were diluted to OD_600_ = 0.018 and kept on ice before inoculation. The assay was conducted in a 96-well transparent, flat bottom multitier plate with lid (Sarstedt 82.1581.001, or similar) at 37 °C, shaking (30 s linear, 5 mm amplitude) and measuring OD_600_ every 20 min (TecanReader Spark) for 16 h. Each variant was grown in the presence of a serial dilution of 5 µM, 2.5 µM, 1.25 µM, 0.6 µM, 0.3 µM and 0 µM BDM91531 in combination with 16 µg/ml erythromycin (E16) in a total volume of 150 µl (LB-K50) starting from OD_600_ = 0.006 (50 µl inoculation volume). The assay was conducted in three biological replicates.

### Drug-agar-plate dilution assays in *E. coli*

Plate dilution assays were performed according to Lazarova et al. 2025^46^ with slight modifications. In brief, a serial dilution from of OD_600_ 1 to of OD_600_ 10^-5^ in 10-fold steps was prepared from pre-cultures of BW25113∆*acrB* cells transformed with pET24a_*acrB*, pet24a_*acrB*_D407N, pet24a_*acrB*_D408N. 3 µL of culture was spotted on LB-agar Kan^47^ plates and the desired antibiotic and inhibitor concentrations (erythromycin 2.75 µg/mL or 15 µg/mL, supplemented with 13 µg/mL BDM88855 or 12.5 µg/mL MBX3135). Plates were grown at 37 ˚C for 20 hours and imaged at 2500 DPI on a Perfection V19 flatbed Epson scanner (Epson, Portland Inc.). A control plate with only Kan^47^ was performed for each round. The experiments were conducted in triplicates. The last dilution steps showing cell growth were documented and averaged.

### Western Blot analysis

Expression levels were checked as previously described in ref. ^44^, using whole cells of the overnight cultures from the growth curve assay and a polyclonal primary α-AcrB (rabbit) antibody in combination with an alkaline-phosphatase-conjugated anti-rabbit secondary antibody (A3687; Sigma Aldrich) for immunological detection via BCIP/NBT staining reaction.

### Homology Modeling of AcrB Variants

To investigate structural changes and rearrangements associated with substitutions in AcrB, homology models of the trimeric mutant structures were constructed using MODELLER (version 10.6)^48^. The crystal structure of wild-type AcrB bound to the BDM91531 inhibitor, determined in this study, served as the template for constructing the homology models of the mutant complexes. For each mutant, structural refinement was performed using MODELLER’s *refine.slow* protocol, focusing on optimizing the local environment around the mutation site by including one residue on either side of the substituted residue.

### Parameterization of BDM91531 Inhibitor

The BDM91531 inhibitor ( + 2 charge state) was parameterized by optimizing its geometry using density functional theory (DFT) with the B3LYP functional and 6-31 + G* basis set, as implemented in Gaussian 16^49^. Partial atomic charges were derived using the restrained electrostatic potential (RESP) method at the same level of theory. These parameters were employed in subsequent MD simulations.

### Molecular dynamics simulations

Short MD simulations for structural relaxation of the trimeric AcrB mutant structures in complex with the BDM91531 inhibitor were performed in an explicit water environment using Amber 24^50^. Structures were processed with the *pdb4amber* command of AmberTools23 to assign GAFF2 atom types for ligands, remove existing hydrogens, and readded them. System setup was performed using *tleap*, applying the ff19SB force field for the protein^47^ and solvating the complex in a TIP3P cuboid box with 12.0 Å buffer in all directions from the solute atoms. Initial structural relaxation was conducted using a combination of steepest descent and conjugate gradient energy minimization via the *pmemd.cuda* program, following established protocols^19,51–55^ with restraints gradually released on all atoms, C_α_ atoms, and the backbone. The system was heated in two stages: first, from 0 to 100 K over 1 ns under constant-volume conditions with harmonic restraints (force constant, k = 1 kcal·mol^−1^·Å^−2^) applied to the heavy atoms of the protein and lipids; then, from 100 to 310 K over 1 ns under constant pressure (1 atm) with restraints (k = 2 kcal·mol^−1^·Å^−2^) on the Cα atoms to allow rearrangements. Equilibration followed in six consecutive 250-ps steps (total 1.5 ns) with a 1-fs time step, applying restraints to protein coordinates under isotropic pressure scaling via the Berendsen barostat and temperature control via a Langevin thermostat (collision frequency 1 ps^−1^). Production simulations were run for 50 ns at 310 K with a 2-fs time step in an isothermal-isobaric (NPT) ensemble, using a Langevin thermostat, anisotropic pressure scaling, and soft positional restraints (k = 0.1 kcal·mol^−1^·Å^−2^) on the Cα atoms of protein backbone to prevent large conformational deviations. Outputs were recorded every 200 ps. Long-range electrostatic interactions were computed using the Particle Mesh Ewald (PME) algorithm with a 9 Å non-bonded cutoff. Trajectories were post-processed with CPPTRAJ^56^. For wt, three additional independent 100 ns production simulations were performed using Amber24/pmemd.cuda to investigate π–π interactions between BDM91531 and F948. These simulations were clustered (by average-linkage method as mentioned below) to generate 100 representative structures, are included in Fig. S17.

### Clustering

Structural clustering was performed on the last 25 ns of each MD trajectory using the hierarchical agglomerative (average-linkage) method in CPPTRAJ, with the number of clusters set to five. Frames were pre-aligned to the binding site (within 4 Å of BDM91531), and ligand root-mean-square deviation (RMSD), excluding hydrogens, was used to measure similarity without additional ligand alignment. Representative structures were extracted by selecting the frame closest to each cluster’s centroid.

### MM-GMSA

Binding free energies were semiquantitatively estimated using Molecular Mechanics - Generalized Born Surface Area (MM/GBSA) approach^57^ as implemented in the *MMPBSA.py* tool of AMBER24 (igb=8), and following the same protocol used in our previous studies^51,58^. This approach provides an intrinsically simple method to decompose free energy of binding into contributions from single atoms, residues, and ligand-residue pairs. The solute conformational entropy contribution (TΔSconf) was not evaluated^57^. Calculations were performed on the top representative cluster (for binding) for each AcrB variant.

In these methods^57,59^ the binding free energy of each compound is evaluated as:

1 $${\Delta {\rm{G}}}_{{\rm{binding}}}={{\rm{G}}}_{{\rm{comp}}}-\left({\Delta {\rm{G}}}_{{\rm{rec}}}+{{\rm{G}}}_{{\rm{lig}}}\right)$$

with G_comp_, G_rec_, and G_lig_ being the absolute free energies of complex, receptor, and ligand, respectively. According to these schemes, the free-energy difference can also be decomposed as

2 $${\Delta {\rm{G}}}_{{\rm{binding}}}={{\Delta {\rm{E}}}_{{\rm{MM}}}+\Delta {\rm{G}}}_{{\rm{solv}}}-{{\rm{T}}\Delta {\rm{S}}}_{{\rm{conf}}}$$

where ΔE_MM_ is the difference in the molecular mechanics energy, ΔG_solv_ is the solvation-free energy, and ΔS_conf_ is the solute conformational entropy change. The first two terms (ΔE_MM_ and ΔG_solv_) were calculated with the following equations:

3 $${\Delta {\rm{E}}}_{{\rm{MM}}}={\Delta {\rm{E}}}_{{\rm{bond}}}+{\Delta {\rm{E}}}_{{\rm{angle}}}+{\Delta {\rm{E}}}_{{\rm{torsion}}}+{\Delta {\rm{E}}}_{{\rm{vdw}}}+{\Delta {\rm{E}}}_{{\rm{elec}}}$$

and

$${\Delta {\rm{G}}}_{{\rm{solv}}}={\Delta {\rm{G}}}_{{\rm{solv}},{\rm{p}}}+{\Delta {\rm{G}}}_{{\rm{solv}},{\rm{np}}}$$

E_MM_ includes the molecular mechanics energy contributed by the bonded (E_bond_, E_angle_, and E_torsion_) and nonbonded (E_vdw_ and E_elec_, calculated with no cutoff) terms of the force field. ΔG_solv_ is the solvation-free energy, which can be modeled as the sum of an electrostatic contribution (ΔG_solv,p_, evaluated using the MM-GBSA approach) and a nonpolar one (ΔG_solv,np_ = γΔ_SA_ + b, proportional to the difference in solvent-exposed surface area, Δ_SA_).

Note that the solute entropy contributions were not calculated here, as they are notoriously challenging; thus, these values represent relative binding energies rather than absolute free energies.

### Ensemble Docking at the cytoplasmic gate for accessibility

For each AcrB variant, the L state conformations from five representative clusters derived from MD were selected for ensemble docking using Glide High Throughput Virtual Screening (HTVS) mode as implemented in the Schrödinger Suite (release 2024-3)^60^. Proteins and ligands were prepared with default settings using the *Protein Preparation Wizard* and *Ligprep*, respectively, at physiological pH while maintaining the experimental protonation states of the proton-relay site of L-state^21,61–63^. A grid box was centered at (70.0, 85.0, 141.0) Å for the cytoplasmic gate with dimensions of 33 × 32 × 35 Å along the x, y, and z axes, respectively, oriented relative to the bilayer normal (Fig. S15). The grid was positioned downwards (from the TMD binding site) to avoid the D407/408 anchor site, with box dimensions tailored to accommodate a maximum ligand extension of ~18 Å, as observed in prior MD simulations (data not shown) to ensure realistic exploration of ligand interactions. Ensemble docking subsequently produced 50 ligand poses, with 10 poses generated for each of the five protein clusters per variant. Poses where the benzylamine group was oriented upwards, opposite to the experimental conformation, were excluded to avoid selecting conformations that would require a complete ligand flip. The top-scored poses for each variant were processed using AmberTools23, specifically *pdb4amber* was used to assign GAFF2 atom types to complexes, remove existing hydrogens, and regenerate them. The ff19SB force field was applied to the protein using *tleap*, and the complex was solvated in a TIP3P cuboid box with a 12.0 Å buffer in all directions from the solute atoms^47^. Structural relaxation was performed via energy minimization using steepest descent and conjugate gradient methods, with restraints progressively released on all atoms, then Cα atoms, and finally the backbone^64^. The relaxed and refined complex structures were used for GBSA analysis as described in the previous section.

### Chemical synthesis

The chemical scheme for the synthesis of compounds **2** to **11** is described in Fig. S18. LC-MS Waters system was equipped with a 2747 sample manager, a 2695 separation module, a 2996 photodiode array detector (200–400 nm) and a Micromass ZQ2000 detector (scan 100–800). XBridge C18 column (50 mm × 4.6 mm, 3.5 µm, Waters) was used. The injection volume was 20 µL. A mixture of water and acetonitrile was used as mobile phase in gradient-elution. The pH of the mobile phase was adjusted with HCOOH and NH_4_OH to form a buffer solution at pH 3.8. The analysis time was 5 min (at a flow rate at 2 mL/min), 10 min (at a flow rate at 1 mL/min) or 30 min (at a flow rate at 1 mL/min). Purity (%) was determined by reversed phase HPLC, using UV detection (215 nm). All final compounds showed purity greater than 95%. UPLC-MS Waters system was equipped with a UPLC I SMP MGR-FTN sample manager, an ACQUITY UPLC I-Class eK photodiode array detector (210–400 nm) and an ACQUITY QDa (Performance) detector (scan 30–1250). Acquity BEH C18 column (50 mm × 2.1 mm, 1.7 µm, Waters) was used. The injection volume was 0.5 µL. A mixture of water and acetonitrile was used as mobile phase in gradient-elution. The pH of the mobile phase was adjusted with HCOOH and NH_4_OH to form a buffer solution at pH 3.8. The analysis time was 5 min (at a flow rate at 600 µL/min), 10 min (at a flow rate at 600 µL/min) or 30 min (at a flow rate at 600 µL/min). Purity (%) was determined using UV detection (215 nm), and all isolated compounds showed purity greater than 95%. HRMS analysis was performed on a LC-MS system equipped with a LCT Premier XE mass spectrometer (Waters), using a XBridge C18 column (50 mm×4.6 mm, 3.5 µm, Waters). A gradient starting from 98% H_2_O 5 mM Ammonium Formate pH 3.8 and reaching 100% MeCN 5 mM Ammonium Formate pH 3.8 within 3 min at a flow rate of 1 mL/min was used. NMR spectra were recorded on a Bruker DRX-300 spectrometer. The results were calibrated to signals from the solvent as an internal reference [e.g., 2.50 (residual DMSO-*d*_*6*_) and 39.52 (DMSO- *d*_*6*_) ppm for ¹H and ¹³C NMR spectra respectively]. Chemical shifts (δ) are in parts per million (ppm) downfield from tetramethylsilane (TMS). The assignments were made using one-dimensional (1D) ^1^H and ^13^C spectra and two-dimensional (2D) HSQC-DEPT, COSY and HMBC spectra. NMR coupling constants (J) are reported in Hertz (Hz), and splitting patterns are indicated as follows: s for singlet, brs for broad singlet, d for doublet, t for triplet, q for quartet, dd for doublet of doublet, ddd for doublet of doublet of doublet, m for multiplet, δ for chemical shift, *J* for coupling constant. Flash chromatography was performed using a Puriflash PF-430 with silica gel cartridges (Buchi silica 40 µm). ELSD and UV detection (254 nm) were used to collect the desired product. Reverse flash chromatography was performed using a CombiFlash® Rf200 with C_18_ cartridges (Buchi C_18_ 40 µm). UV detection (215 and 254 nm) was used to collect the desired product.

#### Procedure A

Introduction of amine in position 6 of the quinoline by Buchwald coupling

tert-butyl 4-(6-bromo-3-chloro-2-quinolyl)piperazine-1-carboxylate **int-3** (0.14–0.35 mmol, 1.0 eq.), corresponding amine (1.5-1.6 eq.), Cs_2_CO_3_ (1.3-1.4 eq.), Xantphos (0.08-0.1 eq.), Pd_2_dba_3_ (0.04 eq.) were dissolved in dioxane (0.7–2.0 mL) under argon. The mixture was heated at 100 °C overnight, cooled to room temperature, dried under vacuum and purified by flash chromatography.

#### Procedure B

Boc-cleavage with HCl

In a round-bottomed flask containing the corresponding Boc-protected compound (0.06-0.20 mmol, 1.0 eq.) in 1,4-dioxane (0.4–1.6 mL) was added HCl 4 M in 1,4-dioxane (20-22 eq.). The mixture was stirred at room temperature overnight. The solvent was evaporated under reduced pressure, petroleum ether was added and the mixture was filtered to give the desired compounds.

#### Procedure C

Introduction of substituted phenyl in position 6 of the quinoline by Suzuki coupling

The tert-butyl 4-[3-chloro-6-(4,4,5,5-tetramethyl-1,3,2-dioxaborolan-2-yl)-2-quinolyl]piperazine-1-carboxylate **int-7** (0.23-0.26 mmol, 1.0 eq.) or tert-butyl 4-(6-bromo-3-chloro-2-quinolyl)piperazine-1-carboxylate **int-3** (0.23 mmol, 1.0 eq.), substituted phenyl bromide (1.0-1.1 eq) or substituted phenyl boronic acid (1.0 eq) and K_2_CO_3_ (1.6-1.7 eq) were dissolved in DME/EtOH/H_2_O (2/1/2, 5.0 mL) under argon. Then was added Pd(PPh_3_)_2_Cl_2_ (0.09-0.1 eq.), the suspension was heated at 90 °C under argon for 1 h to 5 h. The reaction was quenched with water, extracted twice with CH_2_Cl_2_. The organic layer was washed with brine, dried over MgSO_4_, evaporated under reduced pressure. The crude product was purified by flash chromatography.

#### 6-bromo-3-chloro-1H-quinolin-2-one (int-1)

To a solution of 6-bromo-1H-quinolin-2-one (22.3 mmol, 1 eq.) in anhydrous DMF (50.0 mL) was added NCS (1.5 eq.) and the reaction was stirred at 60 °C overnight. The DMF was evaporated and the compound was washed with water, EtOAc and MeOH and filtered under vacuum to give **int-1** as a pink solid. Yield: 73%; ^1^H NMR (300 MHz, CD_2_Cl_2_): δ 7.28 (d, *J* = 8.8 Hz, 1H), 7.67 (dd, *J* = 2.3, 8.8 Hz, 1H), 7.92 (d, *J* = 2.2 Hz, 1H), 8.27 (s, 1H), 12.42 (s, 1H) ppm; LCMS (ES + ) *m/z* [M + H]^+^ 258.

#### 6-bromo-2,3-dichloro-quinoline (int-2)

To a solution of 6-bromo-3-chloro-1H-quinolin-2-one **int-1** (16.3 mmol, 1 eq.) in POCl_3_ (28 eq.). The mixture was stirred at 100 °C for 1 h, then reaction mixture was poured on ice, basified with a saturated solution of Na_2_CO_3_ until pH= 7. The aqueous solution was extracted thrice with EtOAc. The organic layer was washed with brine, dried over MgSO_4_ and then evaporated under reduced pressure to give **int-2** as a brown solid. Yield: 73%; ^1^H NMR (300 MHz, CD_2_Cl_2_): δ 7.82 (dd, *J* = 2.0, 9.0 Hz, 1H), 7.87 (d, *J* = 8.9 Hz, 1H), 7.97 (d, *J* = 1.4 Hz, 1H), 8.20 (s, 1H, ^4^CH) ppm; LCMS (ES + ) *m/z* [M + H]^+^ 276.

#### tert-butyl 4-(6-bromo-3-chloro-2-quinolyl)piperazine-1-carboxylate (int-3)

6-bromo-2,3-dichloro-quinoline (int-2) (10.7 mmol, 1.0 eq.), Boc-piperazine (2.2 eq.) and triethylamine (2.2 eq.) were dissolved in MeCN (39.0 mL) under argon. The mixture was heated at 80 °C for 2 days, cooled to room temperature. The reaction was washed with a 1 N HCl aqueous solution, extracted twice with EtOAc. The organic layers were combined, washed with a saturated solution of NaCl, dried over MgSO_4_, evaporated under vacuum and purified by flash chromatography with a gradient of cyclohexane/EtOAc 100:0 to 90:10 to give int-3 as a pale yellow solid. Yield: 85%; ^1^H NMR (300 MHz, CD_2_Cl_2_): δ 1.47 (s, 9H), 3.41–3.45 (m, 4H), 3.58-3.62 (m, 4H), 7.67-7.68 (m, 2H), 7.80-7.81 (m, 1H), 7.99 (s, 1H) ppm; LCMS (ES + ) *m/z* [M + H]^+^ 426.

#### tert-butyl 4-[6-[3-[(tert-butoxycarbonylamino)methyl]-1-piperidyl]-3-chloro-2-quinolyl]piperazine-1-carboxylate (int-4)

The **int-4** was obtained using **procedure A**, from tert-butyl 4-(6-bromo-3-chloro-2-quinolyl)piperazine-1-carboxylate **int-3** (0.14 mmol, 1.0 eq.), tert-butyl N-(3-piperidylmethyl)carbamate (1.5 eq.), Cs_2_CO_3_ (1.4 eq.), Xantphos (0.1 eq.), Pd_2_dba_3_ (0.04 eq.) in dioxane (0.7 mL) at 100 °C overnight. The crude product was purified with a gradient of cyclohexane/EtOAc 100:0 to 70:30 to give **int-4** as a yellow solid. Yield: 42%; ^1^H NMR (300 MHz, CD_2_Cl_2_): δ 1.44 (s, 9H), 1.47 (s, 9H), 1.58-1.75 (m, 2H), 1.80-1.89 (m, 3H), 2.51-2.59 (m, 1H), 2.74-2.83 (m, 1H), 3.10 (t, *J* = 6.4 Hz, 2H), 3.30-3.34 (m, 4H), 3.57-3.67 (m, 6H), 4.73 (br s, 1H), 6.91 (d, *J* = 2.7 Hz, 1H), 7.39 (dd, *J* = 2.7, 9.3 Hz, 1H), 7.67 (d, *J* = 9.2 Hz, 1H), 7.92 (s, 1H) ppm; LCMS (ES + ) *m/z* [M + H]^+^ 560.

#### [1-(3-chloro-2-piperazin-1-yl-6-quinolyl)-3-piperidyl]methanamine;dihydrochloride (2)

The **int-4** (0.06 mmol, 1.0 eq.) was deprotected using **procedure B** with HCl 4 M (20 eq.) in 1,4-dioxane (0.4 mL) overnight to give **2** as an orange solid. Yield: 100%; Purity (215 nm) : 99%; ^1^H NMR (300 MHz, DMSO-*d*_6_): δ 1.23-1.34 (m, 1H), 1.80-1.93 (m, 3H), 2.22-2.31 (m, 1H), 2.76-2.83 (m, 2H), 2.88-3.12 (m, 2H), 3.23-3.28 (m, 4H), 3.57-3.69 (m, 5H), 3.85-3.91 (m, 1H), 7.66-7.87 (m, 3H), 8.29 (br s, 3H) 8.34 (s, 1H), 9.60 (s, 2H) ppm; ^13^C NMR (75 MHz, DMSO-*d*_6_): δ 23.0, 26.7, 33.1, 41.5, 42.5, 45.9, 51.2, 54.1, 109.6, 122.2, 123.2, 123.2, 126.4, 128.1, 137.2, 141.0, 154.4 ppm; LCMS (ES + ) *m/z* [M + H]^+^ 360; HRMS (*m/z*): [M + H]^+^ calcd. for C_19_H_27_ClN_5_ 360.1955; found 360.1941.

#### tert-butyl 4-[6-[4-[(tert-butoxycarbonylamino)methyl]-1-piperidyl]-3-chloro-2-quinolyl]piperazine-1-carboxylate (int-5)

The **int-5** was obtained using **procedure A**, from tert-butyl 4-(6-bromo-3-chloro-2-quinolyl)piperazine-1-carboxylate **int-3** (0.35 mmol, 1.0 eq.), tert-butyl N-(4-piperidylmethyl)carbamate (1.5 eq.), Cs_2_CO_3_ (1.3 eq.), Xantphos (0.08 eq.), Pd_2_dba_3_ (0.04 eq.) in dioxane (2.0 mL) at 100 °C overnight. The crude product was purified with a gradient of cyclohexane/EtOAc 100:0 to 70:30 to give **int-5** as a yellow oil. Yield: 53%; ^1^H NMR (300 MHz, CD_2_Cl_2_): δ 1.43 (s, 18H), 1.57-1.69 (m, 2H), 1.79-1.86 (m, 2H), 2.75 (td, *J* = 2.3, 12.2 Hz, 2H), 3.05 (t, *J* = 6.4 Hz, 2H), 3.30-3.34 (m, 4H), 3.57-3.61 (m, 4H), 3.74-3.80 (m, 2H), 4.70 (br s, 1H), 6.91 (d, *J* = 2.6 Hz, 1H), 7.39 (dd, *J* = 2.7, 9.3 Hz, 1H), 7.67 (d, *J* = 9.3 Hz, 1H), 7.93 (s, 1H) ppm; LCMS (ES + ) *m/z* [M + H]^+^ 560.

#### [1-(3-chloro-2-piperazin-1-yl-6-quinolyl)-4-piperidyl]methanamine;dihydrochloride (3)

The **int-5** (0.19 mmol, 1.0 eq.) was deprotected using **procedure B** with HCl 4 M (20 eq.) in 1,4-dioxane (1.5 mL) overnight to give **3** as a yellow solid. Yield: 100%; Purity (215 nm) : 99%; ^1^H NMR (300 MHz, DMSO-*d*_6_): δ 1.63-1.74 (m, 2H), 1.94-2.04 (m, 3H), 2.76-2.82 (m, 2H), 3.16-3.29 (m, 6H), 3.58-3.64 (m, 4H), 3.72-3.81 (m, 2H), 7.80-7.97 (m, 3H), 8.20 (br s, 3H) 8.40 (s, 1H), 9.51 (s, 2H) ppm; ^13^C NMR (75 MHz, DMSO-*d*_6_): δ 27.5, 32.2, 42.5, 43.2, 49.1, 122.4, 123.3, 126.1, 128.3, 136.0, 137.5 ppm; LCMS (ES + ) *m/z* [M + H]^+^ 360; HRMS (*m/z*): [M + H]^+^ calcd. for C_19_H_27_ClN_5_ 360.1955; found 360.1952.

#### tert-butyl 4-[6-[3-[(tert-butoxycarbonylamino)methyl]pyrrolidin-1-yl]-3-chloro-2-quinolyl]piperazine-1-carboxylate (int-6)

The **int-6** was obtained using **procedure A**, from tert-butyl 4-(6-bromo-3-chloro-2-quinolyl)piperazine-1-carboxylate **int-3** (0.35 mmol, 1.0 eq.), tert-butyl N-(pyrrolidin-3-ylmethyl)carbamate (1.6 eq.), Cs_2_CO_3_ (1.3 eq.), Xantphos (0.08 eq.), Pd_2_dba_3_ (0.04 eq.) in dioxane (2.0 mL) at 100 °C overnight. The crude product was purified with a gradient of cyclohexane/EtOAc 100:0 to 80:20 to give **int-6** as a yellow oil. Yield: 86%; ^1^H NMR (300 MHz, CD_2_Cl_2_): δ 1.43 (s, 9H), 1.47 (s, 9H), 1.74-1.86 (m, 1H), 2.11-2.22 (m, 1H), 2.50-2.60 (m, 1H), 3.08-3.52 (m, 10H), 3.57-3.61 (m, 4H), 4.78 (br s, 1H), 6.53 (d, *J* = 2.6 Hz, 1H), 7.09 (dd, *J* = 2.7, 9.2 Hz, 1H), 7.67 (d, *J* = 9.2 Hz, 1H), 7.89 (s, 1H) ppm; LCMS (ES + ) *m/z* [M + H]^+^ 546.

#### [1-(3-chloro-2-piperazin-1-yl-6-quinolyl)pyrrolidin-3-yl]methanamine;dihydrochloride (4)

The **int-6** (0.17 mmol, 1.0 eq.) was deprotected using **procedure B** with HCl 4 M (20 eq.) in 1,4-dioxane (1.3 mL) overnight to give **4** as a red powder. Yield: 100%; Purity (215 nm) : 99%; ^1^H NMR (300 MHz, DMSO-*d*_6_): δ 1.79-1.93 (m, 1H), 2.14-2.27 (m, 1H), 2.63-2.74 (m, 1H), 2.89-2.96 (m, 2H), 3.16-3.56 (m, 12H), 6.72 (d, *J* = 2.4 Hz, 1H), 7.18 (dd, *J* = 2.5, 9.2 Hz, 1H), 7.72 (d, *J* = 9.2 Hz, 1H), 8.23 (s, 1H), 8.33 (br s, 3H), 9.60 (br s, 2H) ppm; ^13^C NMR (75 MHz, DMSO-*d*_6_): δ 28.7, 36.5, 41.3, 42.6, 46.3, 47.1, 51.3, 103.4, 119.3, 122.2, 127.5, 127.7, 136.1, 137.4, 145.0, 152.0 ppm; LCMS (ES + ) *m/z* [M + H]^+^ 346; HRMS (*m/z*): [M + H]^+^ calcd. for C_18_H_25_ClN_5_ 346.1798; found 346.1805.

#### tert-butyl 4-[6-[3-[(tert-butoxycarbonylamino)methyl]phenyl]-3-chloro-2-quinolyl]piperazine-1-carboxylate (int-7)

In a round-bottomed flask was added tert-butyl 4-(6-bromo-3-chloro-2-quinolyl)piperazine-1-carboxylate **int-3** (2.34 mmol, 1.0 eq.), bis(pinacolato)diboron (2.52 mmol, 1.1 eq.), K_2_CO_3_ (7.37 mmol, 3.1 eq.), Pd(dppf)Cl_2_ (0.07 mmol, 0.03 eq.), dissolved in 50 mL of dioxane. The mixture was degassed for 40 min then heated to 100 °C. After one night, Bis(pinacolato)diboron (0.41 mmol, 0.2 eq.), K_2_CO_3_ (2.25 mmol, 1.1 eq.) and Pd(dppf)Cl_2_ (0.02 mmol, 0.01 eq.) were added then the mixture was stirred for 3 h. The reaction mixture was then washed with water and then extracted thrice with dichloromethane. The organic layers were washed with a saturated NaCl solution, dried over MgSO_4_ and evaporated under vacuum. The crude product was purified by flash chromatography (cyclohexane/EtOAc 100/0-90/10) to give the title compound **int-7**. Yield: 73%; 1H NMR (300 MHz, CD_2_Cl_2_) : δ 1.36 (s, 12H), 1.47 (s, 9H), 3.44-3.47 (m, 4H), 3.59-3.62 (m, 4H), 7.76 (d, *J* = 8.4 Hz, 1H), 7.93 (dd, *J* = 1.4, 8.4 Hz, 1H), 8.08 (s, 1H), 8.11 (br s, 1H) ppm.; LCMS (ES + ) m/z 474 (MH^+^).

#### tert-butyl N-[(2-bromophenyl)methyl]carbamate (int-8)

LiAlH_4_ (1 M in THF, 2.0 eq.) was dissolved in THF (5.0 mL) then was added dropwise a solution of AlCl_3_ (2.0 eq.) in THF/ Et_2_O (5:1, 6.0 mL). The solution was stirred at room temperature for 20 min. Then 2-bromobenzonitrile (2.8 mmol, 1.0 eq.) in THF (4.0 mL) was added dropwise. The suspension was stirred at room temperature for 3 h, then was quenched with water at 0 °C and stirred at room temperature for 1 h. The reaction was quenched with NH_3_ in H_2_O, extracted twice with CH_2_Cl_2_. The organic layer was washed with brine, dried over MgSO_4_ and evaporated under reduced pressure to afford to (2-bromophenyl)methanamine. The crude product (1.42 mmol, 1.0 eq.) was dissolved in CH_2_Cl_2_ (2.5 mL) then a solution of di-tert-butyl dicarbonate (1.3 eq.) in CH_2_Cl_2_ (2.5 mL) was added dropwise at 0°C. The reaction was stirred at room temperature for 1 h. The crude product was evaporated under reduced pressure to tert-butyl N-[(2-bromophenyl)methyl]carbamate **int-8** as a yellow oil. Yield: 62% over 2 steps; ^1^H NMR (300 MHz, CD_2_Cl_2_) : δ 1.42 (s, 9H), 4.35 (d, *J* = 6.2 Hz, 2H), 5.08 (br s, 1H), 7.12-7.19 (m, 1H), 7.31 (td, *J* = 1.2, 7.6 Hz, 1H), 7.35-7.39 (m, 1H), 7.54 (dd, *J* = 1.1, 7.9 Hz, 1H) ppm; LCMS (ES + ) m/z 286 (MH^+^).

#### tert-butyl 4-[6-[2-[(tert-butoxycarbonylamino)methyl]phenyl]-3-chloro-2-quinolyl]piperazine-1-carboxylate (int-9)

The **int-9** was obtained using **procedure C**, from tert-butyl 4-[3-chloro-6-(4,4,5,5-tetramethyl-1,3,2-dioxaborolan-2-yl)-2-quinolyl]piperazine-1-carboxylate **int-7** (0.23 mmol, 1.0 eq.), tert-butyl N-[(2-bromophenyl)methyl]carbamate (1.0 eq), K_2_CO_3_ (1.7 eq), Pd(PPh_3_)_2_Cl_2_ (0.1 eq.) in DME/EtOH/H_2_O (2/1/2, 5 mL), at 90 °C for 3 h. The crude product was purified by flash chromatography (cyclohexane/EtOAc 100/0-80/20) to give the title compound **int-9** as a white solid. Yield: 68%; ^1^H NMR (300 MHz, CD_2_Cl_2_): δ 1.43 (s, 9H), 1.48 (s, 9H), 3.43-3.47 (m, 4H), 3.61-3.65 (m, 4H), 4.26 (d, *J* = 5.9 Hz, 2H), 4.82 (br s, 1H), 7.29-7.48 (m, 4H), 7.56-7.60 (m, 2H), 7.85 (dd, *J* = 0.6, 9.2 Hz, 1H), 8.09 (s, 1H) ppm; LCMS (ES + ) m/z 553 (MH^+^).

#### [2-(3-chloro-2-piperazin-1-yl-6-quinolyl)phenyl]methanamine;dihydrochloride (5)

The **int-9** (0.20 mmol, 1.0 eq.) was deprotected using **procedure B** with HCl 4 M (20 eq.) in 1,4-dioxane (1.6 mL) overnight to give **5** as a yellow solid. Yield: 99%; ^1^H NMR (300 MHz, DMSO-*d*_6_): δ 3.26-3.32 (m, 4H), 3.66-3.70 (m, 4H), 3.93-3.98 (m, 2H), 7.37-7.41 (m, 1H), 7.46-7.56 (m, 2H), 7.72-7.80 (m, 2H), 7.89 (d, *J* = 8.7 Hz, 1H), 7.93 (d, *J* = 1.7 Hz, 1H), 8.53 (s, 1H), 8.63 (br s, 3H), 9.61 (br s, 2H) ppm; ^13^C NMR (75 MHz, DMSO-*d*_6_): δ 39.2, 42.4, 45.9, 122.0, 125.5, 127.1, 127.3, 128.1, 128.4, 128.9, 130.3, 131.5, 131.6, 136.6, 138.3, 140.6, 143.8, 155.9 ppm; HRMS (*m/z*): [M + H]^+^ calcd. for C_20_H_22_ClN_4_ 353.1533; found 353.1540

#### tert-butyl 4-[6-[3-[(tert-butoxycarbonylamino)methyl]phenyl]-3-chloro-2-quinolyl]piperazine-1-carboxylate (int-10)

The **int-10** was obtained using **procedure C**, from tert-butyl 4-(6-bromo-3-chloro-2-quinolyl)piperazine-1-carboxylate **int-3** (0.23 mmol, 1.0 eq.), [3-[(tert-butoxycarbonylamino)methyl]phenyl]boronic acid (1.0 eq), K_2_CO_3_ (1.6 eq), Pd(PPh_3_)_2_Cl_2_ (0.1 eq.) in DME/EtOH/H_2_O (2/1/2, 5 mL), at 90 °C for 1 h. The crude product was purified by flash chromatography (cyclohexane/EtOAc 100/0-70/30) to give the title compound **int-10** as a yellow oil. Yield: 55%; ^1^H NMR (300 MHz, CD_2_Cl_2_): δ 1.46 (s, 9H), 1.48 (s, 9H), 3.43-3.47 (m, 4H), 3.60-3.64 (m, 4H), 4.38 (d, *J* = 9.3 Hz, 2H), 5.04 (br s, 1H), 7.28-7.32 (m, 1H), 7.42-7.48 (m, 1H), 7.58-7.62 (m, 2H), 7.85-7.89 (m, 3H), 8.13 (s, 1H) ppm; LCMS (ES + ) m/z 553 (MH^+^).

#### [3-(3-chloro-2-piperazin-1-yl-6-quinolyl)phenyl]methanamine;dihydrochloride (6)

The **int-10** (0.13 mmol, 1.0 eq.) was deprotected using **procedure B** with HCl 4 M (20 eq.) in 1,4-dioxane (0.6 mL) overnight to give **6** as a yellow solid. Yield: 91%; Purity (215 nm) : 99%; ^1^H NMR (300 MHz, DMSO-*d*_6_): 3.25-3.30 (m, 4H), 3.65-3.70 (m, 4H), 4.11 (q, *J* = 5.6 Hz, 2H), 7.53-7.58 (m, 2H), 7.77-7.82 (m, 1H), 7.91 (d, *J* = 8.8 Hz, 1H), 8.02 (s, 1H), 8.09 (dd, *J* = 2.1, 8.8 Hz, 1H), 8.24 (d, *J* = 1.9 Hz, 1H), 8.50 (s, 1H), 8.64 (br s, 3H), 9.65 (br s, 2H) ppm; ^13^C NMR (75 MHz, DMSO-*d*_6_): δ 42.2, 42.4, 45.8, 122.0, 124.4, 125.9, 126.7, 127.7, 127.7, 128.3, 129.1, 129.3, 135.0, 136.5, 138.3, 139.3, 144.1, 155.7 ppm; LCMS (ES + ) *m/z* [M + H]^+^ 353; HRMS (*m/z*): [M + H]^+^ calcd. for C_20_H_22_ClN_4_ 353.1533; found 353.1525.

#### tert-butyl 4-[6-[4-[(tert-butoxycarbonylamino)methyl]phenyl]-3-chloro-2-quinolyl]piperazine-1-carboxylate (int-11)

The **int-11** was obtained using **procedure C**, from tert-butyl 4-(6-bromo-3-chloro-2-quinolyl)piperazine-1-carboxylate **int-3** (0.24 mmol, 1.0 eq.), [4-[(tert-butoxycarbonylamino)methyl]phenyl]boronic acid (1.0 eq), K_2_CO_3_ (1.6 eq), Pd(PPh_3_)_2_Cl_2_ (0.1 eq.) in DME/EtOH/H_2_O (2/1/2, 5 mL), at 90 °C for 3h15. The crude product was purified by flash chromatography (cyclohexane/EtOAc 100/0-80/20) to give the title compound **int-11** as a white solid. Yield: 55%; ^1^H NMR (300 MHz, CD_2_Cl_2_): δ 1.47 (s, 9H), 1.49 (s, 9H), 3.43-3.46 (m, 4H), 3.61-3.64 (m, 4H), 4.35 (d, *J* = 6.1 Hz, 2H), 5.13 (s, 1H), 7.39 (d, *J* = 8.2 Hz, 2H), 7.65 (d, *J* = 8.2 Hz, 2H), 7.83-7.87 (m, 3H), 8.10 (s, 1H) ppm; LCMS (ES + ) m/z 553 (MH^+^).

#### [4-(3-chloro-2-piperazin-1-yl-6-quinolyl)phenyl]methanamine;dihydrochloride (7)

The **int-11** (0.13 mmol, 1.0 eq.) was deprotected using **procedure B** with HCl 4 M (22 eq.) in 1,4-dioxane (0.5 mL) overnight to give **7** as a white solid. Yield: 85%; Purity (215 nm): 99%; ^1^H NMR (300 MHz, DMSO-*d*_6_): 3.26-3.30 (m, 4H), 3.64-3.69 (m, 4H), 4.08 (s, 2H), 7.64 (d, *J* = 8.4 Hz, 2H), 7.84 (d, *J* = 8.3 Hz, 2H), 7.89 (d, *J* = 8.8 Hz, 1H), 8.06 (dd, *J* = 2.1, 8.8 Hz, 1H), 8.22 (d, *J* = 2.0 Hz, 1H), 8.52 (s, 1H), 8.58 (s, 3H), 9.56 (s, 2H) ppm; ^13^C NMR (75 MHz, DMSO-*d*_6_): δ 41.8, 42.5, 45.8, 121.9, 124.4, 126.0, 126.9, 127.7, 129.1, 129.7, 133.6, 136.3, 138.3, 139.2, 144.1, 155.6 ppm; LCMS (ES + ) *m/z* [M + H]^+^ 353; HRMS (*m/z*): [M + H]^+^ calcd. for C_20_H_22_ClN_4_ 353.1533; found 353.1530.

#### tert-butyl 4-[6-(3-aminophenyl)-3-chloro-2-quinolyl]piperazine-1-carboxylate (int-12)

The **int-12** was obtained using **procedure C**, from tert-butyl 4-(6-bromo-3-chloro-2-quinolyl)piperazine-1-carboxylate **int-3** (0.23 mmol, 1.0 eq.), (3-aminophenyl)boronic acid (1.0 eq), K_2_CO_3_ (1.6 eq), Pd(PPh_3_)_2_Cl_2_ (0.1 eq.) in DME/EtOH/H_2_O (2/1/2, 5 mL), at 90 °C for 4h30. The crude product was purified by flash chromatography (cyclohexane/EtOAc 100/0-70/30) to give the title compound **int-12** as a yellow oil. Yield: 51%; ^1^H NMR (300 MHz, CD_2_Cl_2_): δ 1.48 (s, 9H), 3.42-3.47 (m, 4H), 3.60-3.65 (m, 4H), 3.87 (br s, 2H), 6.68-6.72 (m, 1H), 6.97-6.99 (m, 1H), 7.04-7.07 (m, 1H), 7.24 (t, *J* = 7.8 Hz, 1H), 7.81-7.86 (m, 3H), 8.11 (s, 1H) ppm; LCMS (ES + ) m/z 439 (MH^+^).

#### 3-(3-chloro-2-piperazin-1-yl-6-quinolyl)aniline;hydrochloride (8)

The **int-12** (0.12 mmol, 1.0 eq.) was deprotected using **procedure B** with HCl 4 M (20 eq.) in 1,4-dioxane (0.5 mL) overnight to give **8** as a yellow solid. Yield: 91%; Purity (215 nm) : 99%; ^1^H NMR (300 MHz, DMSO-*d*_6_): 3.25-3.31 (m, 4H), 3.65-3.70 (m, 4H), 7.45 (d, *J* = 7.8 Hz, 1H), 7.64 (t, *J* = 7.8 Hz, 1H), 7.78 (s, 1H), 7.64 (d, *J* = 7.9 Hz, 1H), 7.92 (d, *J* = 8.8 Hz, 1H), 7.99 (dd, *J* = 1.8, 8.8 Hz, 1H), 8.16 (d, *J* = 1.7 Hz, 1H), 8.58 (s, 1H), 9.57 (s, 2H) ppm; ^13^C NMR (75 MHz, DMSO-*d*_6_): δ 42.5, 45.8, 121.5, 122.0, 122.5, 124.6, 125.9, 126.4, 127.9, 128.9, 130.5, 132.9, 135.6, 138.5, 140.7, 144.2, 155.8 ppm; LCMS (ES + ) *m/z* [M + H]^+^ 339; HRMS (*m/z*): [M + H]^+^ calcd. for C_19_H_20_ClN_4_ 339.1376; found 339.1385.

#### tert-butyl 4-[3-chloro-6-[3-(hydroxymethyl)phenyl]-2-quinolyl]piperazine-1-carboxylate (int-13)

The **int-13** was obtained using **procedure C**, from tert-butyl 4-(6-bromo-3-chloro-2-quinolyl)piperazine-1-carboxylate **int-3** (0.23 mmol, 1.0 eq.), ([3-(hydroxymethyl)phenyl]boronic acid (1.0 eq), K_2_CO_3_ (1.6 eq), Pd(PPh_3_)_2_Cl_2_ (0.09 eq.) in DME/EtOH/H_2_O (2/1/2, 5 mL), at 90 °C for 5 h. The crude product was purified by flash chromatography (cyclohexane/EtOAc 100/0-60/40) to give the title compound **int-13** as a colorless oil. Yield: 29%; ^1^H NMR (300 MHz, CD_2_Cl_2_): δ 1.48 (s, 9H), 1.91 (t, 1H), 3.43-3.47 (m, 4H), 3.61-3.64 (m, 4H), 4.77 (d, *J* = 5.7 Hz, 2H), 7.38 (d, *J* = 7.7 Hz, 1H), 7.48 (t, *J* = 7.6 Hz, 1H), 7.62 (td, *J* = 1.6, 8.0 Hz, 1H), 7.70-7.72 (m, 1H), 7.86-7.93 (m, 3H), 8.13 (s, 1H) ppm; LCMS (ES + ) m/z 454 (MH^+^).

#### [3-(3-chloro-2-piperazin-1-yl-6-quinolyl)phenyl]methanol;hydrochloride (9)

The **int-13** (0.07 mmol, 1.0 eq.) was deprotected using **procedure B** with HCl 4 M (20 eq.) in 1,4-dioxane (0.4 mL) overnight to give **9** as a yellow powder. Yield: 100%; Purity (215 nm): 99%; ^1^H NMR (300 MHz, DMSO-*d*_6_): 3.26-3.32 (m, 4H), 3.63-3.68 (m, 4H), 4.60 (s, 2H), 7.36 (d, *J* = 7.7 Hz, 1H), 7.47 (t, *J* = 7.6 Hz, 1H), 7.64 (d, *J* = 7.8 Hz, 1H), 7.74 (s, 1H), 7.89 (d, *J* = 8.8 Hz, 1H), 8.03 (dd, *J* = 2.1, 8.8 Hz, 1H), 8.18 (d, *J* = 2.0 Hz, 1H), 8.55 (s, 1H), 9.45 (s, 2H) ppm; ^13^C NMR (75 MHz, DMSO-*d*_6_): δ 42.5, 45.9, 62.8, 121.9, 124.2, 124.9, 125.2, 125.9, 126.0, 127.6, 128.9, 129.2, 137.2, 138.4, 138.9, 143.5, 144.0, 155.5 ppm; LCMS (ES + ) *m/z* [M + H]^+^ 354; HRMS (*m/z*): [M + H]^+^ calcd. for C_20_H_21_ClON_3_ 354.1373; found 354.1392.

#### tert-butyl N-[2-(3-bromophenyl)ethyl]carbamate (int-14)

2-(3-bromophenyl)ethanamine (1.25 mmol, 1.0 eq.) was dissolved in THF/H_2_O (2.2 mL) then a solution of di-tert-butyl dicarbonate (1.6 eq.) and NaHCO_3_ (2.0 eq.) were added and the reaction was stirred at room temperature for 3 h. The reaction was quenched with water, extracted twice with EtOAc. The organic layers were combined, washed with a saturated solution of NaCl, dried over MgSO_4_, evaporated under vacuum and purified by flash chromatography (cyclohexane/EtOAc 100/0-90/10) to give the title compound **int-14** as a white solid. Yield: 81%; ^1^H NMR (300 MHz, CD_2_Cl_2_): δ 1.41 (s, 9H), 2.76 (t, *J* = 7.0 Hz, 2H), 3.32 (q, *J* = 6.8 Hz, 2H), 4.58 (s, 1H), 7.13-7.22 (m, 2H), 7.34-38 (m, 2H) ppm; LCMS (ES + ) m/z 300 (MH^+^).

#### tert-butyl 4-[6-[3-[2-(tert-butoxycarbonylamino)ethyl]phenyl]-3-chloro-2-quinolyl]piperazine-1-carboxylate (int-15)

The **int-15** was obtained using **procedure C**, from tert-butyl 4-[3-chloro-6(4,4,5,5-tetramethyl-1,3,2-dioxaborolan-2-yl)-2-quinolyl]piperazine-1-carboxylate **int-7** (0.26 mmol, 1.0 eq.), tert-butyl N-[2-(3-bromophenyl)ethyl]carbamate (1.0 eq), K_2_CO_3_ (1.6 eq), Pd(PPh_3_)_2_Cl_2_ (0.1 eq.) in DME/EtOH/H_2_O (2/1/2, 5 mL), at 90 °C for 3 h. The crude product was purified by flash chromatography (cyclohexane/EtOAc 100/0-80/20) to give the title compound **int-15** as a colorless oil. Yield: 55%; ^1^H NMR (300 MHz, CD_2_Cl_2_): δ 1.40 (s, 9H), 1.48 (s, 9H), 2.85-2.91 (m, 2H), 3.37-3.47 (m, 6H), 3.60-3.64 (m, 4H), 4.65 (br s, 1H), 7.21-7.25 (m, 1H), 7.42 (d, *J* = 7.6 Hz, 1H), 7.53-7.58 (m, 2H), 7.86-7.90 (m, 3H), 8.32 (s, 1H) ppm; LCMS (ES + ) m/z 567 (MH^+^).

#### 2-[3-(3-chloro-2-piperazin-1-yl-6-quinolyl)phenyl]ethanamine;dihydrochloride (10)

The **int-15** (0.09 mmol, 1.0 eq.) was deprotected using **procedure B** with HCl 4 M (20 eq.) in 1,4-dioxane (0.7 mL) overnight to give **10** as a yellow solid. Yield: 100%; Purity (215 nm): 99%; ^1^H NMR (300 MHz, DMSO-*d*_6_): 2.98-3.05 (m, 2H), 3.08-3.16 (m, 2H), 3.26-3.31 (m, 4H), 3.65-3.70 (m, 4H), 7.31 (d, *J* = 7.6 Hz, 1H), 7.48 (t, *J* = 7.6 Hz, 1H), 7.65-7.70 (m, 2H), 7.89 (d, *J* = 8.8 Hz, 1H), 8.07 (dd, *J* = 2.1, 8.8 Hz, 1H), 8.21 (d, *J* = 2.0 Hz, 1H), 8.23 (br s, 3H), 8.53 (s, 1H), 9.60 (br s, 2H) ppm; ^13^C NMR (75 MHz, DMSO-*d*_6_): δ 33.0, 39.6, 42.5, 45.9, 121.9, 124.4, 125.3, 126.0, 127.3, 127.6, 128.2, 129.3, 129.4, 136.9, 138.3, 138.3, 139.5, 144.0, 155.6 ppm; LCMS (ES + ) *m/z* [M + H]^+^ 367; HRMS (*m/z*): [M + H]^+^ calcd. for C_21_H_24_ClN_4_ 367.1689; found 367.1681.

#### tert-butyl N-[2-(4-bromophenyl)ethyl]carbamate (int-16)

2-(4-bromophenyl)ethanamine (1.90 mmol, 1.0 eq.) was dissolved in CH_2_Cl_2_ (3.0 mL) then a solution of di-tert-butyl dicarbonate (1.0 eq.) in CH_2_Cl_2_ (3.0 mL) was added dropwise at 0 °C. The reaction was stirred at room temperature for 1 h. The crude product was evaporated under reduced pressure and purified by flash chromatography (cyclohexane/EtOAc 100/0-70/30) to give the title compound **int-16** as a white solid. Yield: 88%; ^1^H NMR (300 MHz, CD_2_Cl_2_) : δ 1.40 (s, 9H), 2.74 (t, *J* = 7.0 Hz, 2H), 3.31 (q, *J* = 6.6 Hz, 2H), 4.58 (br s, 1H), 7.09 (d, *J* = 8.4 Hz, 2H), 7.43 (d, *J* = 8.4 Hz, 2H) ppm; LCMS (ES + ) m/z 300 (MH^+^).

#### tert-butyl 4-[6-[4-[2-(tert-butoxycarbonylamino)ethyl]phenyl]-3-chloro-2-quinolyl]piperazine-1-carboxylate (int-17)

The **int-17** was obtained using **procedure C**, tert-butyl 4-[3-chloro-6-(4,4,5,5-tetramethyl-1,3,2-dioxaborolan-2-yl)-2-quinolyl]piperazine-1-carboxylate **int-7** (0.26 mmol, 1.0 eq.), tert-butyl N-[2-(4-bromophenyl)ethyl]carbamate (1.0 eq), K_2_CO_3_ (1.7 eq), Pd(PPh_3_)_2_Cl_2_ (0.1 eq.) in DME/EtOH/H_2_O (2/1/2, 5 mL), at 90 °C for 3 h. The crude product was purified by flash chromatography (cyclohexane/EtOAc 100/0-70/30) to give the title compound **int-17** as a white solid. Yield: 28%; ^1^H NMR (300 MHz, CD_2_Cl_2_): δ 1.42 (s, 9H), 1.48 (s, 9H), 2.84 (t, *J* = 7.0 Hz, 2H), 3.35-3.47 (m, 6H), 3.60-3.64 (m, 4H), 4.64 (br s, 1H), 7.32 (d, *J* = 8.3 Hz, 2H), 7.65 (d, *J* = 8.3 Hz, 2H), 7.84-7.88 (m, 3H), 8.13 (s, 1H) ppm; LCMS (ES + ) m/z 567 (MH^+^).

#### 2-[4-(3-chloro-2-piperazin-1-yl-6-quinolyl)phenyl]ethanamine;dihydrochloride (11)

The **int-17** (0.08 mmol, 1.0 eq.) was deprotected using **procedure B** with HCl 4 M (21 eq.) in 1,4-dioxane (0.6 mL) overnight to give **11** as a yellow solid. Yield: 93%; Purity (215 nm) : 99%; ^1^H NMR (300 MHz, DMSO-*d*_6_): 2.94-3.00 (m, 2H), 3.02-3.11 (m, 2H), 3.25-3.30 (m, 4H), 3.64-3.68 (m, 4H), 7.41 (d, *J* = 8.2 Hz, 2H), 7.75 (d, *J* = 8.2 Hz, 2H), 7.88 (d, *J* = 8.8 Hz, 1H), 8.03 (dd, *J* = 2.0, 9.0 Hz, 1H), 8.18 (d, *J* = 2.0 Hz, 1H), 8.21 (br s, 3H), 8.53 (s, 1H), 9.61 (br s, 2H) ppm; ^13^C NMR (75 MHz, DMSO-*d*_6_): δ 32.6, 39.6, 42.5, 45.9, 121.9, 124.1, 126.0, 127.0, 127.6, 129.1, 129.5, 136.7, 137.1, 137.6, 138.4, 143.9, 155.6 ppm; LCMS (ES + ) *m/z* [M + H]^+^ 367; HRMS (*m/z*): [M + H]^+^ calcd. for C_21_H_24_ClN_4_ 367.1689; found 367.1715.

## Supplementary information

Börnsen_et_al_supplementary_figures_and_tables_revision_4

## Data Availability

All data needed to evaluate the conclusions in the paper are present in the paper and/or the Supplementary Materials. Atomic coordinates and structure factors reported in this paper have been deposited in the Protein Data Bank under accession numbers [9HAO] (EcAcrB in complex with BDM91531, X-ray structure), [9HCI] (EcAcrB_S562_T837C PC2_TM7 crosslink X-ray structure), [8QZQ] (EcAcrB in complex with BDM91531, cryoEM-structure class I), [8QZT] (EcAcrB in complex with BDM91531, cryoEM-structure class II), and [8QZL] (KpAcrB in complex with BDM91288, cryoEM-structure class II). Atomic coordinates that were used and support the findings of this study are available in the Protein Data Bank under accession numbers as listed under Table S4, and KpAcrB in complex with BDM91288, SPA-structure, class I - [8P1I]. All supporting computational data for the binding and accessibility studies of BDM91531-AcrB variant complexes can be accessed via Zenodo (10.5281/zenodo.15668409).
