# Supplementary material for: Molecular mechanism of transition-state inhibitors of bacterial antibiotic efflux pumps
Source: NPJ Antimicrob Resist. 2026 May 4;4:35. doi: 10.1038/s44259-026-00207-6 (PMC13139501; doi:10.1038/s44259-026-00207-6)
Supplement: Supplementary file 1 — Börnsen_et_al_supplementary_figures_and_tables_revision_4 [file 44259_2026_207_MOESM1_ESM.pdf]

Supplementary Materials for

Molecular mechanism of transition-state inhibitors of bacterial antibiotic efflux pumps

Clara Börnsen, Reinke T. Müller *et al.*

*Corresponding authors Email:
reinkemueller@gmx.de
ruben.hartkoorn@inserm.fr
marion.flipo@univ-lille.fr
achilleas.frangakis@biophysik.org
pos@em.uni-frankfurt.de

This PDF file includes:

Tables S1 to S8
Figs. S1 to S18
References (1 to 6)

Table S1. Minimal inhibitory concentration of a panel of antibiotics on wildtype (WT) *E. coli* BW25113, and an isogenic strain lacking *acrA* and *acrB* (*E. coli* BW25113 Δ*acrAB*), in the presence and absence of 7.5, 15 and 30 µM BDM91531. MICs are measured after 5 h of incubation with the antibiotics using resazurin, and the MIC_{95} defined as the concentration at which there is less than 5% resorufin fluorescence compared to untreated bacteria. Data are an average (range) of at least 3 independent biological replicates.

Antibiotic	WT BW25113 MIC_{95} (µg/mL) Mean [range]				BW25113 Δ*acrAB* MIC_{95} (µg/mL) Mean [range]			
	0 µM BDM91531	7.5 µM BDM91531	15 µM BDM91531	30 µM BDM91531	0 µM BDM91531	7.5 µM BDM91531	15 µM BDM91531	30 µM BDM91531
**Oxacillin**	>200 [200->200]	4.7 [1.6-6.3]	1.3 [0.8-1.6]	0.65 [0.39-0.78]	0.78	0.52 [0.39-0.78]	0.39	0.39
**Linezolid**	83 [50-100]	2.1 [1.6-3.1]	1.6	1.6	2.3 [1.6-3.1]	1.6	1.6	1.6
**Novobiocin**	100	2.1 [1.6-3.1]	1.6	1.3 [0.8-1.6]	2.9 [1.6-3.1]	1.6	1.3 [0.8-1.6]	1.6
**Erythromycin**	33 [12.5-50]	1.0 [0.8-1.6]	0.8	1.6	2.9 [1.6-6.3]	0.9 [0.4-1.6]	0.7 [0.4-0.8]	1.6
**Tetracycline**	0.31	0.10 [0.08-0.16]	0.10 [0.08-0.16]	0.12 [0.08-0.16]	0.16	0.10 [0.08-0.16]	0.10 [0.08-0.16]	0.16
**Levofloxacin**	0.016	0.004	0.005 [0.004-0.008]	0.008	0.007 [0.004-0.008]	0.007 [0.004-0.008]	0.007 [0.004-0.008]	0.007 [0.004-0.008]
**Chloramphenicol**	2.4 [1.3-3.1]	0.31	0.31	0.39	0.35 [0.31-0.39]	0.31	0.26 [0.16-0.31]	0.39

Table S2. Data collection and refinement statistics crystallographic structures of AcrB wildtype in complex with BDM91531 and AcrB_S562C_T837C

	AcrB in complex with BDM91531 PDB 9HAO	AcrB_S562C_T837C PC2-TM7 crosslink PDB 9HCI
**Data collection**		
Space group	P 21 21 21	P 21 21 21
Cell dimensions		
a, *b*, *c* (Å)	146.18, 161.14, 244.39	145.79, 163.54, 245.14
α, β, γ (°)	90.00, 90.00, 90.00	90.00, 90.00, 90.00
Resolution (Å)	49.38 - 1.94 (2.01 - 1.94)	49.04 - 2.70 (2.80 - 2.70)
R_{sym} or R_{merge}	0.1449 (2.939)	0.1853 (1.309)
$I / \sigma I$	10.22 (0.66)	6.14 (1.01)
Completeness (%)	99.1 (93.4)	99.95 (99.99)
Redundancy	14.1 (14.5)	4.3 (3.9)
**Refinement**		
Resolution (Å)	49.38 - 1.94 (2.01 - 1.94)	49.04 - 2.70 (2.80 - 2.70)
No. reflections	419276 (39220)	160664 (15890)
R_{work} / R_{free}	0.2022 / 0.2253	0.2019 / 0.2364
No. atoms		
Protein	25923	25971
Ligand/ion	2364	566
Water	1597	1559
B-factors		
Protein	50.14	47.96
Ligand/ion	70.44	70.12
Water	48.56	42.92
R.m.s. deviations		
Bond lengths (Å)	0.004	0.003
Bond angles (°)	0.606	0.640

*Values in parentheses are for highest-resolution shell.

Table S3. Thermal shifts determined via Differential Scanning Fluorimetry (DSF). Protein melting temperatures of solubilized AcrB wildtype (WT) and protein variants D408A, D408N, E947A and F948A were determined in the presence of a concentration series of BDM91531 (0 – 20 µM). Apo melting temperatures (T_m (Apo)) of each protein variant and averaged thermal shifts (ΔT_m) from five individual measurements (n = 5) as well as the associated standard deviations are given below in °C.

BDM91531 [µM]	WT	D408A	D408N	E947A	F948A
20	-	-0.24 (±0.17)	-0.11 (±0.16)	0.72 (±0.13)	0.65 (±0.22)
10	1.89 (±0.19)	-0.06 (±0.14)	-0.13 (±0.15)	0.57 (±0.04)	0.62 (±0.15)
5	1.93 (±0.13)	0.06 (±0.08)	-0.03 (±0.08)	0.40 (±0.07)	0.60 (±0.10)
2.5	1.83 (±0.14)	0.01 (±0.08)	-0.01 (±0.07)	0.20 (±0.04)	0.45 (±0.03)
1.25	1.76 (±0.11)	-0.01 (±0.10)	0.04 (±0.10)	0.10 (±0.06)	0.38 (±0.06)
0.63	1.56 (±0.13)	0.05 (±0.11)	0.01 (±0.07)	0.05 (±0.04)	0.30 (±0.11)
0.31	1.17 (±0.09)	-0.01 (±0.16)	0.06 (±0.07)	0.04 (±0.09)	0.19 (±0.15)
0.16	0.88 (±0.03)	0.05 (±0.06)	-0.01 (±0.07)	0.04 (±0.06)	0.15 (±0.06)
0.08	0.54 (±0.06)	0.02 (±0.11)	0.02 (±0.10)	0.03 (±0.09)	0.17 (±0.08)
0.04	0.25 (±0.10)	-0.04 (±0.12)	0.02 (±0.04)	0.00 (±0.06)	0.03 (±0.05)
0.02	0.16 (±0.13)	-	-	-	-
T_m (Apo)	54.00 (±0.12)	54.89 (±0.09)	54.23 (±0.10)	54.01 (±0.09)	55.31 (±0.05)

Table S4. RMSDs of the different structures and sub-domains presented in this study. All Cα RMSDs were calculated with ChimeraX 1.7.1 [1] using the Needleman-Wunsch alignment algorithm. Structures were compared with the following published X-ray structures: EcAcrB/DARPin apo (PDB 4DX5)[2] and EcAcrB/DARPin + BDM88855 (PDB 7OUK)[3].

A. RMSDs of the entire trimers of the AcrB/DARPin/BDM91531 co-crystal X-ray structure.

**RMSD values for:**				**Compared to published structures:**		
Protein	BDM	Type	**RMSD**	Protein	Type	PDB
*Ec*AcrB/DARPin	91531	X-ray	**0.22**	*Ec*AcrB/DARPin apo	X-ray	4DX5
*Ec*AcrB/DARPin	91531	X-ray	**0.35**	*Ec*AcrB/DARPin+BDM88855	X-ray	7OUK
AcrB_S562C_T837C/ DARPin	-	X-ray	**1.63**	*Ec*AcrB/DARPin apo	X-ray	4DX5

B. RMSDs of the entire AcrB trimer cryoEM structures.

**RMSD values for:**				**Compared to:**		
Protein	BDM	Type	**RMSD**	Protein	BDM	Type
*Ec*AcrB	91531	class I	**0.99**	*Kp*AcrB	91288	class I
*Ec*AcrB	91531	class II	**0.83**	*Kp*AcrB	91288	class II

C. RMSDs of the individual AcrB protomers (A, B, C).

**RMSD values for:**					**Compared to published structures:**		
Protein	BDM	Type	Proto-mer	**RMSD**	Protein	State	PDB
*Ec*AcrB/DARPin	91531	X-ray	A	**1.00**	*Ec*AcrB/DARPin apo	L	4DX5
*Ec*AcrB/DARPin	91531	X-ray	A	**0.37**	*Ec*AcrB/DARPin+BDM88855	L[1]	7OUK
*Ec*AcrB/DARPin	91531	X-ray	B	**0.33**	*Ec*AcrB/DARPin apo	T	4DX5
*Ec*AcrB/DARPin	91531	X-ray	C	**0.22**	*Ec*AcrB/DARPin apo	O	4DX5
*Ec*AcrB_S562C_T837C/ DARPin	-	X-ray	A	**2.50**	*Ec*AcrB/DARPin apo	O	4DX5
*Ec*AcrB_S562C_T837C/ DARPin	-	X-ray	A	**1.63**	*Ec*AcrB/DARPin apo	L	4DX5
*Ec*AcrB_S562C_T837C/ DARPin	-	X-ray	B	**0.58**	*Ec*AcrB/DARPin apo	T	4DX5
*Ec*AcrB_S562C_T837C/ DARPin	-	X-ray	C	**0.41**	*Ec*AcrB/DARPin apo	O	4DX5
*Ec*AcrB	91531	class I	A	**1.05**	*Ec*AcrB/DARPin apo	O	4DX5
*Ec*AcrB	91531	class I	A	**2.05**	*Ec*AcrB/DARPin apo	L	4DX5
*Ec*AcrB	91531	class I	A	**1.61**	*Ec*AcrB/DARPin+BDM88855	L[1]	7OUK
*Ec*AcrB	91531	class I	B	**0.85**	*Ec*AcrB/DARPin apo	T	4DX5
*Ec*AcrB	91531	class I	C	**0.78**	*Ec*AcrB/DARPin apo	O	4DX5
*Ec*AcrB	91531	class II	A	**1.61**	*Ec*AcrB/DARPin apo	L	4DX5
*Ec*AcrB	91531	class II	A	**1.10**	*Ec*AcrB/DARPin+BDM88855	L[1]	7OUK
*Ec*AcrB	91531	class II	B	**0.89**	*Ec*AcrB/DARPin apo	T	4DX5
*Ec*AcrB	91531	class II	C	**1.15**	*Ec*AcrB/DARPin apo	O	4DX5
*Kp*AcrB	91288	class I	A	**3.11**	*Ec*AcrB/DARPin apo	O	4DX5
*Kp*AcrB	91288	class I	A	**2.29**	*Ec*AcrB/DARPin apo	L	4DX5
*Kp*AcrB	91288	class I	A	**1.87**	*Ec*AcrB/DARPin+BDM88855	L[1]	7OUK
*Kp*AcrB	91288	class I	B	**1.02**	*Ec*AcrB/DARPin apo	T	4DX5
*Kp*AcrB	91288	class I	C	**0.88**	*Ec*AcrB/DARPin apo	O	4DX5
*Kp*AcrB	91288	class II	A	**1.46**	*Ec*AcrB/DARPin apo	L	4DX5
*Kp*AcrB	91288	class II	A	**0.94**	*Ec*AcrB/DARPin+BDM88855	L[1]	7OUK
*Kp*AcrB	91288	class II	B	**1.05**	*Ec*AcrB/DARPin apo	T	4DX5
*Kp*AcrB	91288	class II	C	**1.23**	*Ec*AcrB/DARPin apo	O	4DX5

[1] *The L state in 7OUK means: L conformation in the PD domain and O/L conformation in the TMD domain.*

D. Root mean square deviations (RMSDs) of the individual porter and transmembrane domains (PD and TMD) of different AcrB protomer A structures.

RMSD values for:						Compared to published structures:			
Protein	BDM	Type	Proto-mer	PD/ TMD	**RMSD**	Protein	PD/ TMD	State	PDB
*Ec*AcrB/DARPin	91531	X-ray	A	PD	**0.25**	*Ec*AcrB/DARPin apo	PD	L	4DX5
*Ec*AcrB/DARPin	91531	X-ray	A	PD	**0.22**	*Ec*AcrB/DARPin +BDM88855	PD	L[1]	7OUK
*Ec*AcrB/DARPin	91531	X-ray	A	TMD	**1.35**	*Ec*AcrB/DARPin apo	TMD	L	4DX5
*Ec*AcrB/DARPin	91531	X-ray	A	TMD	**0.48**	*Ec*AcrB/DARPin +BDM88855	TMD	L[1]	7OUK
*Ec*AcrB_S562C_T837C/ DARPin	-	X-ray	A	PD	**1.66**	*Ec*AcrB/DARPin apo	PD	O	4DX5
*Ec*AcrB_S562C_T837C/ DARPin	-	X-ray	A	PD	**2.02**	*Ec*AcrB/DARPin apo	PD	L	4DX5
*Ec*AcrB_S562C_T837C/ DARPin	-	X-ray	A	PD	**1.87**	*Ec*AcrB/DARPin +BDM88855	PD	L[1]	7OUK
*Ec*AcrB_S562C_T837C/ DARPin	-	X-ray	A	TMD	**0.67**	*Ec*AcrB/DARPin +BDM88855	TMD	L	4DX5
*Ec*AcrB	91531	class I	A	PD	**1.77**	*Ec*AcrB/DARPin apo	PD	O	4DX5
*Ec*AcrB	91531	class I	A	PD	**1.93**	*Ec*AcrB/DARPin apo	PD	L	4DX5
*Ec*AcrB	91531	class I	A	PD	**1.80**	*Ec*AcrB/DARPin +BDM88855	PD	L[1]	7OUK
*Ec*AcrB	91531	class I	A	TMD	**1.98**	*Ec*AcrB/DARPin apo	TMD	O	4DX5
*Ec*AcrB	91531	class I	A	TMD	**1.52**	*Ec*AcrB/DARPin apo	TMD	L	4DX5
*Ec*AcrB	91531	class I	A	TMD	**0.70**	*Ec*AcrB/DARPin +BDM88855	TMD	L[1]	7OUK
*Ec*AcrB	91531	class II	A	PD	**0.91**	*Ec*AcrB/DARPin apo	PD	L	4DX5
*Ec*AcrB	91531	class II	A	PD	**0.85**	*Ec*AcrB/DARPin +BDM88855	PD	L[1]	7OUK
*Ec*AcrB	91531	class II	A	TMD	**1.36**	*Ec*AcrB/DARPin apo	TMD	L	4DX5
*Ec*AcrB	91531	class II	A	TMD	**0.69**	*Ec*AcrB/DARPin +BDM88855	TMD	L[1]	7OUK
*Ec*AcrB	91531	class II	C	PD	**0.68**	*Ec*AcrB/DARPin apo	PD	O	4DX5
*Ec*AcrB	91531	class II	C	TMD	**1.51**	*Ec*AcrB/DARPin apo	TMD	O	4DX5
*Kp*AcrB	91288	class I	A	PD	**1.51**	*Ec*AcrB/DARPin apo	PD	O	4DX5
*Kp*AcrB	91288	class I	A	PD	**2.16**	*Ec*AcrB/DARPin apo	PD	L	4DX5
*Kp*AcrB	91288	class I	A	PD	**2.01**	*Ec*AcrB/DARPin +BDM88855	PD	L[1]	7OUK
*Kp*AcrB	91288	class I	A	TMD	**2.30**	*Ec*AcrB/DARPin apo	TMD	O	4DX5
*Kp*AcrB	91288	class I	A	TMD	**1.97**	*Ec*AcrB/DARPin apo	TMD	L	4DX5
*Kp*AcrB	91288	class I	A	TMD	**1.36**	*Ec*AcrB/DARPin +BDM88855	TMD	L[1]	7OUK
*Kp*AcrB	91288	class II	A	PD	**1.01**	*Ec*AcrB/DARPin apo	PD	L	4DX5
*Kp*AcrB	91288	class II	A	PD	**0.94**	*Ec*AcrB/DARPin +BDM88855	PD	L[1]	7OUK

*Kp*AcrB	91288	class II	A	TMD	**1.58**	*Ec*AcrB/DARPin apo	TMD	L	4DX5
*Kp*AcrB	91288	class II	A	TMD	**0.75**	*Ec*AcrB/DARPin +BDM88855	TMD	L[1]	7OUK
*Kp*AcrB	91288	class II	C	PD	**0.73**	*Ec*AcrB/DARPin apo	PD	O	4DX5
*Kp*AcrB	91288	class II	C	TMD	**1.65**	*Ec*AcrB/DARPin apo	TMD	O	4DX5

1 *The L state in 7OUK means: L conformation in the PD domain and O/L conformation in the TMD domain.*

E. Root mean square deviations (RMSDs) of the individual porter and transmembrane domains (PD and TMD) of protomer A of the structures presented in this study.

**RMSD values for the X-ray structures from this study:**						**Compared to the cryoEM *Ec*AcrB + BDM91531 structures from this study**			
Protein	BDM	Type	Proto-mer	PD /TMD	**RMSD**	Protein	Type	Proto-mer	PD /TMD
*Ec*AcrB/DARPin	91531	X-ray	A	PD	**1.84**	*Ec*AcrB +BDM91531	class I	A	PD
*Ec*AcrB/DARPin	91531	X-ray	A	TMD	**0.49**	*Ec*AcrB +BDM91531	class I	A	TMD
*Ec*AcrB/DARPin	91531	X-ray	A	PD	**0.84**	*Ec*AcrB +BDM91531	class II	A	PD
*Ec*AcrB/DARPin	91531	X-ray	A	TMD	**0.64**	*Ec*AcrB +BDM91531	class II	A	TMD
*Ec*AcrB_S562C_T837C/ DARPin	-	X-ray	A	PD	**0.63**	*Ec*AcrB +BDM91531	class I	A	PD
*Ec*AcrB_S562C_T837C/ DARPin	-	X-ray	A	PD	**1.30**	*Ec*AcrB +BDM91531	class I	A	TMD
*Ec*AcrB_S562C_T837C/ DARPin	-	X-ray	A	PD	**1.53**	*Ec*AcrB +BDM91531	class II	A	PD
*Ec*AcrB_S562C_T837C/ DARPin	-	X-ray	A	TMD	**1.22**	*Ec*AcrB +BDM91531	class II	A	TMD

Table S5. Statistics of cryo-EM data collection and processing parameters for *Ec*AcrB in complex with BDM91531 and *Kp*AcrB in complex with BDM91288.

	***Ec*AcrB + BDM91531 class I**	***Ec*AcrB + BDM91531 class II**	***Kp*AcrB + BDM91288 class II**
**Data collection**			
Microscope	Titan Krios G2	Titan Krios G2	Titan Krios G3i
Detector	Gatan K2	Gatan K2	Gatan K3
Acquisition Software	SerialEM 3.8	SerialEM 3.8	EPU 3.1
Magnification	130,000x	130,000x	105,000x
Pixel size [Å]	1.05	1.05	0.837
Voltage [kV]	300	300	300
Total electron dose [e^-/Å^2]	50	50	50
Dose rate [Å^2/s^{-1}]	9.6	9.6	16
Number of frames	26	26	50
Frame time [s]	0.2	0.2	0.052
Defocus range [µm]	-0.8 to -3.5	-0.8 to -3.5	-0.8 to -3.5
**Processing**			
Micrographs used	2527	2527	7927
Processing software	cryoSPARC v4.0	cryoSPARC v4.0	cryoSPARC v4.1.2
Motion correction	cryoSPARC v4.0	cryoSPARC v4.0	cryoSPARC v4.1.2
CTF estimation	cryoSPARC v4.0	cryoSPARC v4.0	cryoSPARC v4.1.2
Initial particle images	465,620	465,620	1,396,333
Particle images after 2D classification	175,821	175,821	380,181
Final particle images	132,458	43,363	109,174
Symmetry	C1	C1	C1
Map sharpening B factor [Å^2]	-130	-100	-91
Map resolution range [Å]	X	X	X
Model Resolution [Å]	3.23	3.52	3.42
FSC threshold	0.143	0.143	0.143
EMDB code	EMD-18780	EMD-18782	EMD-18777

Table S6. Refinement and validation statistics for the cryoEM structures: *Ec*AcrB class I and class II in complex with BDM91531 and *Kp*AcrB class II in complex with BDM91288.

	***Ec*AcrB + BDM91531 class I**	***Ec*AcrB + BDM91531 class II**	***Kp*AcrB + BDM91288 class II**
**Refinement**			
Initial model used (PDB code)	7OUK	/	/
Model resolution [Å]	3.23	3.52	3.42
FSC threshold	0.143	0.143	0.143
Model resolution range [Å]			
**Model composition**			
Non-hydrogen atoms	47640	47376	47136
Hydrogen atoms	24058	23913	23769
Protein residues	3094	3079	3069
Ligands	2	2	2
**B-factors [Å^2]**			
Protein	85.94	99.87	34.95
Ligand	95.32	103.69	58.55
**R.m.s. deviations**			
Bond lengths [Å]	0.003	0.002	0.002
Bond angles [°]	0.477	0.477	0.452
**Validation**			
MolProbity score	1.43	1.32	1.48
Clashscore	4.98	5.89	5.82
Poor rotamers [%]	0.00	0.00	0.04
**Ramachandran plot**			
Favored [%]	97.02	98.08	97.12
Allowed [%]	2.95	1.92	2.88
Disallowed [%]	0.03	0.00	0.00
**PDB accession code**	8QZQ	8QZT	8QZL

Table S7. Used primers for cloning and mutagenesis. The index and primer names are given in the material and methods section. The bases indicated in capital letters correspond to the positions in the sequence for conversion into restriction recognition sites (primers with index 1.01 – 1.08), or for the exchange of codons as given in the primer name (primers with index 2.01 – 2.19). Primers with index 3.01 - 3.04 are sequencing primers.

Index	Primer name	Sequence	Purity
**1.01**	AcrB(Bsp119I)_FW	cgtaggcggtattcTTCGAAgtacggggcgttacc	DST
**1.02**	AcrB(Bsp119I)_RV	ggtaacgccccgtacTTCGAAgaataccgcctacg	DST
**1.03**	AcrB(HindIII)_FW	ggtattgcgttcgtAAGCTTgaaggactgggcc	DST
**1.04**	AcrB(HindIII)_RV	ggcccagtccttcAAGCTTacgaacgcaatacc	DST
**1.05**	AcrB(NheI)BsaI_FW	atatatGGTCTCtAGCGaccggtgcaaacgcg	DST
**1.06**	AcrB(NheI)BsaI_RV	atatatGGTCTCtCGCTagcttgatccccagaccggaag	DST
**1.07**	AcrB(dNcoI)BsaI_FW	atatatGGTCTCaGACGatggttcagctgccagcagg	DST
**1.08**	AcrB(dNcoI)BsaI_RV	atatatGGTCTCaCGTCataaacacgccctggtcctcatc	DST
**2.01**	AcrB_Q437E_FW	cgatggggGAAattcagggcgctctggtc	DST
**2.02**	AcrB_Q437E_RV	gccctgaatTTCcccccatcgacttacggg	DST
**2.03**	AcrB_A441L_FW	cagggcCTGctggtcggtatcgcgatg	DST
**2.04**	AcrB_A441L_RV	gataccgaccagCAGgccctgaatctgccccatc	DST
**2.05**	AcrB_E947Q_FW	ccttatcgtcCAGttcgccaaagacttgatgg	DST
**2.06**	AcrB_E947Q_RV	ctttggcgaaCTGgacgataaggatcgcgttcttc	DST
**2.07**	AcrB_F948I_FW	ccttatcgtcgaaATCgccaaagacttgatggataaag	DST
**2.08**	AcrB_F948I_RV	ctttggcGATttcgacgataaggatcgcg	DST
**2.09**	AcrB_D951A_FW	gaattcgccaaaGCAttgatggataaagaaggtaaagg	DST
**2.10**	AcrB_D951A_RV	ctttatccatcaaTGCtttggcgaattcgacgataag	DST
**2.11**	AcrB_D951N_FW	gaattcgccaaaAACttgatggataaagaaggtaaagg	DST
**2.12**	AcrB_D951N_RV	ctttatccatcaaGTTtttggcgaattcgacgataag	DST
**2.13**	AcrB_K955E_FW	cttgatggatGAAgaaggtaaaggtctgattgaagc	DST
**2.14**	AcrB_K955E_RV	cctttaccttcTTCatccatcaagtctttggcg	DST
**2.15**	AcrB_D951A_K955E_FW	cgccaaaGCAttgatggatGAAgaaggtaaaggtctgattg	DST
**2.16**	AcrB_D951A_K955E_RV	ccttcTTCatccatcaaTGCtttggcgaattcgacgataag	DST
**2.17**	AcrB(NheI)FW2	atatatGCTAGCgaccggtgcaaacg	DST
**2.18**	AcrB_E734R_FW	gattgatatcgaccagAGAaaagcgcaggcg	DST
**2.19**	AcrB(XhoI)RV2	atatatCTCGAGatgatgatcgacagtatggctg	DST
**3.01**	T7P	aaataaatacgactgacttaggg	DST
**3.02**	T7T	gctagttattgctcagcggtg	DST
**3.03**	AcrB-for3	aagattgagctgggtggtgag	DST
**3.04**	AcrB-for7	ttctcggcgttgtcctcttctc	DST

Table S8. Residue-wise contribution to the free energy of binding ($\Delta G_{binding}$) of the BDM91531 inhibitor in wild-type and mutant forms of AcrB, calculated using MM/GBSA analysis. Values are reported in kcal/mol*.

Residue	Total	404	405	407	408	411	412	437	438	441	442	443	445	446	449	478	481	482	892	940	943	944	947	948	951	955
**wt**	-41.9	-2.9	-0.7			-1.4			-1.3	-1.6	-2.8		-2.3	-1						-0.9	-1	-0.9	-5.2	-2.2	-2.6	
**Q437E**	-47.0	-3.5	-0.6	-0.6		-2.3		-4.3	-1.5		-1.6		-2.7		-1.1	-0.6					-1	-2	-5.6	-0.7	-2.9	
**K955E**	-46.5					-2.9			-1.6	-0.8	-3.5	-0.6	-3.7	-0.8	-1.3	-0.6	-0.5	-1		-1.3	-0.9	-0.7	-6.5	-1.1	-1.8	
**D951A_K955E**	-44.6	-2.4				-2.3			-1.7	-0.7	-2.1		-2.9	-0.6	-1.1					-0.6	-1	-0.9	-4.1	-1.1	-0.9	-3.4
**Q437E_D951A**	-44.8	-3.3	-0.8			-2.2			-2.5	-0.8	-2.5		-3.5	-1.2	-0.9	-0.5				-1.4	-0.9	-1.1	-4	-1.5	-0.7	
**F948A**	-34.2	-1	-0.7	-0.5		-1.9	-0.6		-1.9		-1.3		-1.5		-1.3	-0.6						-1.9	-3.1		-1.4	
**D951A**	-34.3			-3.4	-3.3	-1.7			-1.5	-1.2	-1.3		-2.9						-1.1		-1	-1.4				
**E947A**	-40.8					-2.6			-1.3	-0.7	-2.9	-0.5	-3.4	-1.1	-1.1	-0.8		-0.9		-1.3	-0.8	-0.6	-1.4	-0.9	-1.5	

*Heatmap color scale ranges from white to green, with deeper green indicating a greater residue contribution to the $\Delta G_{binding}$ in each AcrB variant

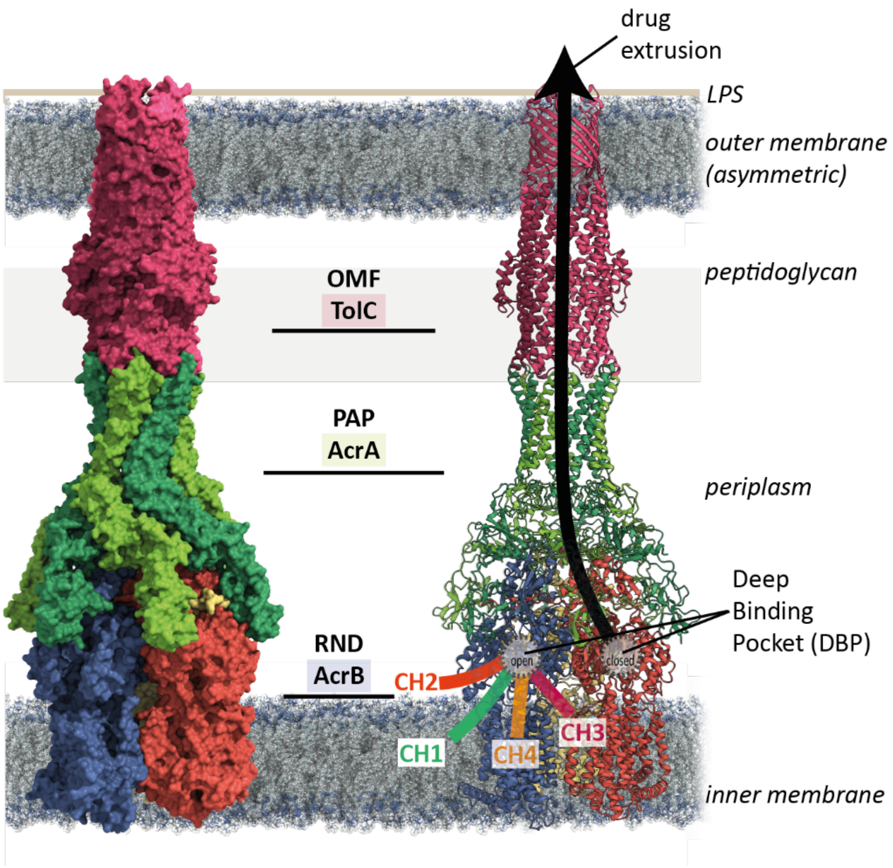

Fig. S1. Architecture of the tripartite AcrAB-TolC pump assembly. The homotrimeric inner membrane (IM) transporter AcrB (blue, red, yellow) is connected to the trimeric outer membrane (OM) channel TolC (pink) via the hexameric periplasmic adapter protein (PAP) AcrA (green). Substrates can enter through different channels into AcrB (CH1-CH4, indicated with different colors), where it binds to the DBP (grey encircled area). Upon closure of the DBP in the O protomer (red), the drug is then transported across the outer membrane to the outside (black arrow).

E. coli WT BW25113

Pyridomycin (µg/mL) \ BDM91531 (µM)	64	32	16	8	4	2	1	0.5	0.3	0.1	0
64	0	0	0	0	0	0	0	0	0	-1	-1
32	0	0	0	0	0	0	0	0	0	4	0
16	0	0	0	0	0	0	0	5	25	40	31
8	0	0	0	0	0	0	7	52	77	81	77
4	0	0	0	0	0	6	57	82	92	98	92
2	0	0	1	1	9	62	83	91	103	104	96
1	0	1	1	29	63	91	91	94	101	99	95
0.5	0	17	52	104	102	105	102	104	95	101	105
0.3	14	84	119	136	114	113	105	111	97	97	109
0	22	163	148	135	125	118	104	109	104	107	106

E. coli ΔacrAB

Pyridomycin (µg/mL) \ BDM91531 (µM)	64	32	16	8	4	2	1	0.5	0.3	0.1	0
64	0	0	0	0	0	0	0	0	0	0	0
32	0	0	0	0	0	0	0	0	0	0	0
16	0	0	0	0	0	0	0	0	0	0	0
8	0	0	0	0	0	0	0	0	0	0	0
4	0	0	0	0	0	0	0	0	0	0	0
2	0	0	0	0	0	0	0	0	0	0	0
1	0	1	1	1	1	1	1	1	1	1	1
0.5	0	4	23	43	56	63	64	70	77	77	71
0.3	9	78	100	102	92	99	93	95	99	92	90
0	17	126	120	109	105	105	101	102	103	98	96

E. coli WT BW25113

Oxacillin (µg/mL) \ BDM91531 (µM)	64	32	16	8	4	2	1	0.5	0.3	0.1	0
128	0	0	0	0	0	0	83	93	92	93	96
64	0	0	0	0	1	8	95	92	95	96	89
32	0	0	0	0	1	89	91	92	92	93	95
16	0	0	1	0	41	97	99	95	97	97	93
8	0	0	2	1	103	104	98	95	103	99	98
4	1	1	1	108	109	106	102	99	102	96	101
2	0	0	61	123	114	111	104	101	101	100	101
1	0	29	128	125	118	110	105	106	101	103	105
0.5	3	140	138	129	122	123	109	105	110	104	106
0	16	158	143	134	128	119	118	111	113	113	111

E. coli ΔacrAB

Oxacillin (µg/mL) \ BDM91531 (µM)	64	32	16	8	4	2	1	0.5	0.3	0.1	0
128	0	0	0	0	0	0	0	0	0	0	0
64	0	0	0	0	0	0	0	0	0	0	0
32	0	0	0	0	0	0	0	0	0	0	0
16	0	0	0	0	0	0	1	1	0	0	1
8	0	1	1	1	1	1	1	1	1	1	1
4	0	1	1	1	1	1	1	1	1	1	1
2	1	1	1	1	1	1	1	1	1	1	1
1	0	1	2	3	15	53	67	69	69	65	64
0.5	1	61	112	102	97	94	93	98	95	92	93
0	24	132	130	123	116	110	115	109	108	106	106

Fig. S2. Checkerboard assays combinations of pyridomycin (top) and oxacillin (bottom) with BDM91531, as performed on WT *E. coli* BW25113 (left) and an isogenic lacking *acrA* and *acrB* (*E. coli* BW25113 Δ*acrAB*, right). Bacteria were cultured with antibiotic/EPI mixes for 5 h followed by a viability assessment using resazurin. Values indicate the relative percentage of resazurin turnover as measured by fluorescence. Data are an average (range) of 3 independent biological replicates.

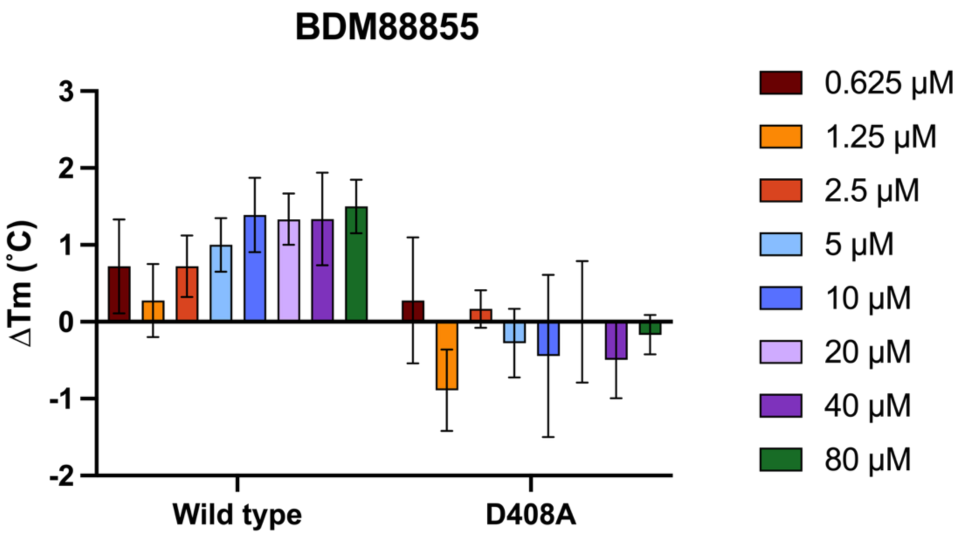

Fig. S3. D408 is essential for BDM88855 binding and activity. (**A**) Differential scanning fluorimetry of AcrB D408A in dependence on BDM88855 concentration. Solubilized AcrB wildtype or substitution variant D408A were incubated in presence of 0 - 80 µM BDM88855 during cysteine-reactive fluorophore CPM-based melting temperature determination. Thermal shifts (ΔT_M) from at least three DSF measurements in presence of BDM88855 are shown as deviations from the respective apo protein melting temperature. The average values are shown in the bar chart with standard deviation. Wildtype AcrB shows thermal stabilization in presence of the inhibitor, indicating binding. The inactive variant D408A was not thermal stabilized in presence of the inhibitor indicating lack of binding. CPM: 7-diethylamino-3-(4-maleimidophenyl)-4-methylcoumarin.

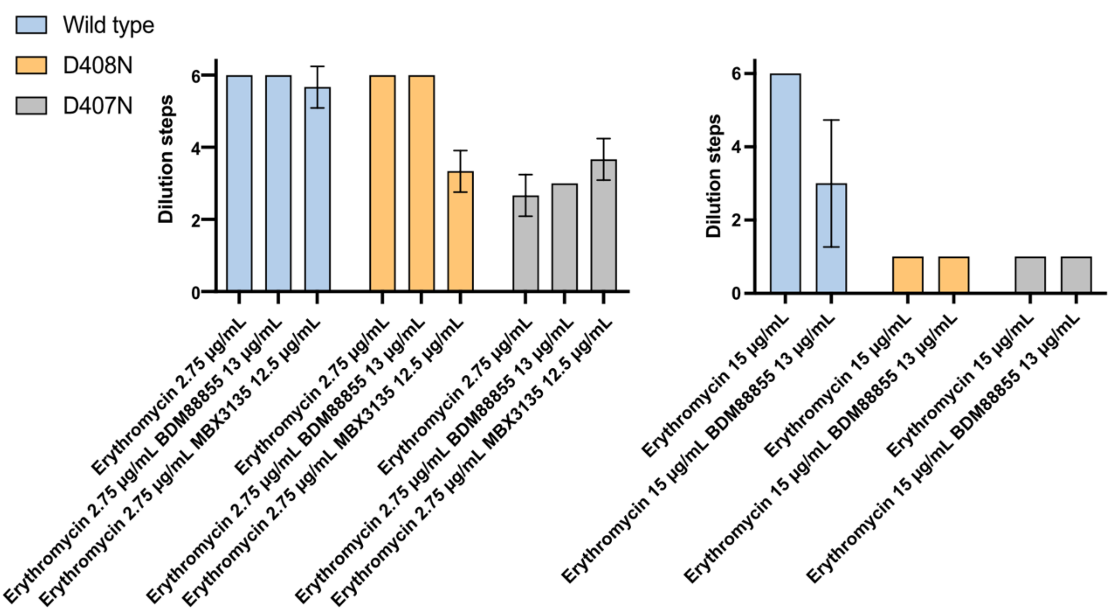

Fig. S4. Erythromycin susceptibility growth assays with *E. coli* BW25113Δ*acrB* complemented with WT, D407N, and D408N AcrB. All experiments were performed in biological triplicates. The numbers on the ordinate indicate the number of dilution steps where growth was observed on the growth agar plates. AcrB_D408N confers residual resistance compared to the inactive AcrB_D407N variant[4]. The presence of BDM88855 (13 µg mL^{-1}) does not affect the growth of cells harbouring D408N, whereas the presence of the inhibitor MBX3135 (12.5 µg mL^{-1}), which binds to the deep binding pocket, eliminates its activity. Wildtype AcrB appears not to confer inhibition at the low erythromycin concentration (2.75 µg mL^{-1}), a condition which was specifically used to show the residual AcrB_D408N activity. To show BDM88855 inhibition of the wildtype AcrB, the same experiment was conducted using a higher erythromycin concentration (15 µg mL^{-1}).

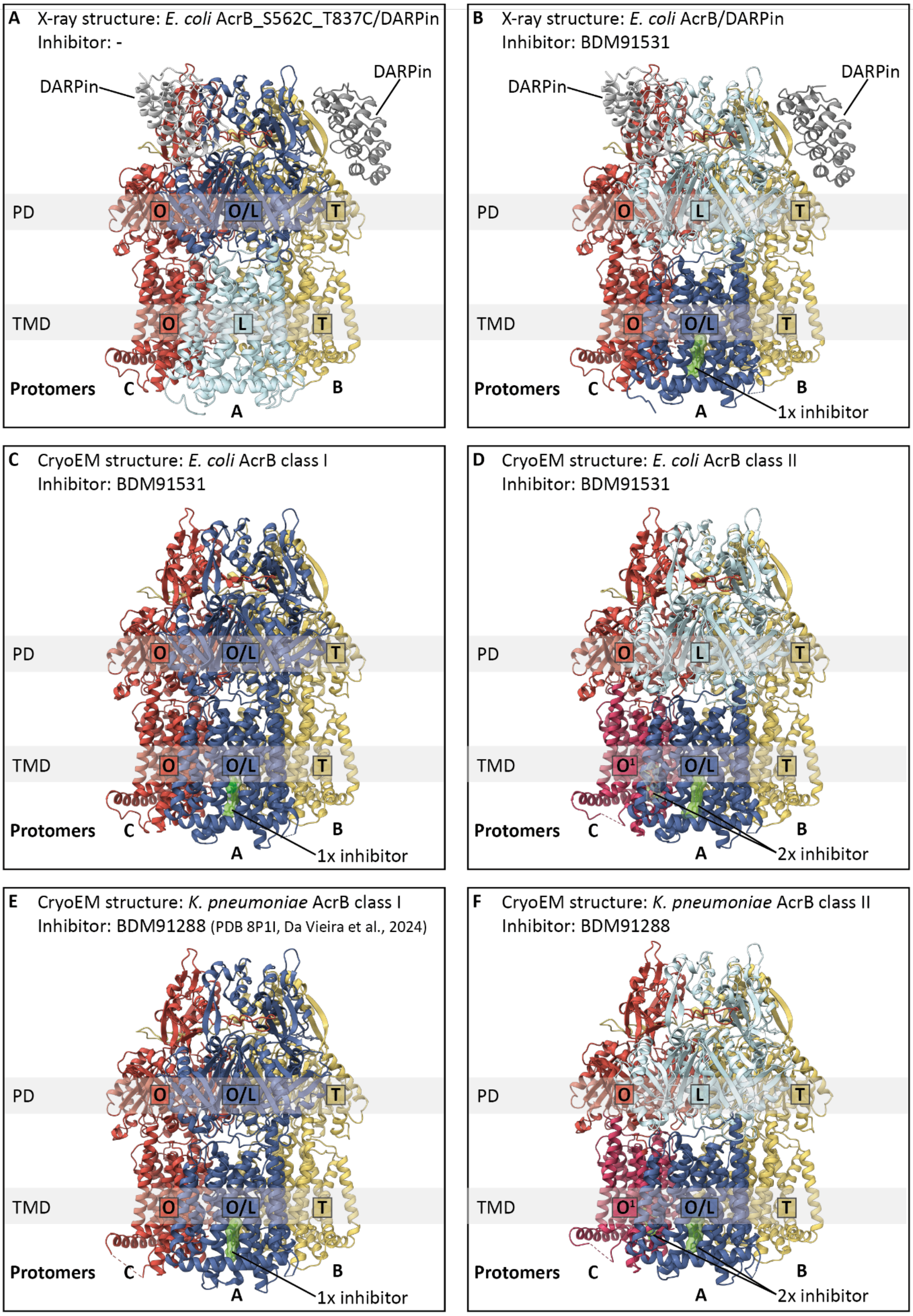

Fig. S5. Overview of all presented AcrB structures and their different conformational states in this study. The cryoEM *K. pneumoniae* AcrB class I structure (PDB 8P1I) has been published in [5]. O^1 indicates the O state except for TM*s* 1, 4, 5, 8-11, which adopt a transitional conformation within the binding region to accommodate the inhibitor, detailed in Fig. S8.

***E. coli* AcrB in complex with BDM91531**

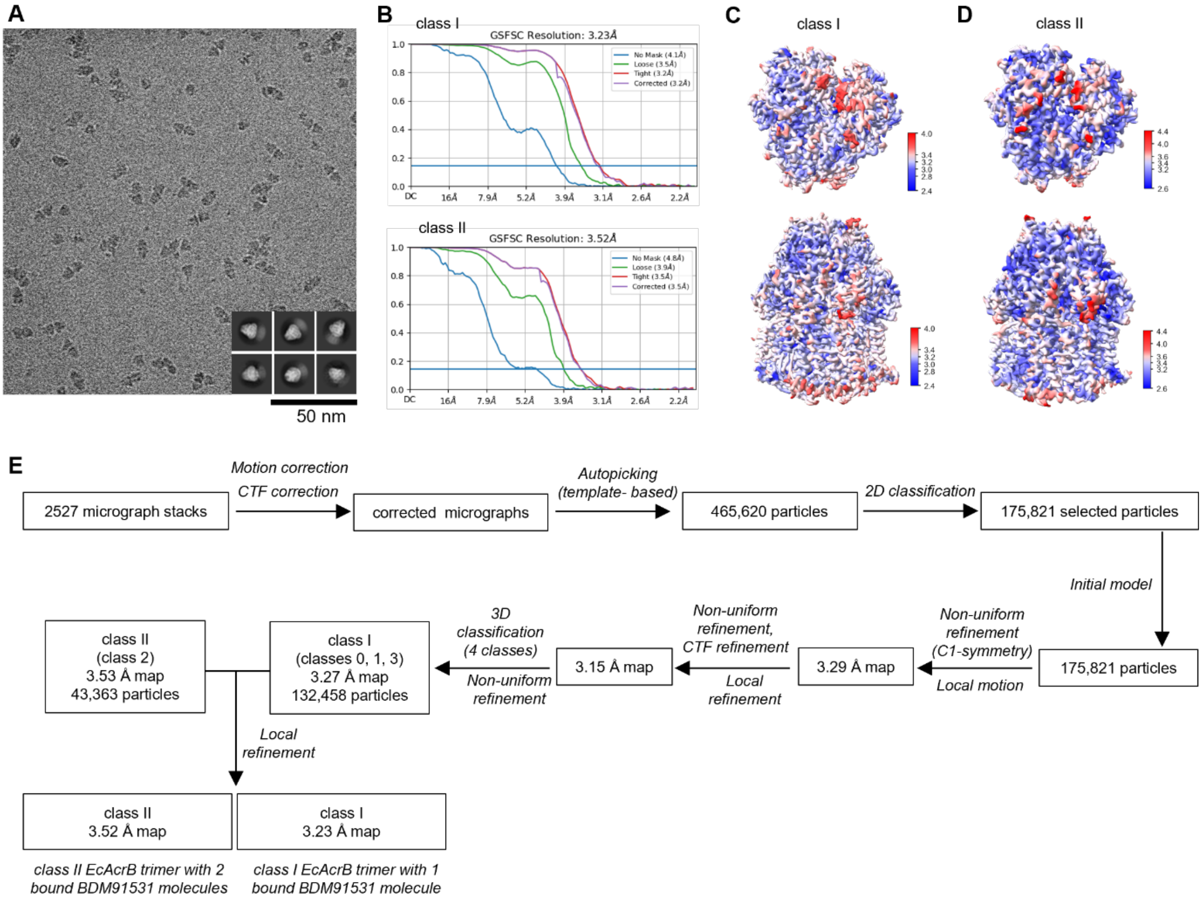

Fig. S6. Overview of cryo-EM processing of *E. coli* AcrB in complex with BDM91531. (**A**)**.** Exemplary micrograph of the *Ec*AcrB sample with BDM91531 at -3.0 µm defocus with representative 2D class averages used for template picking. (**B**) FSC curves of *Ec*AcrB class I and II. (**C**) and (**D**) Top views and side views of *Ec*AcrB density maps (C1 symmetry) for class I and II with global resolutions of 3.23 Å and 3.52 Å, respectively. (**E**) Schematic processing overview with cryoSPARC v4.0 of the *Ec*AcrB sample with bound BDM91531 inhibitor.

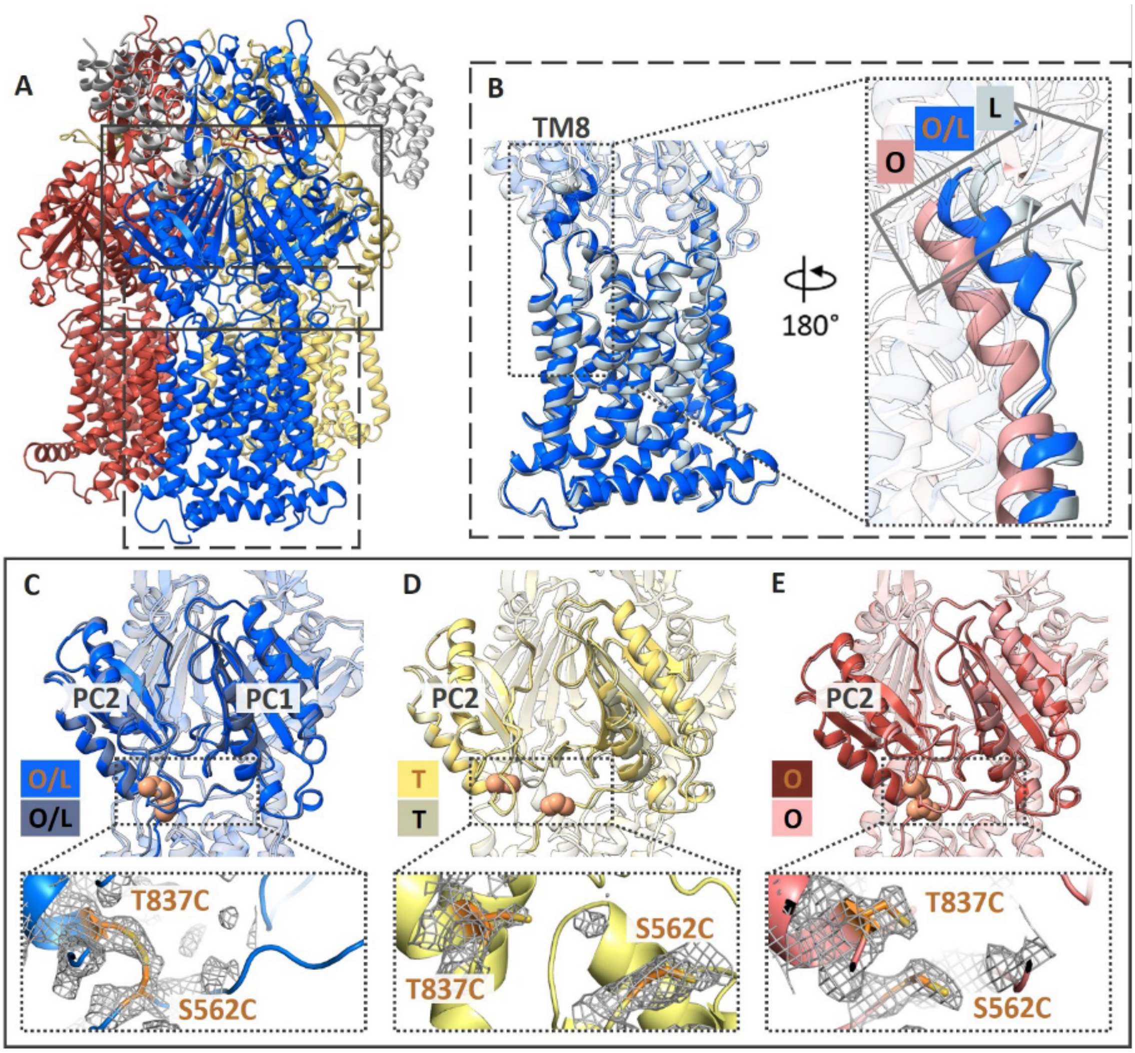

Fig. S7. Structural analysis of the AcrB S562C_T837C cross-linked variant. This variant shows reduced efflux activity in its cross-linked state, and its activity can be recovered upon addition of reducing agents [6]. S562 resides on TM7, and T837 on the PC2 subdomain. (**A**) Side view of the TM7/PC2 cross-linked structure. Protomer A (blue) is in front, the protomers B and C (yellow and red, respectively) are in the back. The two DARPin chaperones, which bind to protomer A (blue) and B (yellow), but not to protomer C (red), are indicated in grey color. The transmembrane domain (TMD) region indicated with a dashed-lined box is displayed in a detailed view in (**B**), whereas the solid-lined boxed periplasmic region of AcrB is shown in detail in (**C**), (**D**), and (**E**). (**B**) Detailed view at the TMD of protomer A of the AcrB_S562C_T837C cross-linked structure (colored in blue). The structure is superposed on the AcrB wildtype protomer A in the L state (in steel blue, PDB: 4DX5). Whereas the TMD of both structures appear congruent, the conformation of TM8 deviates as shown in the inset. Here, the superimposition of the TM8 conformations between the L state (steel blue), the O state of wildtype AcrB (red) and the AcrB_S562C_T837C cross-linked structure (blue) show that the latter adopts a T-to-O intermediate conformational state. (**C**-**E**) Side view on the PC1 and PC2 subdomains (in front in solid colors) of the AcrB_S562C_T837C cross-linked periplasmic porter domain. The protomer structures of the cross-linked AcrB are superimposed on the non-cross-linked wildtype protomer structures shown in lighter color. The Cys-substituted S562 and T837 are shown as spheres in protomer A (blue), B (yellow), and C (in red). The B and C protomers adopts the T and O state respectively. Protomer A adopts an intermediate O/L state, as shown in (**C**). The dotted boxed regions are shown enlarged in the insets below. Here the $2F_o$-F_c densities are indicated as grey mesh at 1 σ. The formation of the Cys-crosslink is apparent for protomer A (C), but not for the Protomers B (yellow) and C (red) in the T and O state, respectively.

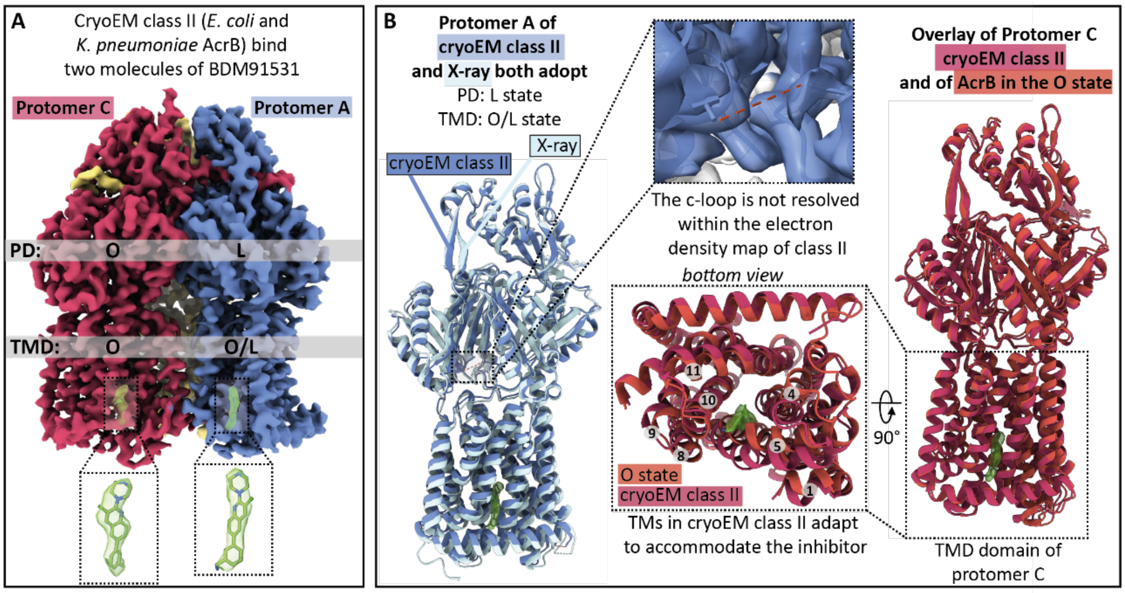

Fig. S8. Simultaneous BDM-inhibitor binding in two protomers of the AcrB trimer (cryoEM class II). The class II AcrB trimer is specifically shown only for the *E. coli* AcrB co-structure, as the conformational characteristics are congruent to the *K. pneumoniae* AcrB co-structure. RMSD values for comparison of the different states can be found in Table S4 (**A**) Two molecules of the BDM-inhibitor bind to protomer A (dark blue) and protomer C (pink) simultaneously. The PDs of protomer A and C adopt the L and O conformation, respectively. The TMDs of protomer A and C adopt the O/L and O conformation, respectively. (**B**) Overlay of protomer A of cryoEM class II (dark blue) with protomer A of the X-ray BDM91531 bound structure (light blue) shows that both, the PD and TMD are congruent in the L state and O/L state, respectively. The c-loop within protomer A is not resolved within the electron density maps of cryoEM class II. Overlay of protomer C cryoEM class II (pink) with the O state (red). The TMD of protomer C class II is in the O state, except for TMs 1, 4, 5, 8-11, which adopt a transitional conformation within the binding region to accommodate the inhibitor (bottom view).

K. pneumoniae AcrB class II in complex with BDM91288

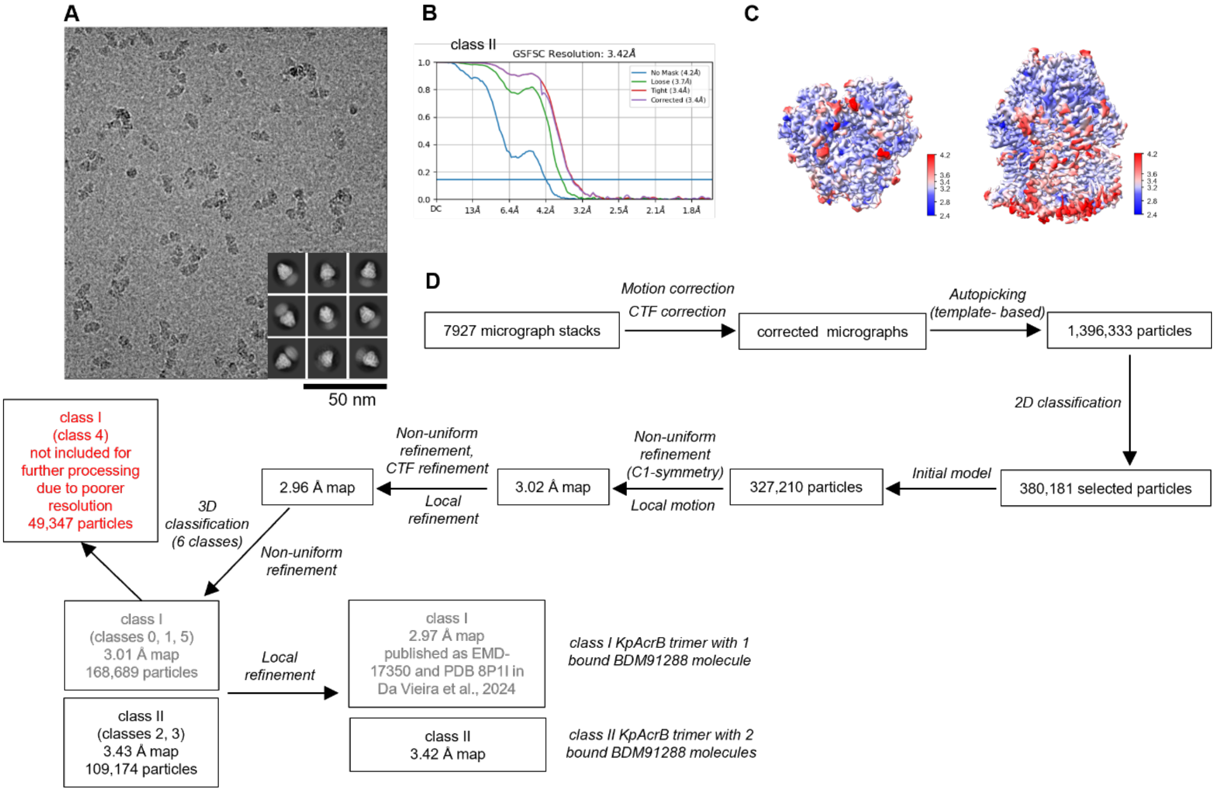

Fig. S9. Overview of cryo-EM processing of *K. pneumoniae* AcrB in complex with BDM91288. (**A**) Exemplary micrograph of the *Kp*AcrB sample with bound BDM91288 at -3.5 µm defocus with representative 2D class averages used for template picking. (**B**) FSC curve of *Kp*AcrB. (**C**)Top view and side view of the *Kp*AcrB class II density map (C1 symmetry) with an overall resolution of 3.42 Å.

(**D**) Schematic processing overview with cryoSPARC v4.1.2 of the *Kp*AcrB construct with bound BDM91288 inhibitor. The resulting *Kp*AcrB class I (EMD-17350, PDB 8P1I) has already been published in [5].

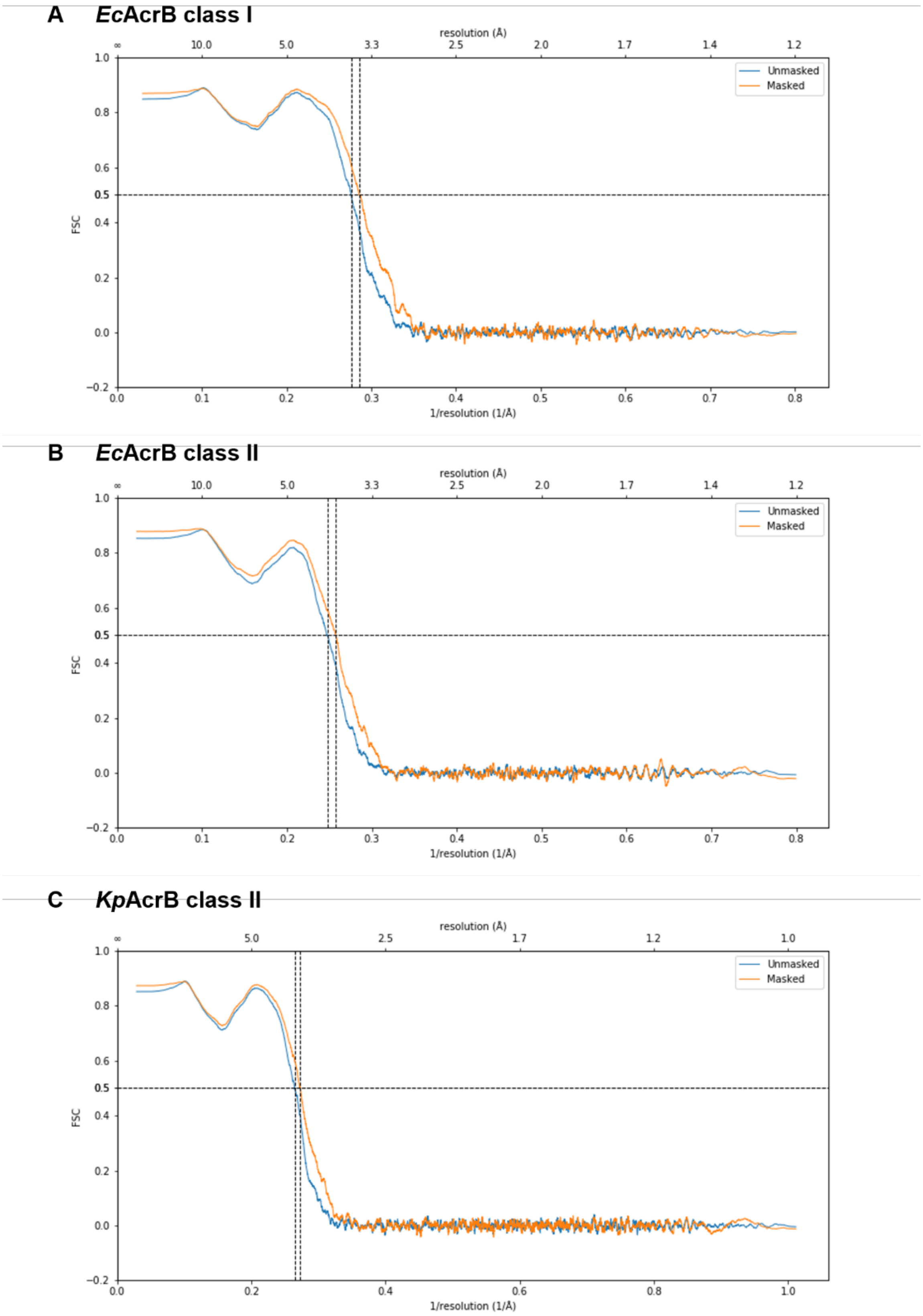

Fig. S10. Map-Model FSC curves of *Ec*AcrB class I and II and *Kp*AcrB class II. (**A**) Map-model FSC of *Ec*AcrB class I. (**B**) Map-model FSC of *Ec*AcrB class II. (**C**) Map-model FSC of *Kp*AcrB class II.

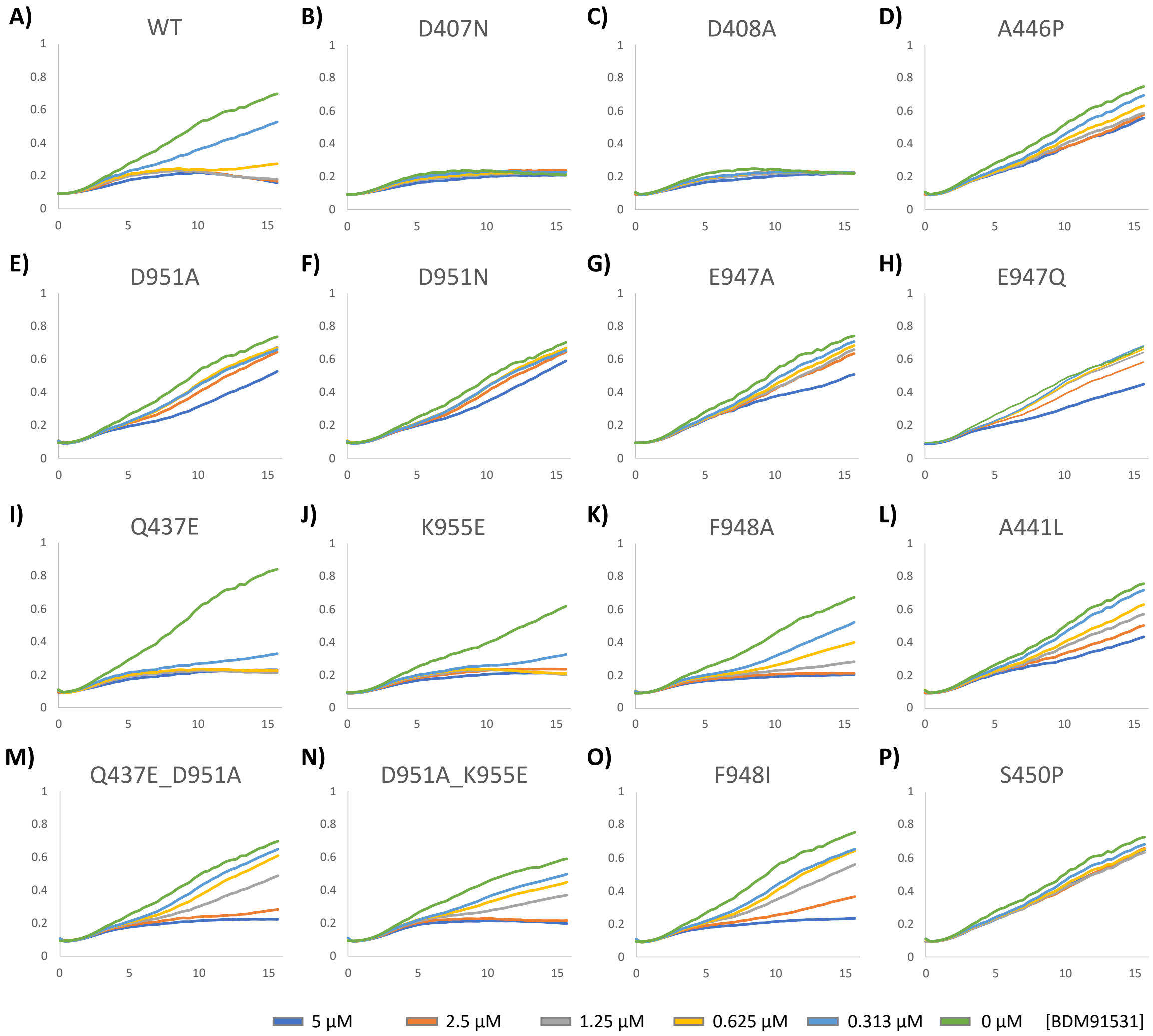

Fig. S11. Phenotypical characterization against a BDM91531 titration series. *E. coli* BW25113*ΔacrB*/ pET24a_*acrB* expressing the indicated *acrB* variants (WT (**A**), D407N (**B**), D408A (**C**), A446P (**D**), D951A (**E**), D951N (**F**), E947A (**G**), E947Q (**H**), Q437E (**I**), K955E **(J**), F948A (**K**), A441L (**L**), Q437E_D951A (**M**) , D951A_K955E (**N**), F948I (**O**) and S450P (**P**)) were incubated in LB kanamycin (50 µg/ml) in the presence of 16 µg/ml erythromycin in combination with a titration series of 5 µM, 2.5 µM, 1.25 µM, 0.625 µM, 0.313 µM or 0 µM BDM91531 at 37°C for 16h. Relative absorption at 600 nm (in AU, y-axis) was determined every 20 min and are plotted against the incubation time (in h, x-axis). The figure shows the representative results of one of three measurement days (biological replicates). In contrast to the WT (**A**), growth of bacteria harbouring the A446P (**D**), S450P (**P**), E947A/Q (**G** and **H**) or D951A/N (**E** and **F**) substitution variants was not (or only slightly) affected even at highest inhibitor concentrations. Interestingly, the introduction of the acidic substitutions Q437E and K955E at the putative inhibitor entry site resulted in hypersensitive phenotypes of the single substitution variants (**I** and **J**) when compared to the WT and for the double substitution variants (Q437E_D951A (**M**) and D951A_K955E (**N**)) when compared to the D951A variant (**E**), indicating that the function of D951 within the cytoplasmic rim can (partially) be replaced by nearby anionic substitutions.

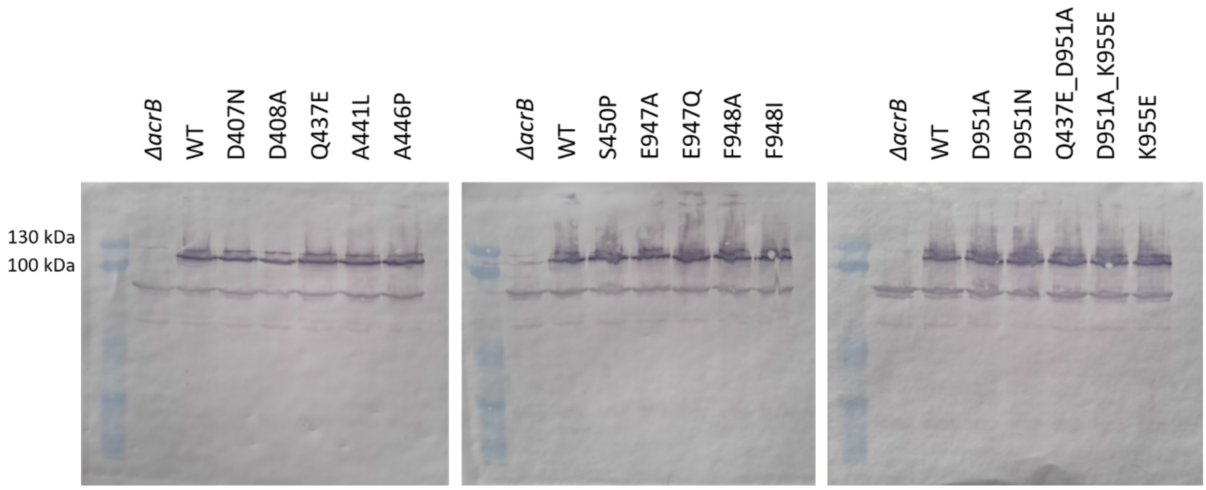

Fig. S12. Similar expression levels of tested AcrB mutants. *E. coli* BW25113*ΔacrB* expressing the indicated *acrB* mutants and wildtype (WT, positive) and empty vector (*ΔacrB*, negative) controls were lysed and subjected to SDS-PAGE and Western blot analysis. AcrB was probed with an α-AcrB polyclonal primary antibody (rabbit) in combination with an alkaline-phosphatase-conjugated anti-rabbit secondary antibody as explained in the Materials and Methods section. AcrB specific bands of comparable intensities were observed for all variants tested (with an approx. electrophoretic mobility of 113 kDa).

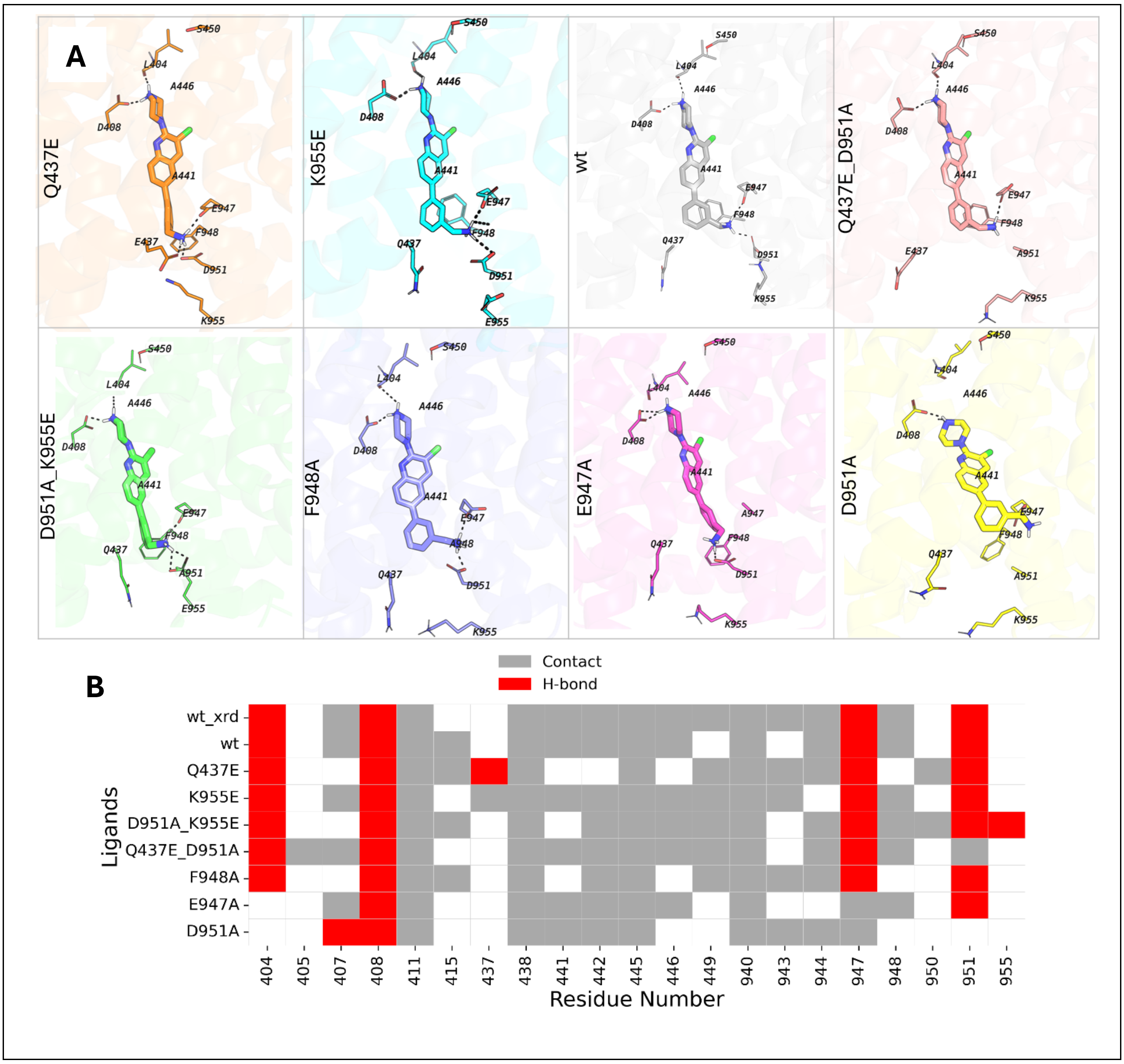

Fig. S13. BDM91531 binding in AcrB wild-type and mutants. (A) MD-derived representative clusters of BDM91531 (sticks) bound to various AcrB mutants (transparent cartoon), with ligands color-coded by mutant. **(B)** Heatmap of residue-level interactions between BDM91531 and AcrB variants: grey color indicates contacts, red color indicates hydrogen bonds (Maestro Ligand Interaction toolkit). Residue numbers are on the x-axis; AcrB variants on the y-axis. *wt_xrd* refers to the reference experimental X-ray structure.

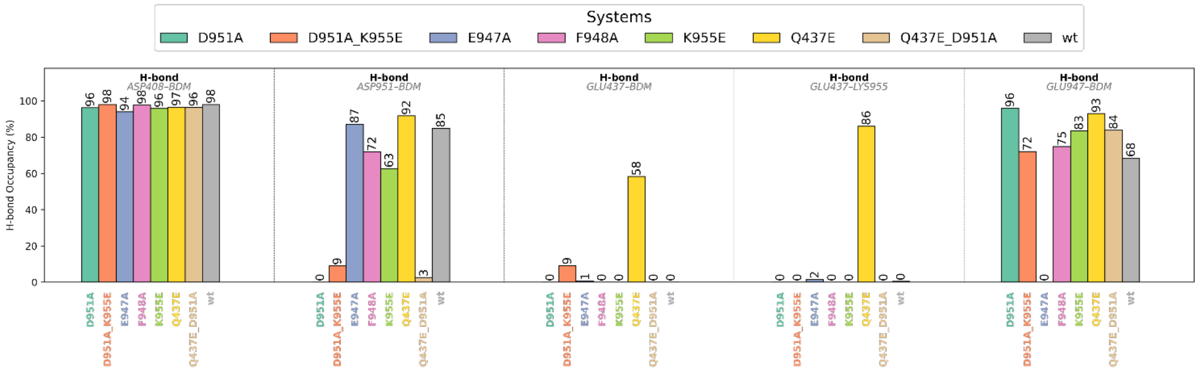

Fig. S14. Hydrogen bond occupancies between BDM91531 and AcrB variants. Bar plot showing key hydrogen bond interactions identified in MD simulations of BDM91531 bound to different modelled AcrB mutants. Each group of bars corresponds to a specific H-bond interaction, and each bar within a group represents one AcrB variant (system). Systems are color-coded as indicated in the legend above. System names are labeled along the x-axis, and the y-axis shows the occupancy percentage (%).

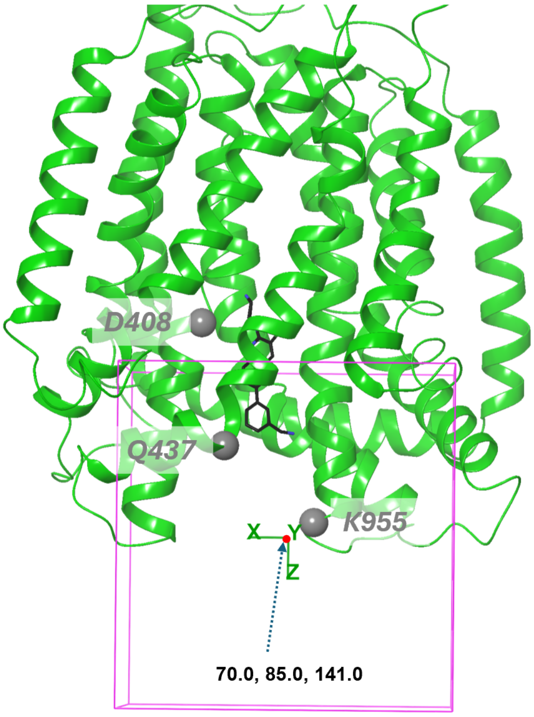

Fig. S15. Docking Grid employed for the BDM91531 accessibility. Ensemble docking calculations for accessibility were conducted separately in the cytoplasmic gate of the L-state conformation of AcrB, with a grid box centred at (70.0, 85.0, 141.0) Å for the lower channel, each with dimensions of 33 × 32 × 35 Å along the x, y, and z axes, respectively, oriented relative to the bilayer normal. This grid was shifted downward to avoid the D407/408 anchor site based on a maximum ligand extension of ~18 Å observed in MD simulations to ensure complete exploration of ligand interaction. The C_α atoms at positions 408, 437, and 955 are depicted as grey spheres.

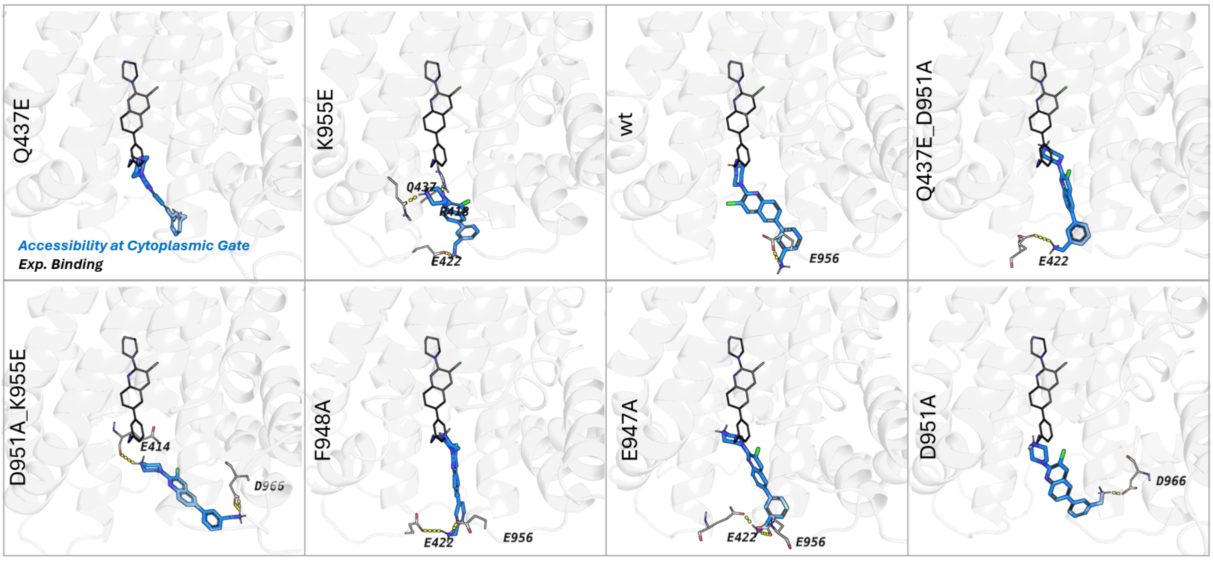

Fig. S16. Predicted accessible entry routes through cytoplasmic gate of BDM91531 in AcrB mutants. In blue (carbon) poses are for cytoplasmic gate whereas experimental pose of BDM91531 in the AcrB wildtype. XRD structure provided as black lines for reference. H-bonds with the poses of the cytoplasmic gate are shown with the corresponding residue.

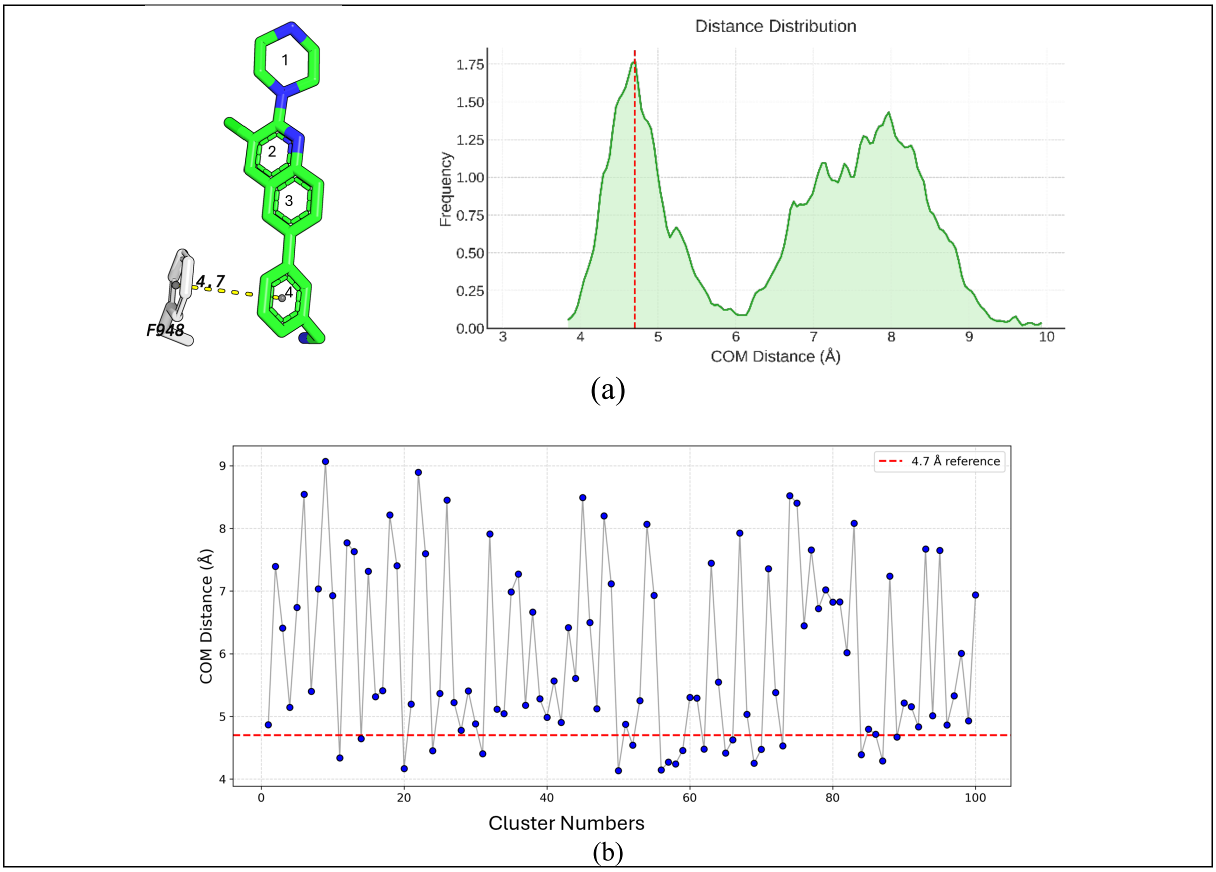

Fig. S17. π–π interaction analysis between BDM91531 and F948 in WT complex. (a) Distribution of center-of-mass (COM) distances between the terminal aromatic ring of BDM91531 (ring 4) and the aromatic ring of F948 across three independent wildtype replicas. The red dashed line indicates the reference distance (4.7 Å) from the experimental structure (left). **(b)** COM distances for 100 representative clustered frames, clustered from the 100 ns WT simulation trajectory across three replicas. Corresponding structural frames are provided in the Supporting Information (Zenodo).

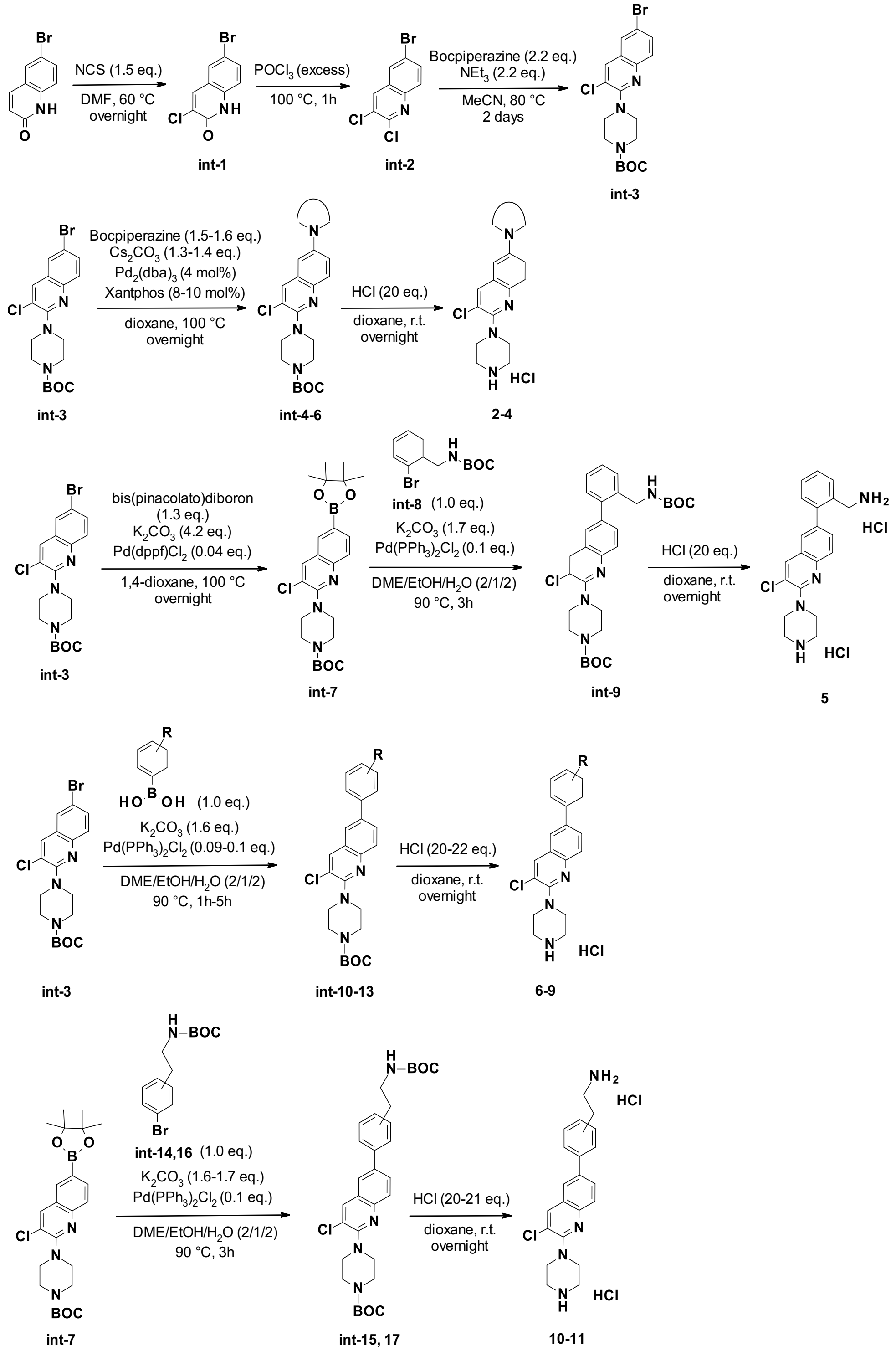

Fig. S18. Chemical scheme for the synthesis of compounds 2 to 11. Abbreviations: Boc, tert-butoxycarbonyle; dba, dibenzylideneacetone; DME, dimethoxyethane; DMF, dimethylformamide; dppf, 1,1'-bis(diphenylphosphino)ferrocene; MeCN, acetonitrile; NCS, *N*-chlorosuccinimide; r.t., room temperature; Xantphos, (9,9-Dimethyl-9*H*-xanthene-4,5-diyl)bis(diphenylphosphane).

References

1. Meng, E. C. *et al.* UCSF ChimeraX: Tools for structure building and analysis. *Protein Science* **32**, e4792 (2023).
2. Eicher, T. *et al.* Transport of drugs by the multidrug transporter AcrB involves an access and a deep binding pocket that are separated by a switch-loop. *Proc. Natl. Acad. Sci. U.S.A.* **109**, 5687–5692 (2012).
3. Plé, C. *et al.* Pyridylpiperazine-based allosteric inhibitors of RND-type multidrug efflux pumps. *Nat Commun* **13**, 115 (2022).
4. Seeger, M. A., von Ballmoos, C., Verrey, F. & Pos, K. M. Crucial Role of Asp408 in the Proton Translocation Pathway of Multidrug Transporter AcrB: Evidence from Site-Directed Mutagenesis and Carbodiimide Labeling. *Biochemistry* **48**, 5801–5812 (2009).
5. Vieira Da Cruz, A. *et al.* Pyridylpiperazine efflux pump inhibitor boosts in vivo antibiotic efficacy against K. pneumoniae. *EMBO Molecular Medicine* 93–111 (2023) doi:10.1038/s44321-023-00007-9.
6. Seeger, M. A. *et al.* Engineered disulfide bonds support the functional rotation mechanism of multidrug efflux pump AcrB. *Nat Struct Mol Biol* **15**, 199–205 (2008).
